# mRNA-based generation of marmoset PGCLCs capable of differentiation into gonocyte-like cells

**DOI:** 10.1016/j.stemcr.2023.08.006

**Published:** 2023-09-07

**Authors:** Musashi Kubiura-Ichimaru, Christopher Penfold, Kazuaki Kojima, Constance Dollet, Haruka Yabukami, Katsunori Semi, Yasuhiro Takashima, Thorsten Boroviak, Hideya Kawaji, Knut Woltjen, Aki Minoda, Erika Sasaki, Toshiaki Watanabe

**Affiliations:** 1 Central Institute for Experimental Animals, 3-25-12 Tonomachi, Kawasaki-ku, Kawasaki 210-0821, Japan; 2 Division of Molecular Genetics & Epigenetics, Department of Biomolecular Science, Faculty of Medicine, Saga University, 5-1-1 Nabeshima, Saga 849-8501, Japan; 3 Department of Physiology, Development and Neuroscience, University of Cambridge, Downing Site, Cambridge, UK; 4 Wellcome Trust-Cancer Research UK Gurdon Institute, University of Cambridge, Cambridge, UK; 5 Centre for Trophoblast Research, University of Cambridge, Downing Site, Cambridge CB2 3EG, UK; 6 Wellcome Trust – Medical Research Council Stem Cell Institute, University of Cambridge, Jeffrey Cheah Biomedical Centre, Puddicombe Way, Cambridge CB2 0AW, UK; 7 National Center for Child Health and Development, Tokyo 157-8535, Japan; 8 Laboratory for Cellular Epigenomics, RIKEN Center for Integrative Medical Sciences, Yokohama, Kanagawa 230-0045, Japan; 9 Department of Life Science Frontiers, Center for iPS Research and Application (CiRA), Kyoto University, Kyoto 606-8507, Japan; 10 Research Center for Genome & Medical Sciences, Tokyo Metropolitan Institute of Medical Science, Tokyo 156-8506, Japan; 11 Preventive Medicine and Applied Genomics Unit, RIKEN Center for Integrative Medical Sciences, Yokohama, Kanagawa 230-0045, Japan; 12 Department of Cell Biology, Faculty of Science, Radboud Institute for Molecular Life Sciences, Radboud University, Nijmegen, the Netherlands

**Keywords:** germ cells, marmosets, primate, iPSCs, gonocytes, PGCLCs, development, DNA methylation, mRNA transfection, SOX17

## Abstract

Primate germ cell development remains largely unexplored due to limitations in sample collection and the long duration of development. In mice, primordial germ cell-like cells (PGCLCs) derived from pluripotent stem cells (PSCs) can develop into functional gametes by *in vitro* culture or *in vivo* transplantation. Such PGCLC-mediated induction of mature gametes in primates is highly useful for understanding human germ cell development. Since marmosets generate functional sperm earlier than other species, recapitulating the whole male germ cell development process is technically more feasible. Here, we induced the differentiation of iPSCs into gonocyte-like cells via PGCLCs in marmosets. First, we developed an mRNA transfection-based method to efficiently generate PGCLCs. Subsequently, to promote PGCLC differentiation, xenoreconstituted testes (xrtestes) were generated in the mouse kidney capsule. PGCLCs show progressive DNA demethylation and stepwise expression of developmental marker genes. This study provides an efficient platform for the study of marmoset germ cell development.

## Highlights

• An mRNA transfection-based PGCLC induction method is developed
• Marmoset PGCLCs are efficiently induced from iPSCs
• Marmoset PGCLCs differentiate into gonocyte-like cells in mouse kidneys
• Developmentally regulated expression and demethylation are recapitulated

## Graphical abstract

**Figure:**
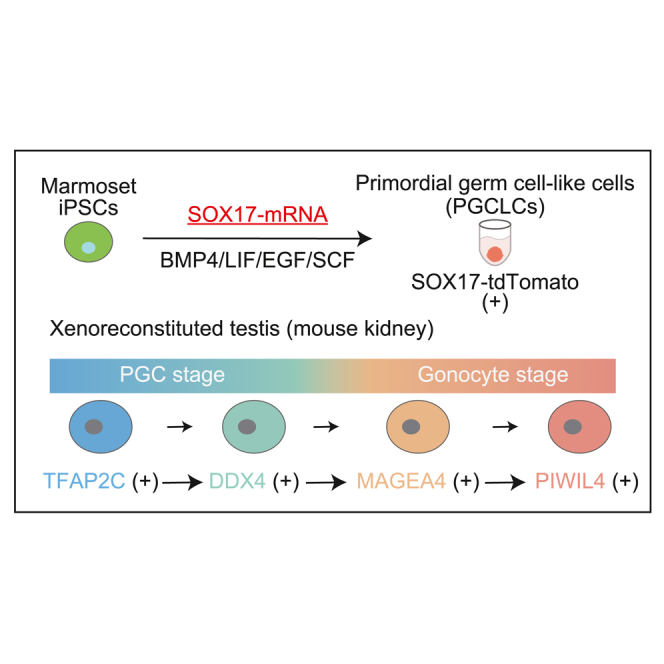

## Teaser

Watanabe et al. efficiently induced marmoset primordial germ cell-like cells (PGCLCs) using an mRNA transfection-based approach. PGCLCs further develop into gonocyte-like cells in the xenoreconstituted testes constructed under mouse kidney capsules. This system faithfully reproduced *in vivo* developmental processes (e.g., stage-specific expression of developmentally regulated genes and DNA demethylation).

## Introduction

Marmosets (*Callithrix jacchus*) are New World monkeys native to Brazil that are often used in biomedical research, particularly in brain science, owing to their small size, relative ease of handling, high reproductive ability, and close evolutionary relationship to humans. In addition to these characteristics, marmosets reach puberty within 1 year of birth. This relatively short period of sexual maturation makes marmosets an ideal model organism for studying primate germ cell development. However, the high cost of studies using marmosets limits the number of animal experiments. Therefore, to complement *in vivo* studies, it is important to develop a tractable marmoset germ cell developmental system using pluripotent stem cells (PSCs).

Several studies have reported the induction of primordial germ cell-like cells (PGCLCs) from PSCs in primates (humans, macaques, and marmosets) (Irie et al., 2015; Sakai et al., 2020; Sasaki et al., 2015; Sosa et al., 2018; Yoshimatsu et al., 2021). Although complete differentiation of PGCLCs into sperm or eggs has been successful in mice, it has not yet been achieved in any primate species. In humans, PGCLCs differentiate into gonocytes after a few months of the *in vitro* culture of xenoreconstituted testes (xrtestes) generated from human PGCLCs and mouse embryonic gonadal somatic cells (Hwang et al., 2020). Furthermore, in rhesus macaques, male PGCLCs differentiate into MAGEA4-positive gonocytes by homologous transplantation into adult testes and xenotransplantation into seminiferous tubules of mouse testes (Sosa et al., 2018). Because both extrinsic and intrinsic factors determine developmental speed, the duration of germ cell development in the PGCLC system is likely to be influenced by the *in vivo* development timetable. Although early embryonic development is delayed in marmosets (Phillips, 1976), they reach sexual maturation earlier than other primates. Therefore, the marmoset PGCLC system may be more feasible for recapitulating the entire primate germ cell development process. Although PGCLCs have been generated in marmosets, the reported method requires *a priori* transgene integration for the forced expression of *SOX17* and *PRDM1* (Yoshimatsu et al., 2021). Furthermore, there are no reports on the differentiation of PGCLCs into *MAGEA4*-positive gonocytes in marmosets.

In addition to examining germ cell development, this PGCLC-initiated germ cell developmental system is useful for generating genetically modified animals. This is enabled by gamete production using this system from genetically modified PSCs. All genetically modified marmosets generated to date have been produced from zygotes (Park and Sasaki, 2020). Cultured cell-based systems, such as the PGCLC-mediated system, enable the creation of animals with complex genetic modifications such as reporter gene knock-in and multiple modifications. Furthermore, a cell-based system ensures the production of the expected genetic modifications. Thus, establishing a germ cell developmental system from PGCLCs to produce functional gametes may accelerate primate research.

In this study, to develop a PGCLC-initiated germ cell developmental system in marmosets, we developed a novel mRNA transfection-based method to convert PSCs into PGCLCs. Furthermore, these PGCLCs were differentiated into a gonocyte-like state using a transplantation approach. Our results provide the basis for studying marmoset gametogenesis using PGCLCs.

## Results

### mRNA transfection-based induction of marmoset PGCLCs

To monitor the differentiation into PGC-like state, we inserted T2A-tdTomato into the C terminus of *SOX17* gene (*SOX17*-tdTomato [ST]) in one female (mRNA ST) and two male iPSC lines harboring CAG-EGFP (CE) transgenes (971 STCE and 972 STCE) (Figure S1A). We have previously reported mRNA transfection-based methods for marmoset induced PSC (iPSC) induction (Watanabe et al., 2019). To generate PGCLCs from iPSCs, we performed mRNA transfection. Because *SOX17* has been reported to have critical functions in PGCLC induction in humans (Irie et al., 2015; Kobayashi et al., 2017), we selected this mRNA for transfection. To alleviate the damage caused by mRNA transfection in cells, we transfected cells with interferon suppressors (vaccinia virus *E3*, *K3*, and *B18R* mRNAs) (Poleganov et al., 2015) and an apoptosis suppressor (mouse P53DD mRNA) (Hong et al., 2009).

**Figure 1:**
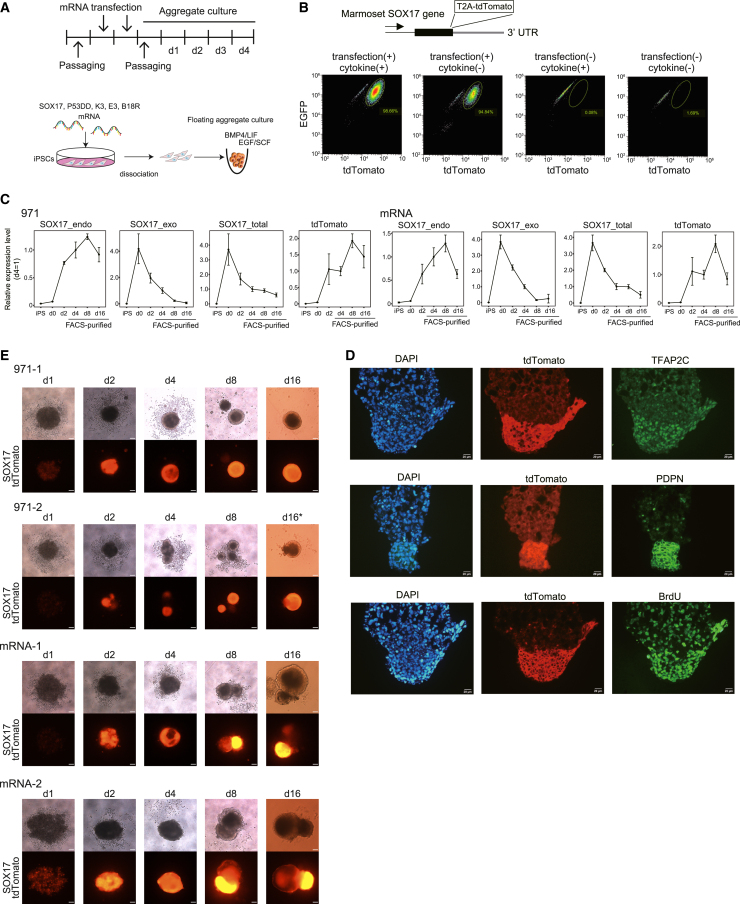
mRNA-based generation of PGCLCs from iPSCs (A) Schematic diagram for mRNA-mediated induction of PGCLCs from iPSCs. (B) Requirement of mRNA transfection and growth factors. A T2A-tdTomato cassette was inserted into the C terminus of the *SOX17* gene to monitor differentiation into PGCLCs. The four charts represent the results of induction with the presence/absence of mRNA transfection and cytokines (n = 1). (C) Time course qPCR analyses of total/exogenous/endogenous *SOX17* and tdTomato mRNA expression. PGCLC aggregates of two different cell lines (971 STCE and mRNA ST) were cultured for 16 days. A primer set detecting both exogenous and endogenous *SOX17* mRNAs was used for total *SOX17*. Error bars represent SD of the results of cells of different passage numbers (n = 2). (D) Immunohistochemical analyses of the day 4 PGCLC aggregates. The expression of PGC markers (*TFAP2C*, *PDPN*) and BrdU incorporation were examined in *SOX17*-tdTomato-positive cells. BrdU was added to the medium 48 h before the sampling. Scale bars, 20 μm. n = 1. (E) Time course examination of *SOX17*-tdTomato fluorescence (971 STCE and mRNA ST). Shown are PGCLC aggregates examined by the qPCR analyses in (C). Asterisk in 971-2: different PGCLC aggregates were shown for day 16 and the other stages. Scale bars, 100 μm.

After 2 successive days of transfection into 971 STCE iPSCs, the transfected cells were seeded onto low-attachment 96-well plates to form aggregates in medium containing LIF, EGF, stem cell factor (SCF), and BMP4 based on the procedures for human PGCLC induction (Figure 1A) (Hwang et al., 2020; Irie et al., 2015; Kojima et al., 2017; Sasaki et al., 2015; Yamashiro et al., 2018). Four days after making the aggregate, ST expression was observed in many cells in the aggregates (Figures 1B, S1B, and S1C), although the efficiency was somewhat varied among trials. ST expression was correlated with the endogenous *SOX17* expression (Figure 1C). Immunofluorescence analyses showed that these ST-positive cells co-expressed *TFAP2C* and *PDPN* (Figure 1D), suggesting that they are PGCLCs. In contrast, when mRNA transfection was omitted, ST expression was not observed (Figure 1B). This result indicates that mRNA transfection plays critical roles in PGCLC induction. Omission of growth factors slightly reduced the number of ST-positive cells and the level of ST fluorescence (Figure 1B). Thus, growth factors likely play some roles in PGCLC induction and/or maintenance in our induction system.

To examine the time course change, aggregate culture was continued with medium change every 4 days using a male iPSC line harboring *SOX17*-tdTomato transgene (971 STCE). ST expression was first observed 24 h after PGCLC aggregate formation (Figure 1E). A proportion of cells expressing ST fluorescence seemingly reached maximal levels at either day 4 or 8 (Figures 1E and S1D). Then, the proportions of the positive cells decreased at day 16 when we stopped the culture. However, a significant portion of cells still expressed ST, even on day 16. In ST-positive cells, exogenous *SOX*17 mRNAs were still observed at day 4, but they were barely detected by day 8 (Figure 1C), suggesting autonomous regulation of gene regulatory network at day 8. Proliferation of the ST-positive cell was observed on days 4, 8, and 16 (Figures 1D and S1E). The expression of PGC and pluripotent marker genes were observed in ST-positive cells during this period, although some decreases were observed on day 16 (Figure S1F).

The optimal number of mRNA transfections was determined to be two. Two successive days of transfections resulted in higher *NANOS3* expression in day 4 aggregates (d4_PGCLC) than did single transfection, but 3 successive day transfections did not result in increased *NANOS3* expression (Figure S2A). The optimal number of transfected cells was determined to be 50,000 cells per well in a 12-well plate (Figure S2B). Using an increased number of cells (100,000 cells) resulted in a decreased PGCLC induction efficiency, likely due to an insufficient number of mRNAs distributed into each cell when a larger quantity was used. In contrast to human PGCLC induction (Kobayashi et al., 2017), addition of *BLIMP1* mRNA to *SOX17* mRNA did not cause positive effects on marmoset PGCLC induction (Figure S2C). Therefore, *SOX17* mRNA was only used together with the determined conditions (2 successive days of transfection and 50,000 cells for transfection) for PGCLC generation in this study.

### Single-cell analysis of iPSCs and PGCLCs

**Figure 2:**
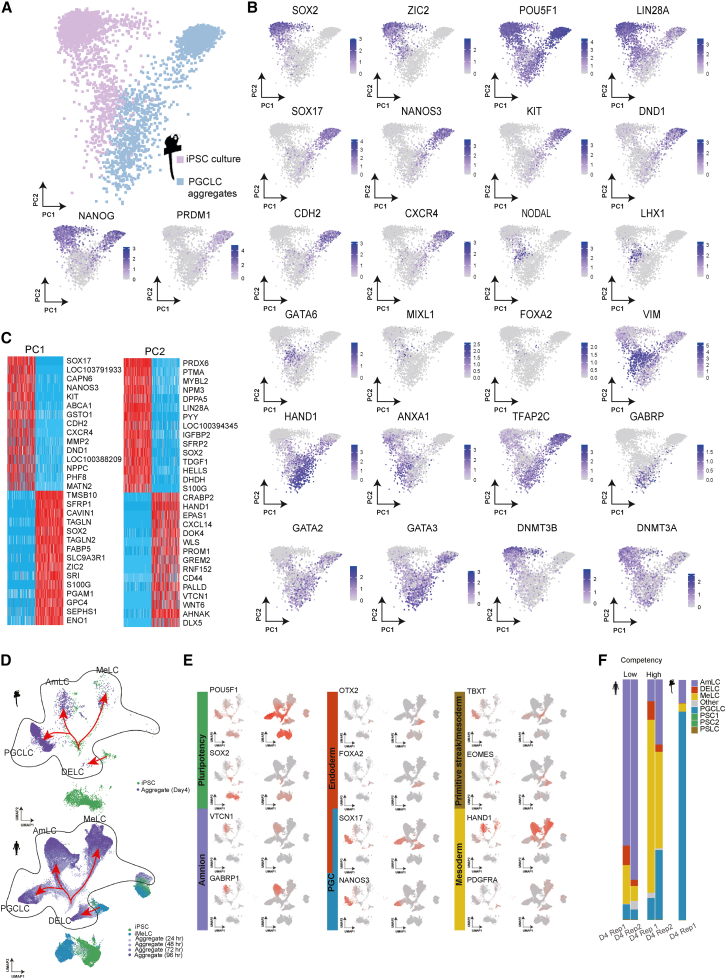
Gene expression analyses of marmoset PGCLCs induced from iPSCs (A) Single-cell analyses of iPSCs (n = 1) and PGCLCs (n = 1). (B) In addition to the iPSC (*SOX2* and *ZIC2*) and PGCLC markers (*SOX17*, *KIT*, *NANOS3*, and *DND1*), mesoendoderm-like cells (*NODAL* and *MIXL1*), a small number of endoderm-like cells (*SOX17* and *FOXA2*), mesenchymal-like cells (*HAND1*, *ANXA1*, and *VIM*), and amnion-like cells (*HAND1*, *TFA2PC*, and *GABRP*) were present. (C) Well-characterized iPSC markers (*SOX2* and *ZIC2*) and PGC markers (*SOX17*, *NANOS3*, *KIT*, and *DND1*) were found in the genes contributing to PC1. (D) Alignment to human dataset suggests four key lineages in our aggregate: PGCLCs, mesoderm-like cells (MeLCs), amnion-like cells (AmLC), and definitive endoderm-like cells (DELCs). (E) These subpopulations express appropriate markers identified in human systems (Tyser et al., 2021) or marmoset *in vivo* embryos (Bergmann et al., 2022). (F) The relative proportion of each cell type in PGCLC aggregates, which shows an approximately 70%–80% efficiency of PGCLC induction in our approach.

To examine whether induced cells showed gene expression typical of PGCs, single-cell RNA sequencing (scRNA-seq) libraries were constructed from iPSCs (mRNA iPSCs) and d4_PGCLC aggregates (derived from mRNA iPSCs) (Table S1). The *SOX2*-positive iPSC population constituted ∼80% of cells in the iPSC library, and the *NANOS3*-positive PGCLC population constituted 70%–80% of cells in the PGCLC library (Figures S3A and S3B). Principal-component analysis (PCA) was conducted by assembling all cells from both libraries. This analysis clearly separated the iPSC and PGCLC populations (Figure 2A), which were located on opposite sides of PC1. Well-characterized iPSC (*SOX2* and *ZIC2*) and PGC markers (*SOX17*, *NANOS3*, *KIT*, and *DND1*) were identified in the genes contributing to PC1 (Figures 2B and 2C). PC2 separated iPSC/PGCLC from other cell types, and iPSCs and PGCLCs were located on the upper part of the PC2 (Figure 2B). In the middle and lower parts of PC2, cells expressing well-known marker genes for somatic lineages (e.g., *NODAL*, *LHX1*, *HAND1*, *GABR*P, and *HOX* genes) were found (Figures 2B, 2C, and S4A).

To characterize cells that are present in our PGCLC aggregate, we aligned our dataset to a published dataset of PGCLC aggregates (Chen et al., 2019), which has been reported to include several cell types other than PGCLCs. Based on marker gene expressions reported in the studies of human and marmoset embryos (Bergmann et al., 2022; Tyser et al., 2021), four major populations of cells were identified in both marmoset and human PGCLC aggregates (Figures 2D and 2E): PGCLC (*SOX17* and *NANOS3*), amnion-like cells (AmLCs) (*HAND1*, *TFA2PC*, and *GABRP*), mesoderm-like cells (MeLCs) (*TBXT*, *EOMES*, *HAND1*, and *PDGFRA*), and a small number of definitive endoderm-like cells (DELCs) (*SOX17*, *OTX2*, and *FOXA2*). *SOX17* expression was, like humans, observed in DELCs as well as in PGCLCs; however, DELCs seemed to be already present in iPSCs rather than be newly induced. The rate of PGCLCs in our PGCLC aggregate was again estimated to be 70%–80% (Figure 2F). In marmoset PGCLCs, co-expression of *SOX17* and other germ cell marker genes (*BLIMP1/NANOS3/TFAP2C*) was observed (Figure S4B), suggesting conservation of the core PGC network. At the same time, we observed some differences in key transcription factor expression between marmoset and human PGCLCs: *POU5F1* was downregulated, while *NANOG* was upregulated in marmoset PGCLCs (Figure S4C). In somatic cells in the aggregates, *POU5F1* expression, although weak, was also observed (Figures 2B and 2E). In human systems (Castillo-Venzor et al., 2022), *POU5F1* expression is downregulated in other lineages, including DELCs, MeLCs, and AmLCs, although this downregulation itself is gradual. Therefore, some *POU5F1* may also persist early after their specification in marmosets. *NANOG*, a well-known iPSC and PGCLC marker, was expressed specifically in these cells, while *SOX2* is downregulated in both PGCLCs and somatic lineages (Figures 2B and 2E).

### Single-cell analysis of developing gonads

Before setting out to advance the development of PGCLCs, we collected information on *in vivo* developing germ cells as a reference. We thus prepared scRNA-seq libraries from developing marmoset gonads. Ovaries from embryonic day 74 (E74), E82, and newborn marmosets and testes from E74, E87, 22 day olds (22d), and 3-year- and 10-month-old marmosets were subjected to single-cell analyses (Table S1). After tSNE plotting, the germ cells of interest were extracted from each library (Figure S3). PGCs marked by *POU5F1* expression were extracted from fetal ovaries (E74 and E82) and testes (E87) (Figure S3). In E74 fetal testes, only a small number of PGCs were present, and they did not form a distinct cluster (Figure S3). Therefore, we did not extract the PGCs from this library. The body size of the E74 male was apparently smaller than that of its sibling E74 female. The small number of PGCs in E74 testes is, therefore, probably because many PGCs are still migrating and have not yet arrived at this stage (Aeckerle et al., 2015). In the somatic cells of E74 testes and ovaries, the expression of genes involved in initial sex differentiation was observed (*SRY*, *SOX9*, and *AMH* in testes, and *FOXL2* in ovaries) (Figure S5A), suggesting that sex differentiation is already initiated. Consistent with a previous report (Fereydouni et al., 2014), a wide range of cells (PGCs, oogonia, and early oocytes) were found in newborn ovaries (Figure S3). In 22d testes, germ cells were already differentiated into gonocytes, as shown by the decreased expression of *POU5F1* and elevated expression of *DDX4*. These expression patterns in 22d testis germ cells align with previously published studies (Albert et al., 2010; McKinnell et al., 2013; Mitchell et al., 2008).

### PGCLCs align with *in vivo* PGCs

**Figure 3:**
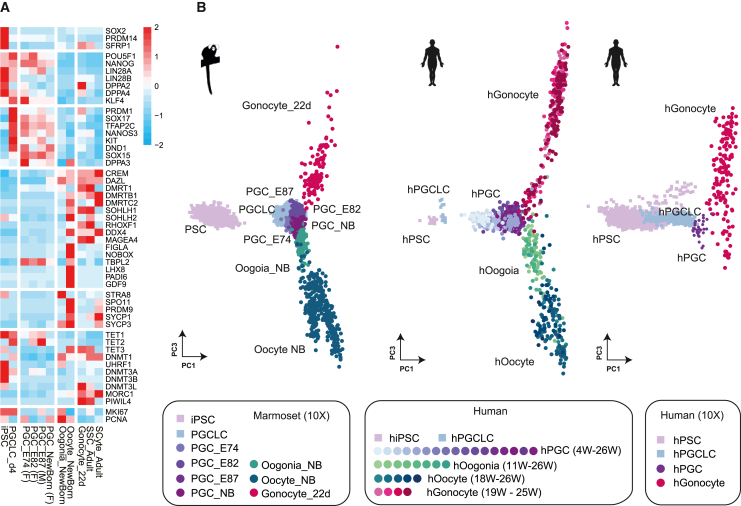
Gene expression analyses of PGCLCs and developmental germ cells (A) Heatmap analyses of marker genes. iPSCs, PGCLCs, and marmoset endogenous developmental germ cells were examined. For expression levels in these cell types, cells expressing iPSC or germ cell markers were extracted from each library (Figure S3). Relative expression levels were shown (n = 1). (B) PCA of PSCs, PGCLCs, and developing germ cells from and marmosets (left) and humans (center and right). Published human data were downloaded (Chen et al., 2019; Kojima et al., 2017; Li et al., 2017; Sohni et al., 2019) and analyzed together with marmoset data. Human data are shown in two separate panels according to the scRNA-seq platform. PSCs and germ cells were extracted from each 10x library based on marker gene expression. The anchoring function was used to integrate datasets of different platforms.

To examine whether our induced PGCLCs showed similar gene expression patterns to *in vivo* PGCs, we compared the expression of representative marker genes between *in vivo* germ cells and our PGCLCs (Figure 3A). A similar pattern was observed between *in vivo* PGCs and PGCLCs for the key marker genes. Both *in vivo* PGCs and PGCLCs showed the expression common marker genes for PSCs and PGCs (e.g., *POU5F1*, *NANOG*, *LIN28A*, *DPPA4*, and *KLF4*) and PGC marker genes (*PRDM1*, *SOX17*, *TFAP2C*, *NANOS3*, *KIT*, *DND1*, and *SOX15*). However, none of the PSC marker genes (e.g., *SOX2*), gonocyte/oogonium genes (e.g., *DAZL* and *SOHLH1*), oocyte genes (e.g., *FIGLA* and *NOBOX*), or meiotic genes (e.g., *STRA8* and *SPO11*) were expressed in both *in vivo* PGCs and PGCLCs. Some differences in gene expression levels were observed between *in vivo* PGCs and PGCLCs. For example, *TBPL2* was upregulated in both male and female *in vivo* PGCs compared with PGCLCs (Figure 3A). This difference may be, at least in part, explained by the difference in developmental stages between *in vivo* PGCs (late PGCs) and PGCLCs (early PGCs).

To obtain further evidence that our PGCLCs were indeed PGC-like, PCA was conducted using marmoset iPSCs, PGCLCs, and *in vivo* germ cells (PGCs, gonocytes, oogonia, and oocytes). As expected, marmoset PGCLCs clustered together with *in vivo* PGCs (Figure 3B, right panel). Furthermore, we added human data to this analysis (Chen et al., 2019; Kojima et al., 2017; Li et al., 2017; Sohni et al., 2019) to obtain additional supporting evidence. Human PSCs, PGCLCs, and germ cells from various developmental stages aligned well with their corresponding cells in marmosets (Figure 3B). These results suggest that human and marmoset early germ cell development is overall conserved and that our PGCLCs were indeed reminiscent of *in vivo* PGCs.

### Generation of xrtestes in mouse kidneys

**Figure 4:**
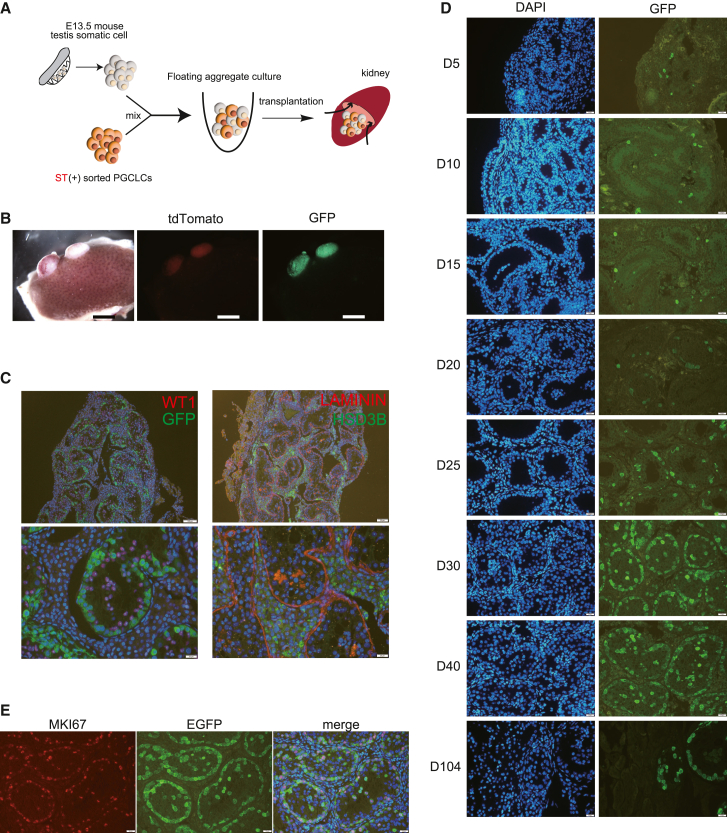
Proliferation of PGCLCs in xrtestis formed in mouse kidneys (A) Scheme for differentiation of PGCLCs. (B) d28_xrtestes formed under the kidney capsule. PGCLC (972-STCE)-derived cells express GFP and tdTomato. PGCLCs in xrtestes showed weaker tdTomato fluorescence than d4_PGCLCs. Scale bar, 2 mm. (C) Reconstitution of testis structure (d104_xrtestes). Immunohistochemical analyses of the transplanted tissues. Markers: *LAMININ* (basement membrane), *HSD3B* (Leydig cells), and *WT1* (Sertoli cells). PGCLC (971-STCE)-derived cells express GFP. Scale bars, 100 μm (top) and 20 μm (bottom). Testis structure was also confirmed for xrtestes using 972-STCE. (D) Time course change in the number of PGCLC (971-STCE)-derived cells during xrtestis development. Days after transplantation are indicated on the left side of the pictures. Scale bar, 20 μm. The same trend was observed in 972-STCE. (E) Many PGCLC (971-STCE)-derived cells express *MKI67* in d30_xrtestes. Scale bar, 20 μm. Similar observations were made for 972-STCE.

To advance the development of marmoset PGCLCs, we used the xrtestis system that has been used to differentiate human PGCLCs (Hwang et al., 2020). However, instead of the *in vitro* air-liquid interface culture used in humans (Hwang et al., 2020; Yamashiro et al., 2018), we employed mouse kidney transplantation, as this system develops mouse reconstituted testes well (Matoba and Ogura, 2011). To recapitulate the male *in vivo* developmental process, we used two male iPSC lines harboring the ST and CE transgenes (971-STCE and 972-STCE) (Figure S1). To prepare xrtestes, FACS-purified d4_PGCLCs were mixed with E13.5 testis somatic cells for floating aggregate culture. The next day, the aggregates were transplanted into kidney capsules (Figure 4A).

Ten days after transplantation (day 10), the transplanted aggregates formed a testicular cord-like structure (Figures 4B–4D). xrtestes were successfully formed in most cases (the results of transplantation experiments are listed in Table S2). This structure was maintained for over 100 days (Figures 4C and 4D) unless cancer developed. In the xrtestes, PGCLC-derived cells (EGFP- and tdTomato-positive) and Sertoli cells (*WT1*-positive) were found within the cord structure (marked by *LAMININ*). In contrast, cells expressing the Leydig cell marker *HSDB* were found in interstitial regions (Figure 4C). Only a small number of PGCLC-derived cells was observed in the xrtestes until day 30 after transplantation. However, by day 30, the number of PGCLC-derived cells dramatically increased, occupying the entire circumference of each tubule (Figure 4D). Consistent with this massive increase in cell number, *MKI67* signals were observed in many PGCLC-derived cells from day 30 xrtestes (d30_xrtestes) (Figure 4E). Thus, PGCLCs are incorporated into the tubules of the xrtestes, and these PGCLC-derived cells actively proliferate within the tubules.

### Differentiation of PGCLCs in xrtestes

**Figure 5:**
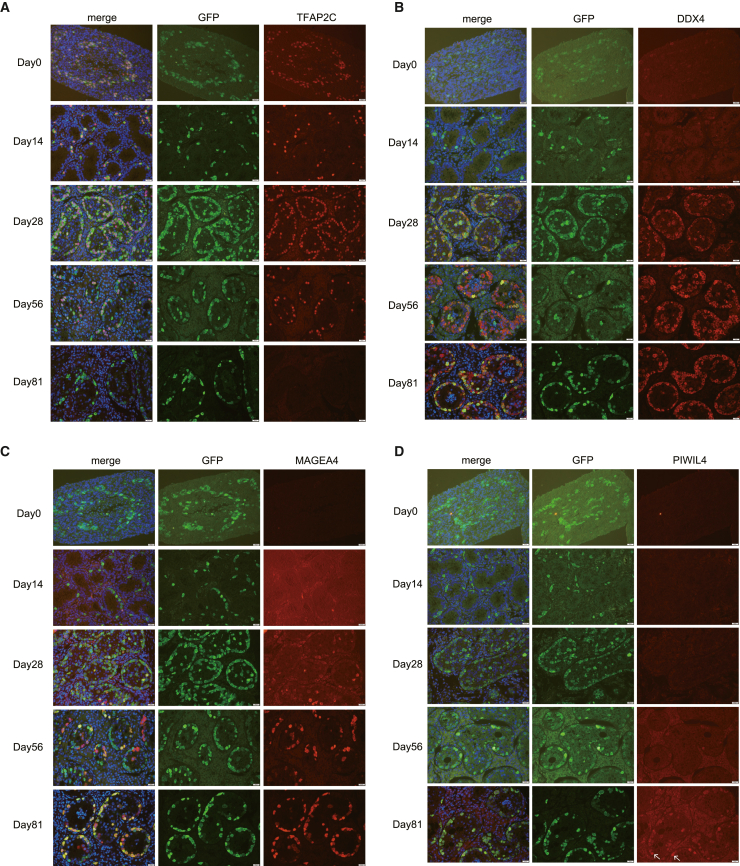
Differentiation of marmoset PGCLCs into gonocyte-like cells (A–D) Immunofluorescence analyses of marker gene expression in developing xrtestes (972-STCE) and d5_PGCLC aggregates. *TFAP2C* (A), *DDX4* (B), *MAGEA4* (C), and *PIWIL4* (D). White arrows indicate nuclear *PIWIL4* staining. The staining patterns of *PIWIL4* were different from those of cytoplasmic EGFP staining. Scale bar, 20 μm. The results of all xrtestis experiments are summarized in Table S2.

Human PGCLCs develop into gonocyte-like cells over ∼80 days (Hwang et al., 2020). We examined the expression of four well-characterized developmentally regulated marker genes *(TFAP2C*, *DDX4*, *MAGEA4*, and *PIWIL4*) in the xrtestes from several developmental points. Essentially similar results were obtained between the two different iPSC lines, 972-STCE and 971-STCE (Figures 5 and S6). *TFAP2C* (PGC marker) expression was observed in all PGCLC-derived cells before day 40 (Figures 5A and S6A). Subsequently, the number of PGCLC-derived cells expressing *TFAP2C* decreased dramatically. On day 81, none of the cells expressed this gene. No cells expressed gonocyte markers (*DDX4*, *MAGEA4*, and *PIWIL4*) on day 14. Almost all PGCLC-derived cells showed *DDX4* expression on day 28 (Figures 5B and S6A). On day 28, the expression of *MAGEA4* was also detected in only a small number of cells. As development progressed, the proportion of cells expressing *MAGEA4* increased, and all PGCLC-derived cells exhibited expression on days 81 and 104 (Figures 5C and S6A). *PIWIL4* was first, although weakly, observed in a small number of cells on days 81 and 104 (Figures 5D and S6A). Thus, these analyses revealed stepwise (in)activation of gonocyte (PGC) markers during xrtestis development, and the *in vivo* developmental pattern was recapitulated in our xrtestis system (Albert et al., 2010; Mitchell et al., 2008). qRT-PCR analyses also revealed the upregulation of these and some other gonocyte-expressed genes (*CREM*, *DMRT1*, *DMRT1B*, *DAZL*, *ZBTB16*, *FOXR1*, *RHOXF1*, *SOHLH1*, *SOHLH2*, and *RBM46*) in d56_, d81_, and d104_xrtestes (Figure S7). When d12_PGCLCs were used instead of d4_PGCLCs, *MAGEA4* expression was observed on day 43 after the transplantation (Figure S6B).

### Demethylation of PGCLCs in xenoreconstituted testes

**Figure 6:**
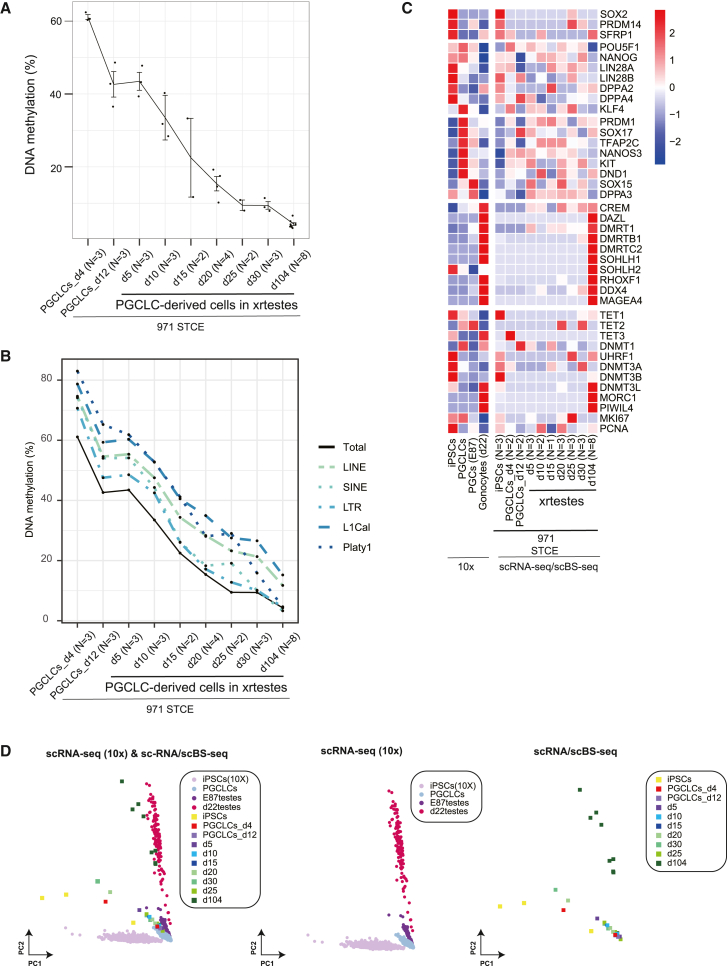
DNA methylation analyses during germ cell development from PGCLCs (A) Single-cell analysis of DNA methylation during PGCLC development. PGCLCs_d4 and PGCLCs_d12 correspond to 4 and 12 days of floating aggregate culture, respectively. d5–d104 represent duration (days) after transplantation into mouse kidneys. Each dot represents a single cell. Cells examined in each stage were derived from xrtestes grown in one host mouse. (B) Developmental dynamics of DNA methylation levels of retrotransposons. The average values are shown. (C) Heatmap analyses of marker genes. Relative expression levels were shown. Apparently variable expression of pluripotent genes during xrtestis development is likely, at least in part, due to technical issues (small number of single cells, medium expression levels, and multiple stages analyzed. (D) The data obtained by 10x and scRNA-seq/scBS-seq are shown together (left) or separately (center and right). The anchoring function was not used to integrate datasets of different platforms. There is a small displacement between the iPSCs of the 10x platform and iPSCs of the scRNA-seq/scBS-seq platform.

PGC development is accompanied by progressive loss of DNA methylation (Shirane et al., 2016). To determine DNA methylation status, we conducted single-cell bisulfite sequencing (scBS-seq) analyses in PGCLCs and PGCLC-derived cells in the xrtestes (Table S1 and S3). Simultaneously, RNA expression was analyzed in the same single cells using the cytoplasmic fraction. In d4_PGCLCs, the average DNA methylation level was 61.1% (Figure 6A). Interestingly, the level decreased to 45.7% in d12_PGCLCs, suggesting the occurrence of demethylation during long floating aggregate culture. The xrtestes were generated using d4_PGCLCs. DNA methylation levels decreased gradually in xrtestes (Figure 6A). Although we still detected some residual methylation in d30_xrtestes (9.4%), this level is close to the minimum level in d104_xrtestes (4.3%). Thus, DNA demethylation was recapitulated in our xrtestis system. However, the establishment of DNA methylation was likely not initiated, even in d104_xrtestes.

DNA methylation plays a critical role in the repression of retrotransposons in germline cells. In both mice and humans, active and young retrotransposons (e.g., IAP and LINE1 in mice, Alu and LINE1 in humans) show relatively high levels of residual DNA methylation in demethylated PGC genomes (Guo et al., 2015; Kobayashi et al., 2013; Seisenberger et al., 2012). Two types of potentially active retrotransposons exist in the marmoset genome. One is the LINE1 element, and the other is a very short ∼100-bp SINE element named Platy-1 (Konkel et al., 2016). The DNA methylation dynamics of these two active retrotransposons (LINE1 and Platy-1) and three major classes of retrotransposons sequences (LINE, LTR, and SINE) were examined. In d4_PGCLCs, all three major retrotransposon sequences (LINE, 76.6%; LTR, 70.6%; SINE, 74.1%) showed higher levels than the genome average (61.1%), and the two active retrotransposons (LINE1: 78.7%, Platy-1: 82.9%) showed the highest levels (Figure 6B). As PGCLC development progressed, all retrotransposons lost DNA methylation with dynamics similar to those of the genomic average. In d104_xrtestes, DNA methylation levels of all the examined retrotransposons were decreased more than 5-fold (LINE1, 15.2% from 76.6%) to 17-fold (Platy-1, 4.7% from 82.9%). LINE1 (15.2%) and LINE (11.8%) still showed much higher levels than the genomic average (4.3%). On the other hands, other retrotransposons (LTR, 4.5%; SINE, 3.3%; Platy-1, 4.7%) showed similar levels to the genomic average (Figure 6B). Thus, a higher level of residual methylation was retained in LINE1 but not in Platy-1.

### Comparison of germ cell development *in vivo* and in xrtestes

**Figure 7:**
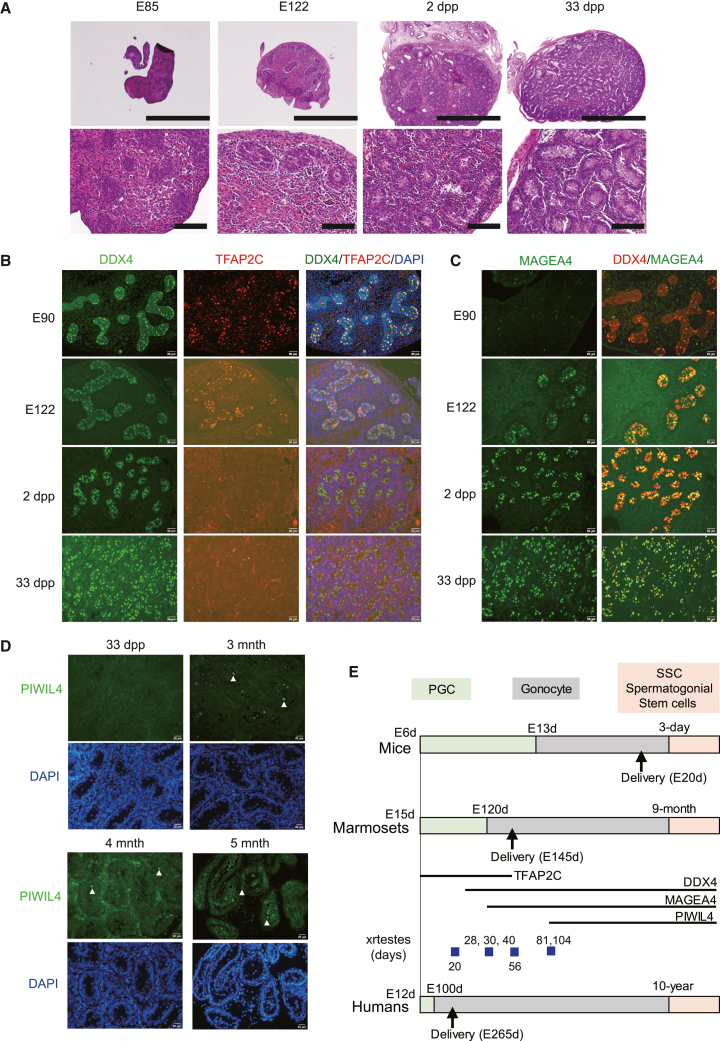
*In vivo* germ cell development in marmoset testes (A) Histology of developing testes. Scale bars, 2 mm (top) and 200 μm (bottom). n = 1. (B–D) Immunohistochemical analyses of the developing testes. Germ cell markers: *DDX4/TFAP2C* (B), *DDX4/MAGEA4* (C), and *PIWIL4* (D). Arrow heads in (D) indicate *PIWIL4*-positive cells. Scale bars, 20 μm. n = 1. (E) Schematic diagram for germ cell development in mice, marmosets, and humans. The expression windows of *TFAP2C*, *DDX4*, *MAGEA4*, and *PIWIL4* in marmosets are shown. PGC to gonocyte transition was determined based on the disappearance of *TFAP2C* expression and the start of *MAGEA4* expression. Possible corresponding stages of xrtestis development are shown.

To correlate germ cell development in xrtestes with *in vivo* germ cell development, we analyzed the RNA expression of PGCLC-derived germ cells, in which we analyzed DNA methylation. Upon differentiation of iPSCs into PGCLCs, the expression of *UHRF1* (involved in DNA methylation maintenance) and *de novo* DNA methyltransferase *DNMT3A/3B/3L* decreased (Figure 6C). This decrease may be involved in the demethylation of the PGCLC genome. At the initial stage of xrtestis development (d5−d30), pluripotent genes (*POU5F1*, *NANOG*, *LIN28A*, and *KLF4*) and PGC genes (*PRDM1*, *SOX17*, *TFAP2C*, *NANOS3*, *KIT*, *DND1*, *SOX15*, and *DPPA3*) were expressed. Their expression was decreased in d104_xrtestes. Instead, *DNMT3L*, *PIWIL4*, and *MORC1* were highly upregulated, establishing the stage for *de novo* methylation of retrotransposons. In addition, the expression of gonocyte genes (*CREM*, *DAZL*, *DMRT1*, *DMRTB1*, *DMRTC2*, *SOHLH1*, *SOHLH2*, *RHOXF1*, *DDX4*, and *MAGEA4*) was observed in d104_xrtestes as well as in gonocytes from *in vivo* 22d testes (Figure 6C). Some of them (*CREM*, *DMRT1*, and *DDX4*) were expressed from earlier stages of xrtestis development (Figures 6C and 5B for *DDX4*). These genes were expressed, although very weakly, in *in vivo* PGCs from E87 testes (Figure 6C) or E90 testes (see Figure 7 for *DDX4*), suggesting that developmentally regulated gene expression is recapitulated in the xrtestis system. PCA revealed that PGCLC-derived cells, except those from d104_xrtestes, closely aligned with E87 testis germ cells (Figure 6D). On the other hand, PGCLC-derived germ cells from d104_xrtestes clustered together with 22d testis germ cells. Thus, PGCLCs differentiate into gonocyte-like cells in the xrtestis.

To more precisely correlate PGCLC-derived cell development with *in vivo* germ cell development, immunohistochemichal analyses (TFAP2C, DDX4, MAGEA4, and PIWIL4) were conducted using testes from several developmental stages (E85, E90, E122, 2-day, 33-day, 3-month, 4-month, and 5-month testes) (Figure 7). In E85 and E90 testes, many germ cells co-expressed *TFAP2C* and *DDX4*, although a small number of cells expressed only *TFAP2C* (Figure 7B and data not shown). In E122 testes, many germ cells still co-expressed *TFAP2C* and *DDX4*, and *MAGEA4* expression was started in a small part of germ cells (similar to day 28, 30, and 40 xrtestes) (Figure 7C). In 2-day testes, many germ cells expressed *MAGEA4*, which is similar to day 56 xrtestes. Almost all (none) of germ cells express *MAGEA4* (*TFAP2C*) in 33-day testes. *PIWIL4* expression, which was weakly observed in day 81 and 104 xrtestes, was first observable in 3-month testes (Figures 5D, 7D, 7E, and S6A).

## Discussion

In this study, marmoset PGCLCs were generated from iPSCs using an mRNA-transfection-based method. This is the first report of PGCLC generation using mRNAs. Since this method is simple and efficient, it may also be useful for other species. Furthermore, the generated PGCLCs differentiated into gonocyte-like cells in the xrtestes that were transplanted under the kidney capsules of immunodeficient mice. Stepwise expression of PGC and gonocyte marker genes was observed. DNA methylation was progressively lost and almost completely erased in the gonocyte-like cells. Thus, early germ cell development *in vivo* was recapitulated by our PGCLC-initiated system. This study provides a platform for developmental studies on marmoset germ cells and the generation of genetically modified marmosets.

We induced PGCLCs from iPSCs using a combination of *SOX17* mRNA transfection and subsequent floating aggregate culture. Our method was based on a report on PGCLC generation by *SOX17* overexpression using an inducible system (Irie et al., 2015; Kobayashi et al., 2017), which requires prior transgene integration. To omit this step, we used mRNA transfection-based overexpression. Although the induction rate was highly dependent on the iPSC lines (data not shown), as in humans (Chen et al., 2017), our induction efficiency usually reached >80% when highly competent lines were used (Figures 1B and S1). Since this efficiency is comparable with or higher than those of existing methods (Irie et al., 2015; Jo et al., 2022; Kobayashi et al., 2017; Sakai et al., 2020; Sasaki et al., 2015; Sosa et al., 2018; Yoshimatsu et al., 2021), we believe that the mRNA transfection method reported here serves as an alternative method.

After developing a solid foundation for PGCLC induction, we aimed to differentiate PGCLCs into a more advanced state. Matoba and Ogura (2011) reported that *in vivo* mouse PGCs developed into spermatids in reconstituted testes transplanted under the kidney capsule. This led us to examine whether immunodeficient mouse kidneys serve as suitable sites to develop xrtestes. The xrtestes developed well under the kidney capsule. Using this technique, PGCLCs in the xrtestes were found to differentiate into gonocyte-like cells. All PGCLC-derived cells from the d81_xrtestes were negative for the PGC marker *TFAP2C* (Figure 5). Given that a small number of the germ cells still express *TFAP2C* in newborn testes (Figure 7B), gonocyte-like cells in d81_xrtestes are more developmentally advanced than newborn testis germ cells. However, our bisulfite sequencing analyses showed that PGCLC-derived cells in d81_ and d104_xrtestes did not undergo *de novo* DNA methylation (Figure 6). In marmoset testes, *de novo* DNA methylation is initiated at 4 months at the latest (Langenstroth-Rower et al., 2017), although the precise timing has not yet been determined. Therefore, gonocyte-like cells in d81_ and d104_xrtestes likely correspond to *in vivo* gonocytes between newborn and 4-month-old animals. *PIWIL4* expression was first observable in 3-month testes, but not in 2-month testes (Figures 7D and 7E). In d81_ and d104_xrtestes, weak *PIWIL4* expression was observed. Thus, these xrtestes likely correspond to 2- or 3-month testes *in vivo*. The kidney transplantation method reported here will be a robust *in vivo* method for male PGCLC differentiation in other species as well. Reconstituted embryonic ovaries from mice and cynomolgus monkeys develop well in mouse kidneys (Matoba and Ogura, 2011; Mizuta et al., 2022). Therefore, the kidney transplantation of (xeno)reconstituted ovaries may be also useful for advancement of female PGCLC development in marmosets and other species.

Successful marmoset PGCLC induction has been reported previously by Yoshimatsu et al. (2021). However, their induction efficiency (∼40% for the two ESC lines and 1%–2% for the two iPSC lines) seemed to be not as high as ours. In addition, their study did not test the developmental potential of these PGCLCs. By contrast, PGCLCs were differentiated into gonocyte-like cells in our study. Their methods involved both transgene (*SOX17* and *BLIMP1*) overexpression and pre-ME/iMeLC induction steps, and it took 10 days (+prior transgene integration) for the procedure. Our method required only 6 days, and no prior transgene integration is required. Furthermore, we did not use *BLIMP1*, because the addition of *BLIMP1* mRNAs to *SOX17* mRNAs did not have any positive effect on PGCLC induction in our system (Figure S2C). Combinatorial expression of *BLIMP1* and *SOX17* has been reported to promote PGCLCs in humans (Kobayashi et al., 2017). Species difference or different methods used likely account for the discrepancy of the effect of *BLIMP1*. Recently, another group reported marmoset PGCLC induction from PSCs (Seita et al., 2022). Their induction efficiency was 40% at the highest using a similar method reported in cynomolgus monkeys and rabbits (Kobayashi et al., 2021; Sakai et al., 2020). They cultured PSCs in the presence of the WNT inhibitor IWR-1, and PGCLCs were directly induced from PSCs without undergoing pre-ME/iMeLC. Although their PGCLCs differentiated into *DDX4*-positive cells (corresponding to late PGCs or gonocytes), complete DNA demethylation and the potential for differentiation into *MAGEA4*-positive gonocyte-like cells were not examined. Thus, our study provides two efficient and useful systems associated with marmoset PGCLCs: (1) an mRNA-transfection-based PGCLC induction system and (2) a kidney transplantation-based PGCLC to gonocyte-like cell differentiation system.

In contrast to the PSCs cultured in the presence of the WNT inhibitor IWR-1 (Seita et al., 2022), our iPSCs cultured in MEF-conditioned medium did not directly differentiate into PGC lineage upon aggregate formation. This difference in germline competency may be due to differences in culture conditions and/or cell lines. We speculate that *SOX17* mRNA overexpression makes iPSC transcriptome closer to PGC, and these iPSCs become preferentially differentiating into PGC lineage upon aggregate formation. Given that mRNA transfection only can induce PGCLCs (Figure 1B), cytokines are not essential for PGCLC induction in our system. However, omitting growth factors slightly reduced the number of PGCLCs induced. At least, SCF is required for the maintenance of PGCLCs. Further studies are needed to determine other cytokines used here play any positive roles in the *SOX17* mRNA-mediated induction of PGCLCs.

Long-term *SOX17* overexpression in human ESCs have been reported to give competency for differentiation into DE lineage (Seguin et al., 2008). In contrast, our short-term overexpression in marmoset iPSCs and aggregate formation resulted in exclusive differentiation into PGC lineage (Figures 2D and 2E). What is the reason for this difference? One possibility is the duration of *SOX17* overexpression. Consistent with this, we observed decreased efficiency of PGCLC induction by increasing the mRNA transfection period (Figure S2A). In addition, aggregate culture conditions may promote differentiation into PGC lineage. Another possibility is species-specific difference. Marmoset primed PSCs are suggested to be slightly earlier developmental stage than human primed PSCs (Bergmann et al., 2022). Interestingly, a recent study suggested that *SOX17* binding to PGC and DE promoter/enhancer was determined by the cofactors (PGC cofactors: *POU5F1*, *NANOG*, and *TFAP2C*; DE cofactors: *EOMES*, *SMAD2/3/4*, *FOXA1/A2*, and *ZIC2/3/5*) (Tang et al., 2022). The earlier state of marmoset PSCs, therefore, might promote directing *SOX17* to PGC promoter/enhancer in preference to DE promoter/enhancer.

For the generation of functional gametes, the gonocyte-like cells generated in this study require further development. The next step is further differentiation into late gonocyte-like cells that undergo *de novo* DNA methylation. It is important to understand the cues that initiate *de novo* DNA methylation. Furthermore, the current protocol requires a long time to differentiate gonocyte-like cells from PGCLCs. Shortening this time is also an important next step. However, undertaking the normal demethylation process in PGCs is likely important for generating functional gametes. In fact, bypassing this resulted in abnormal DNA methylation patterns in mouse oocytes (Hamazaki et al., 2021). Furthermore, *in vitro* PGCLC culture and the resultant prior erasure of DNA methylation have been reported to be essential for the spermatogenic potential of PGCLC-derived spermatogonial stem cells (Ishikura et al., 2021). DNA methylation dynamics revealed in this study are, in part, useful for determining which developmental stage can be bypassed without affecting the DNA demethylation process. Our study provides a solid foundation for complete generation of gametes from pluripotent stem cells.

## Experimental procedures

### Resource availability

#### Corresponding author

Further information should be requested from the corresponding author, Toshiaki Watanabe (watanabe-tos@ncchd.go.jp).

#### Materials availability

Materials can be requested from the corresponding author.

### Cell culture

Marmoset iPSCs were cultured in MEF-conditioned primate ES cell medium (REPROCELL, RCHEMD001) containing bFGF (REPROCELL, RCHEOT003) and 1× antibiotic-antimycotic (Nacalai Tesque, 0289-54) (Watanabe et al., 2019). The methods for the induction of PGCLCs are found in the supplemental information.

### Single-cell library generation and data analyses

The sample information is summarized in Table S1. Summary statistics for simultaneous scRNA-seq and scBS-seq analyses are found in Table S3. See supplemental information for the library generation and the analyses.

## Data Availability

Single-cell data generated by 10x Genomics platform were deposited to ArrayExpress (accession no. E-MTAB-12123) and scRNA-seq/scBS-seq data were registered to DDBJ (accession nos. DRA014666 and DRA014672). Data and code will be shared with the research community upon request.

## Supplemental information

Document S1. Figures S1–S7, Tables S1–S4, and experimental procedures

Document S2. Article plus supplemental information

## References

[bib1] Aeckerle N., Drummer C., Debowski K., Viebahn C., Behr R. (2015). Primordial germ cell development in the marmoset monkey as revealed by pluripotency factor expression: suggestion of a novel model of embryonic germ cell translocation. Mol. Hum. Reprod..

[bib2] Albert S., Ehmcke J., Wistuba J., Eildermann K., Behr R., Schlatt S., Gromoll J. (2010). Germ cell dynamics in the testis of the postnatal common marmoset monkey (Callithrix jacchus). Reproduction.

[bib3] Bergmann S., Penfold C.A., Slatery E., Siriwardena D., Drummer C., Clark S., Strawbridge S.E., Kishimoto K., Vickers A., Tewary M. (2022). Spatial profiling of early primate gastrulation in utero. Nature.

[bib4] Castillo-Venzor A., Penfold C.A., Morgan M.D., Tang W.W.C., Kobayashi T., Wong F.C.K., Bergmann S., Slatery E., Boroviak T.E., Marioni J.C., Surani M.A. (2022). Origin and segregation of the human germline. bioRxiv.

[bib5] Chen D., Liu W., Lukianchikov A., Hancock G.V., Zimmerman J., Lowe M.G., Kim R., Galic Z., Irie N., Surani M.A. (2017). Germline competency of human embryonic stem cells depends on eomesodermin. Biol. Reprod..

[bib6] Chen D., Sun N., Hou L., Kim R., Faith J., Aslanyan M., Tao Y., Zheng Y., Fu J., Liu W. (2019). Human primordial germ cells are specified from lineage-primed progenitors. Cell Rep..

[bib7] Fereydouni B., Drummer C., Aeckerle N., Schlatt S., Behr R. (2014). The neonatal marmoset monkey ovary is very primitive exhibiting many oogonia. Reproduction.

[bib8] Guo F., Yan L., Guo H., Li L., Hu B., Zhao Y., Yong J., Hu Y., Wang X., Wei Y. (2015). The transcriptome and DNA methylome landscapes of human primordial germ cells. Cell.

[bib9] Hamazaki N., Kyogoku H., Araki H., Miura F., Horikawa C., Hamada N., Shimamoto S., Hikabe O., Nakashima K., Kitajima T.S. (2021). Reconstitution of the oocyte transcriptional network with transcription factors. Nature.

[bib10] Hong H., Takahashi K., Ichisaka T., Aoi T., Kanagawa O., Nakagawa M., Okita K., Yamanaka S. (2009). Suppression of induced pluripotent stem cell generation by the p53-p21 pathway. Nature.

[bib11] Hwang Y.S., Suzuki S., Seita Y., Ito J., Sakata Y., Aso H., Sato K., Hermann B.P., Sasaki K. (2020). Reconstitution of prospermatogonial specification in vitro from human induced pluripotent stem cells. Nat. Commun..

[bib12] Irie N., Weinberger L., Tang W.W.C., Kobayashi T., Viukov S., Manor Y.S., Dietmann S., Hanna J.H., Surani M.A. (2015). SOX17 is a critical specifier of human primordial germ cell fate. Cell.

[bib13] Ishikura Y., Ohta H., Sato T., Murase Y., Yabuta Y., Kojima Y., Yamashiro C., Nakamura T., Yamamoto T., Ogawa T., Saitou M. (2021). In vitro reconstitution of the whole male germ-cell development from mouse pluripotent stem cells. Cell Stem Cell.

[bib14] Jo K., Teague S., Chen B., Khan H.A., Freeburne E., Li H., Li B., Ran R., Spence J.R., Heemskerk I. (2022). Efficient differentiation of human primordial germ cells through geometric control reveals a key role for Nodal signaling. Elife.

[bib15] Kobayashi H., Sakurai T., Miura F., Imai M., Mochiduki K., Yanagisawa E., Sakashita A., Wakai T., Suzuki Y., Ito T. (2013). High-resolution DNA methylome analysis of primordial germ cells identifies gender-specific reprogramming in mice. Genome Res..

[bib16] Kobayashi T., Castillo-Venzor A., Penfold C.A., Morgan M., Mizuno N., Tang W.W.C., Osada Y., Hirao M., Yoshida F., Sato H. (2021). Tracing the emergence of primordial germ cells from bilaminar disc rabbit embryos and pluripotent stem cells. Cell Rep..

[bib17] Kobayashi T., Zhang H., Tang W.W.C., Irie N., Withey S., Klisch D., Sybirna A., Dietmann S., Contreras D.A., Webb R. (2017). Principles of early human development and germ cell program from conserved model systems. Nature.

[bib18] Kojima Y., Sasaki K., Yokobayashi S., Sakai Y., Nakamura T., Yabuta Y., Nakaki F., Nagaoka S., Woltjen K., Hotta A. (2017). Evolutionarily distinctive transcriptional and signaling programs drive human germ cell lineage specification from pluripotent stem cells. Cell Stem Cell.

[bib19] Konkel M.K., Ullmer B., Arceneaux E.L., Sanampudi S., Brantley S.A., Hubley R., Smit A.F.A., Batzer M.A. (2016). Discovery of a new repeat family in the Callithrix jacchus genome. Genome Res..

[bib20] Langenstroth-Rower D., Gromoll J., Wistuba J., Trondle I., Laurentino S., Schlatt S., Neuhaus N. (2017). De novo methylation in male germ cells of the common marmoset monkey occurs during postnatal development and is maintained in vitro. Epigenetics.

[bib21] Li L., Dong J., Yan L., Yong J., Liu X., Hu Y., Fan X., Wu X., Guo H., Wang X. (2017). Single-cell RNA-seq analysis maps development of human germline cells and gonadal niche interactions. Cell Stem Cell.

[bib22] Matoba S., Ogura A. (2011). Generation of functional oocytes and spermatids from fetal primordial germ cells after ectopic transplantation in adult mice. Biol. Reprod..

[bib23] McKinnell C., Mitchell R.T., Morris K., Anderson R.A., Kelnar C.J.H., Wallace W.H., Sharpe R.M. (2013). Perinatal germ cell development and differentiation in the male marmoset (Callithrix jacchus): similarities with the human and differences from the rat. Hum. Reprod..

[bib24] Mitchell R.T., Cowan G., Morris K.D., Anderson R.A., Fraser H.M., McKenzie K.J., Wallace W.H.B., Kelnar C.J.H., Saunders P.T.K., Sharpe R.M. (2008). Germ cell differentiation in the marmoset (Callithrix jacchus) during fetal and neonatal life closely parallels that in the human. Hum. Reprod..

[bib25] Mizuta K., Katou Y., Nakakita B., Kishine A., Nosaka Y., Saito S., Iwatani C., Tsuchiya H., Kawamoto I., Nakaya M. (2022). Ex vivo reconstitution of fetal oocyte development in humans and cynomolgus monkeys. EMBO J..

[bib26] Park J.E., Sasaki E. (2020). Assisted reproductive techniques and genetic manipulation in the common marmoset. ILAR J..

[bib27] Phillips I.R. (1976). The embryology of the common marmoset (Callithrix jacchus). Adv. Anat. Embryol. Cell Biol..

[bib28] Poleganov M.A., Eminli S., Beissert T., Herz S., Moon J.I., Goldmann J., Beyer A., Heck R., Burkhart I., Barea Roldan D. (2015). Efficient reprogramming of human fibroblasts and blood-derived endothelial progenitor cells using nonmodified RNA for reprogramming and immune evasion. Hum. Gene Ther..

[bib29] Sakai Y., Nakamura T., Okamoto I., Gyobu-Motani S., Ohta H., Yabuta Y., Tsukiyama T., Iwatani C., Tsuchiya H., Ema M. (2020). Induction of the germ cell fate from pluripotent stem cells in cynomolgus monkeysdagger. Biol. Reprod..

[bib30] Sasaki K., Yokobayashi S., Nakamura T., Okamoto I., Yabuta Y., Kurimoto K., Ohta H., Moritoki Y., Iwatani C., Tsuchiya H. (2015). Robust in vitro induction of human germ cell fate from pluripotent stem cells. Cell Stem Cell.

[bib31] Seguin C.A., Draper J.S., Nagy A., Rossant J. (2008). Establishment of endoderm progenitors by SOX transcription factor expression in human embryonic stem cells. Cell Stem Cell.

[bib32] Seisenberger S., Andrews S., Krueger F., Arand J., Walter J., Santos F., Popp C., Thienpont B., Dean W., Reik W. (2012). The dynamics of genome-wide DNA methylation reprogramming in mouse primordial germ cells. Mol. Cell.

[bib33] Seita Y., Cheng K., McCarrey J.R., Yadu N., Cheeseman I., Bagwell A., Ross C.N., Santana-Toro I., Yen L.-H., Vargas S. (2022). Efficient generation of marmoset primordial germ cell-like cells using induced pluripotent stem cells. bioRxiv.

[bib34] Shirane K., Kurimoto K., Yabuta Y., Yamaji M., Satoh J., Ito S., Watanabe A., Hayashi K., Saitou M., Sasaki H. (2016). Global landscape and regulatory principles of DNA methylation reprogramming for germ cell specification by mouse pluripotent stem cells. Dev. Cell.

[bib35] Sohni A., Tan K., Song H.W., Burow D., de Rooij D.G., Laurent L., Hsieh T.C., Rabah R., Hammoud S.S., Vicini E., Wilkinson M.F. (2019). The neonatal and adult human testis defined at the single-cell level. Cell Rep..

[bib36] Sosa E., Chen D., Rojas E.J., Hennebold J.D., Peters K.A., Wu Z., Lam T.N., Mitchell J.M., Sukhwani M., Tailor R.C. (2018). Differentiation of primate primordial germ cell-like cells following transplantation into the adult gonadal niche. Nat. Commun..

[bib37] Tang W.W.C., Castillo-Venzor A., Gruhn W.H., Kobayashi T., Penfold C.A., Morgan M.D., Sun D., Irie N., Surani M.A. (2022). Sequential enhancer state remodelling defines human germline competence and specification. Nat. Cell Biol..

[bib38] Tyser R.C.V., Mahammadov E., Nakanoh S., Vallier L., Scialdone A., Srinivas S. (2021). Single-cell transcriptomic characterization of a gastrulating human embryo. Nature.

[bib39] Watanabe T., Yamazaki S., Yoneda N., Shinohara H., Tomioka I., Higuchi Y., Yagoto M., Ema M., Suemizu H., Kawai K., Sasaki E. (2019). Highly efficient induction of primate iPS cells by combining RNA transfection and chemical compounds. Gene Cell..

[bib40] Yamashiro C., Sasaki K., Yabuta Y., Kojima Y., Nakamura T., Okamoto I., Yokobayashi S., Murase Y., Ishikura Y., Shirane K. (2018). Generation of human oogonia from induced pluripotent stem cells in vitro. Science.

[bib41] Yoshimatsu S., Nakajima M., Iguchi A., Sanosaka T., Sato T., Nakamura M., Nakajima R., Arai E., Ishikawa M., Imaizumi K. (2021). Non-viral induction of transgene-free iPSCs from somatic fibroblasts of multiple mammalian species. Stem Cell Rep..

