# Supplementary material for: mRNA-based generation of marmoset PGCLCs capable of differentiation into gonocyte-like cells
Source: Stem Cell Reports. 2023 Sep 7;18(10):1987–2002. doi: 10.1016/j.stemcr.2023.08.006 (PMC10656353; doi:10.1016/j.stemcr.2023.08.006)
Supplement: Document S1. Figures S1–S7, Tables S1–S4, and experimental procedures [file mmc1.pdf]

Stem Cell Reports, Volume *18*

Supplemental Information

mRNA-based generation of marmoset PGCLCs capable of differentiation into gonocyte-like cells

Musashi Kubiura-Ichimaru, Christopher Penfold, Kazuaki Kojima, Constance Dollet, Haruka Yabukami, Katsunori Semi, Yasuhiro Takashima, Thorsten Boroviak, Hideya Kawaji, Knut Woltjen, Aki Minoda, Erika Sasaki, and Toshiaki Watanabe

Figure S1

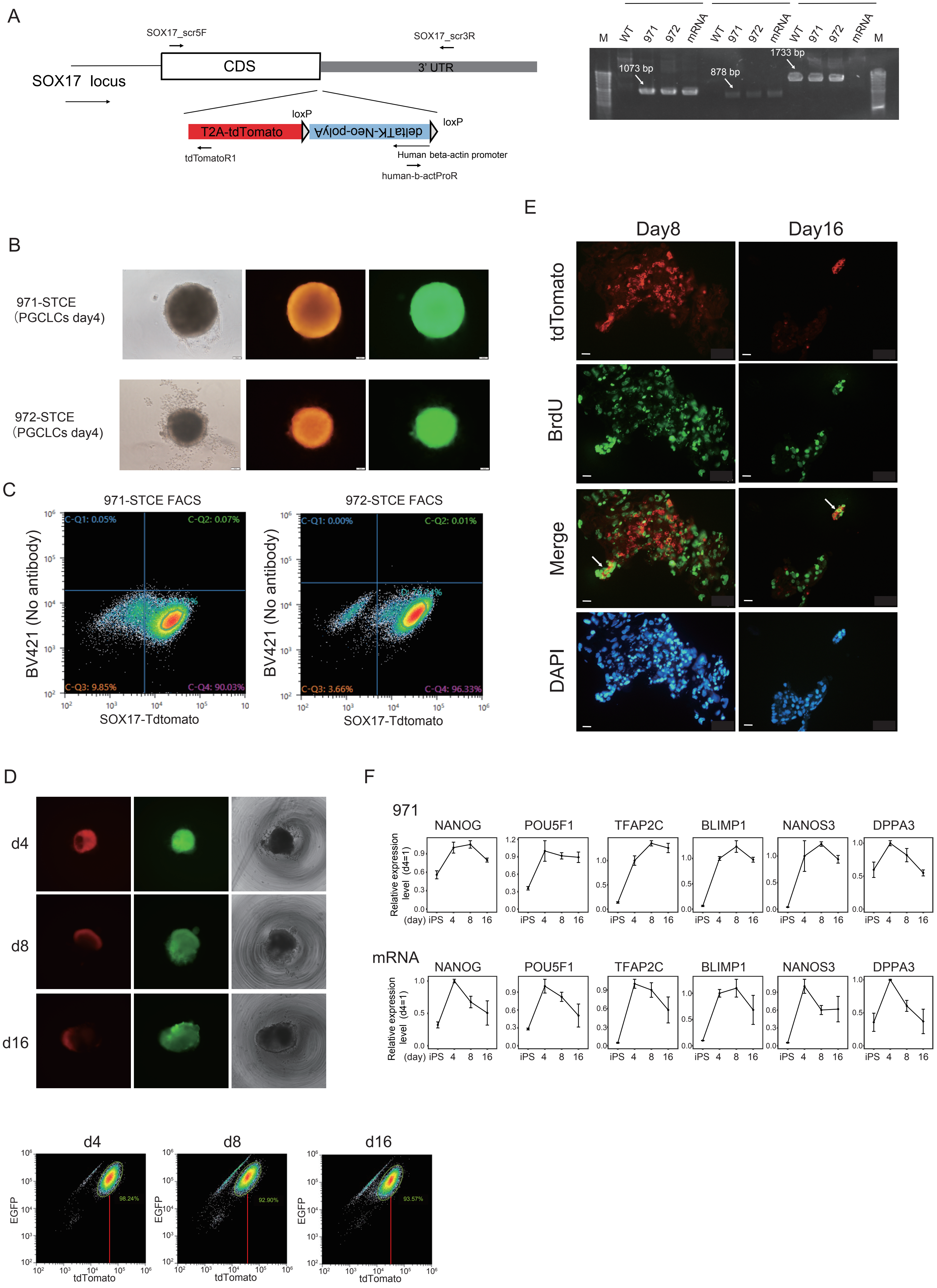

A
SOX17_scr5F
SOX17_scr3R
CDS
3' UTR
SOX17 locus
loxP
T2A-tdTomato
deltaTK-Neo-polyA
loxP
tdTomatoR1
Human beta-actin promoter
human-b-actProR
SOX17_scr5F tdTomatoR1
human-b-actProR SOX17_scr3R
SOX17_scr5F SOX17_scr3R
M WT 971 972 mRNA WT 971 972 mRNA WT 971 972 mRNA M
1073 bp
878 bp
1733 bp
B
971-STCE (PGCLCs day4)
972-STCE (PGCLCs day4)
C
971-STCE FACS
972-STCE FACS
BV421 (No antibody)
SOX17-Tdtomato
C-Q1: 0.05%
C-Q2: 0.07%
C-Q3: 9.85%
C-Q4: 90.03%
C-Q1: 0.00%
C-Q2: 0.01%
C-Q3: 3.66%
C-Q4: 96.33%
E
Day8
Day16
tdTomato
BrdU
Merge
DAPI
D
d4
d8
d16
EGFP
tdTomato
98.24%
92.90%
93.57%
F
971
mRNA
NANOG
POU5F1
TFAP2C
BLIMP1
NANOS3
DPPA3
Relative expression level (d4=1)
(day) iPS 4 8 16

Figure S2

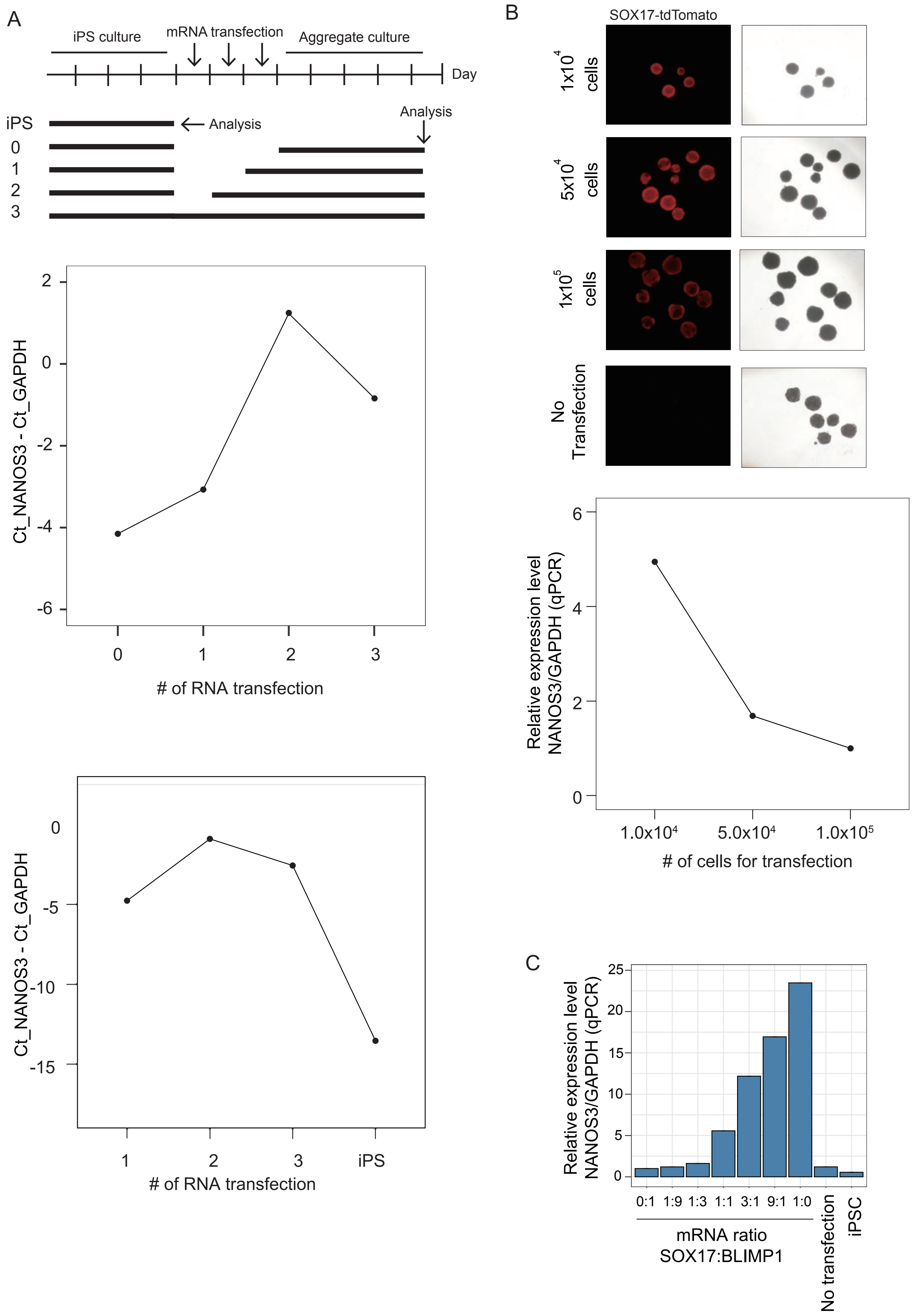
A
iPS culture
mRNA transfection
Aggregate culture
Day
iPS
Analysis
Analysis
0
1
2
3
Ct_NANOS3 - Ct_GAPDH
# of RNA transfection
iPS
B
SOX17-tdTomato
1x10^4 cells
5x10^4 cells
1x10^5 cells
No Transfection
Relative expression level NANOS3/GAPDH (qPCR)
1.0x10^4
5.0x10^4
1.0x10^5
# of cells for transfection
C
0:1
1:9
1:3
1:1
3:1
9:1
1:0
mRNA ratio
SOX17:BLIMP1
No transfection
iPSC

Figure S3

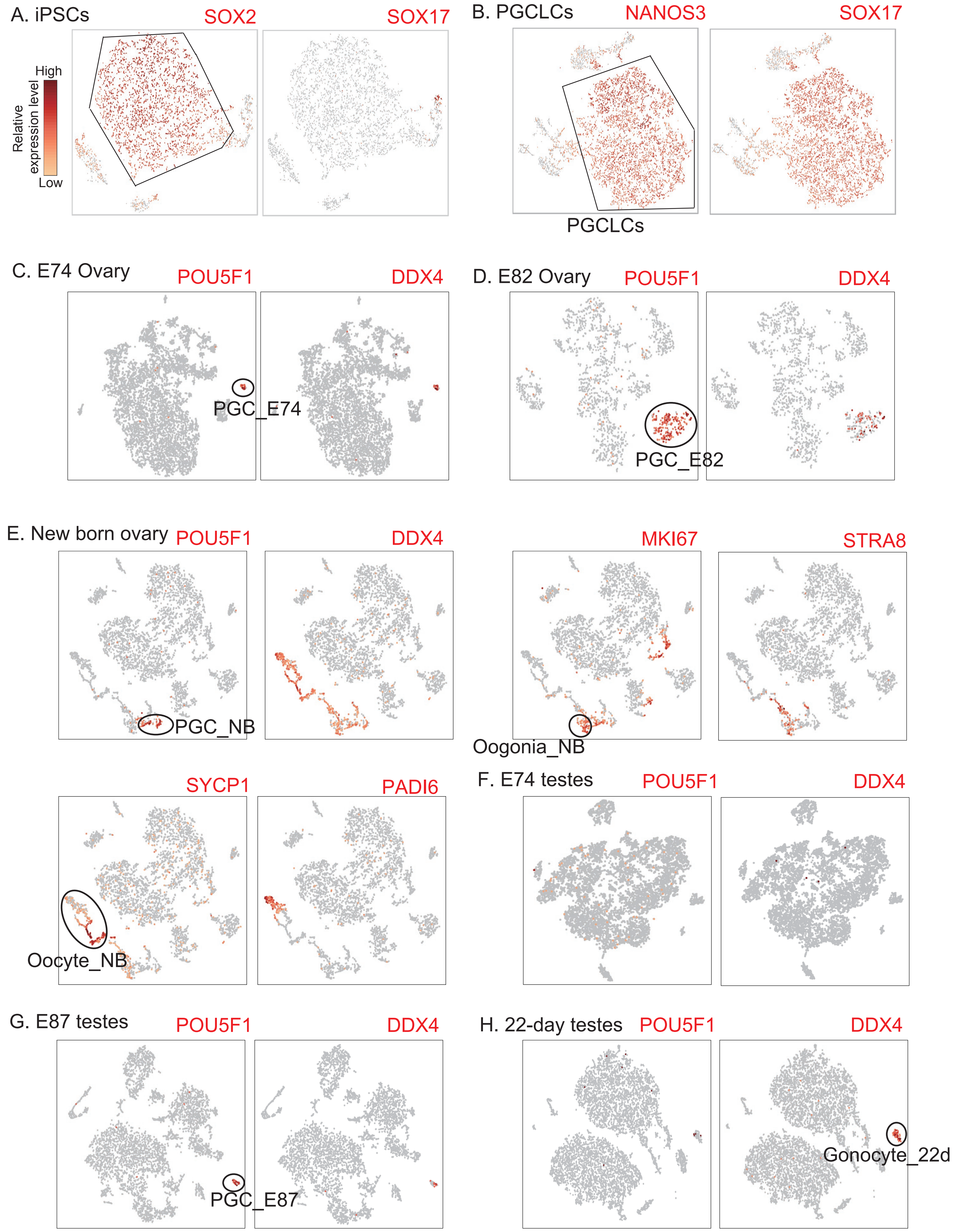
A. iPSCs
SOX2
SOX17
High
Relative expression level
Low
B. PGCLCs
NANOS3
SOX17
PGCLCs
C. E74 Ovary
POU5F1
DDX4
PGC_E74
D. E82 Ovary
POU5F1
DDX4
PGC_E82
E. New born ovary
POU5F1
DDX4
MKI67
STRA8
PGC_NB
Oogonia_NB
SYCP1
PADI6
Oocyte_NB
F. E74 testes
POU5F1
DDX4
G. E87 testes
POU5F1
DDX4
PGC_E87
H. 22-day testes
POU5F1
DDX4
Gonocyte_22d

Figure S4

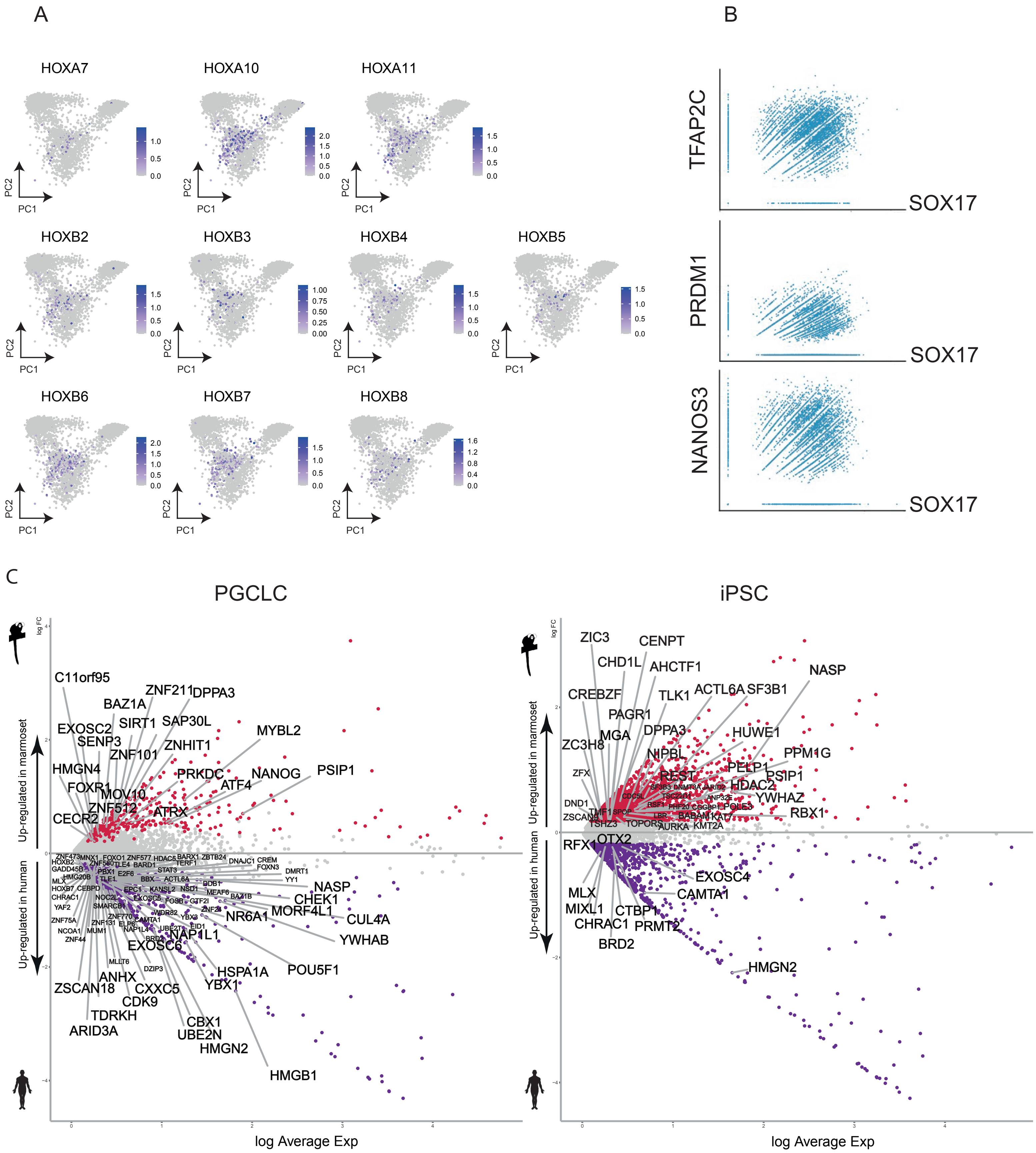
A
HOXA7
HOXA10
HOXA11
HOXB2
HOXB3
HOXB4
HOXB5
HOXB6
HOXB7
HOXB8
PC1
PC2
B
TFAP2C
PRDM1
NANOS3
SOX17
C
PGCLC
iPSC
Up-regulated in marmoset
Up-regulated in human
log FC
log Average Exp
C11orf95
ZNF211
DPPA3
BAZ1A
EXOSC2
SIRT1
SAP30L
MYBL2
SENP3
ZNHIT1
ZNF101
HMGN4
PRKDC
NANOG
PSIP1
ATF4
FOXR1
MOV10
ZNF512
CECR2
ATRX
NASP
CHEK1
MORF4L1
CUL4A
NR6A1
YWHAB
NAP1L1
EXOSC6
HSPA1A
POU5F1
ANHX
YBX1
ZSCAN18
CXXC5
CDK9
TDRKH
ARID3A
CBX1
UBE2N
HMGN2
HMGB1
ZIC3
CENPT
CHD1L
AHCTF1
CREBZF
TLK1
ACTL6A
SF3B1
NASP
PAGR1
MGA
DPPA3
HUWE1
ZC3H8
NIPBL
PPM1G
PELP1
PSIP1
HDAC2
YWHAZ
RBX1
REST
RFX1
OTX2
EXOSC4
MLX
CAMTA1
MIXL1
CTBP1
CHRAC1
PRMT2
BRD2
HMGN2

Figure S5

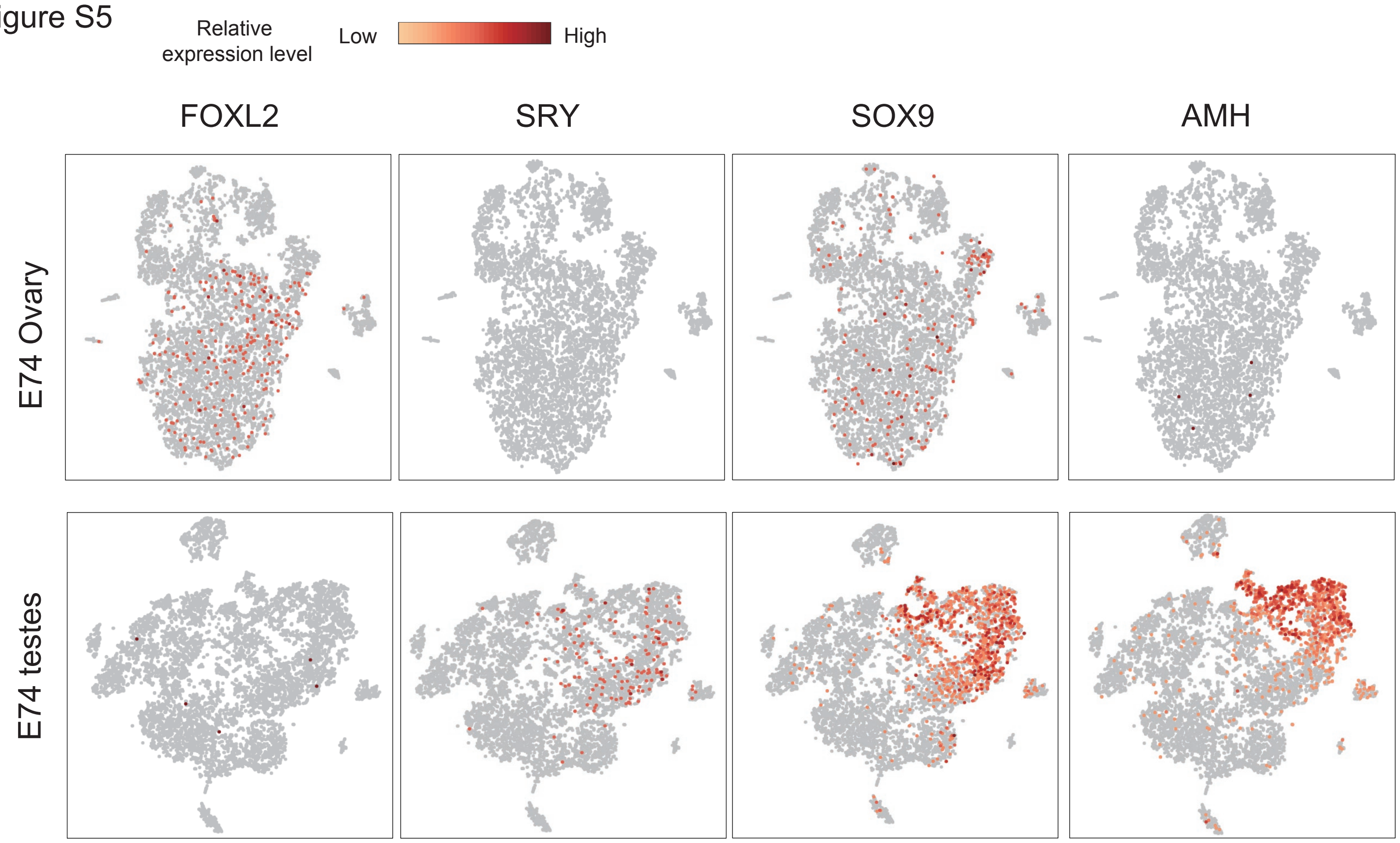

Relative
expression level
Low
High
FOXL2
SRY
SOX9
AMH
E74 Ovary
E74 testes

Figure S6

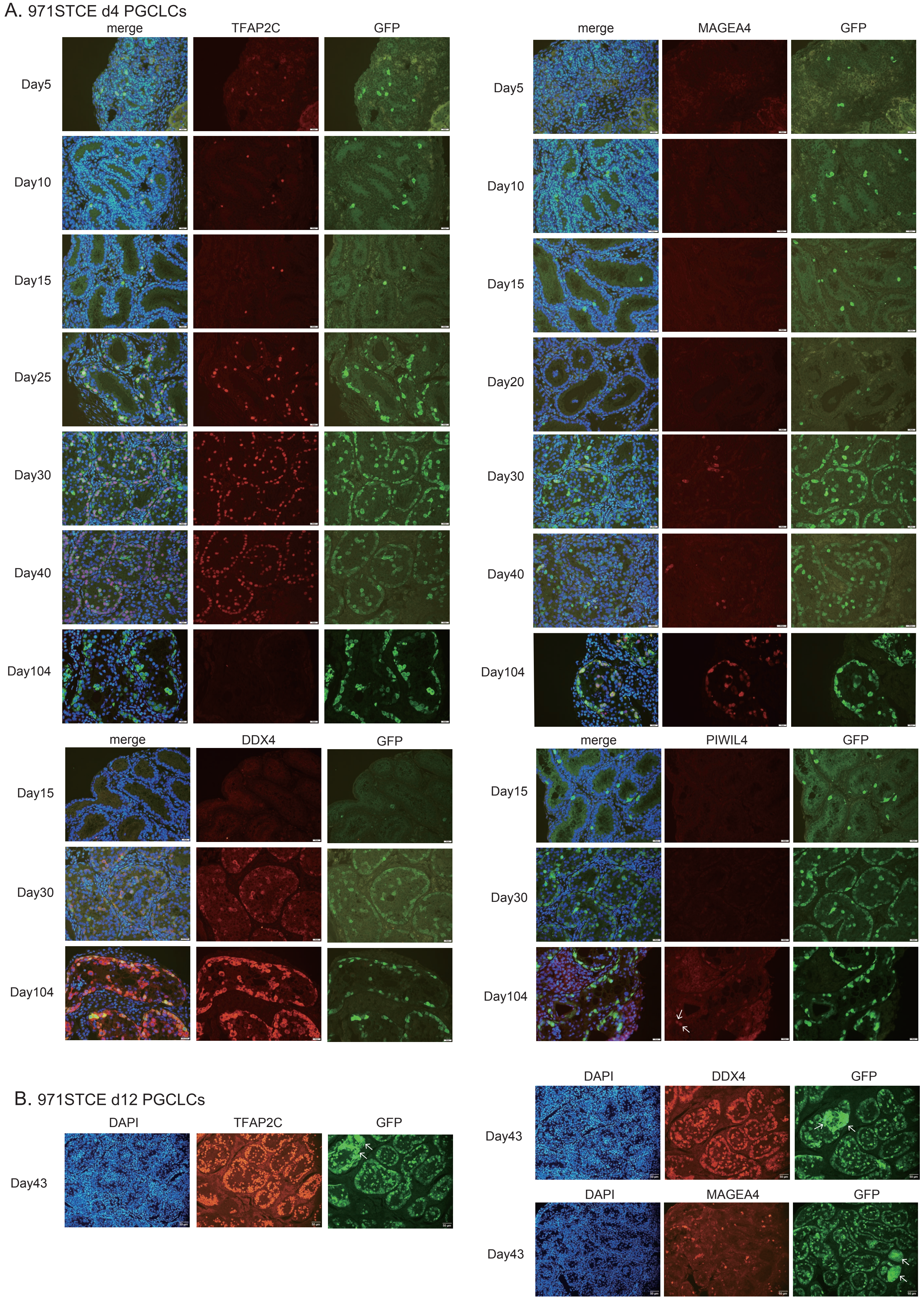
A. 971STCE d4 PGCLCs
merge
TFAP2C
GFP
Day5
Day10
Day15
Day25
Day30
Day40
Day104
merge
MAGEA4
GFP
Day5
Day10
Day15
Day20
Day30
Day40
Day104
merge
DDX4
GFP
Day15
Day30
Day104
merge
PIWIL4
GFP
Day15
Day30
Day104
B. 971STCE d12 PGCLCs
DAPI
TFAP2C
GFP
Day43
DAPI
DDX4
GFP
Day43
DAPI
MAGEA4
GFP
Day43

Figure S7

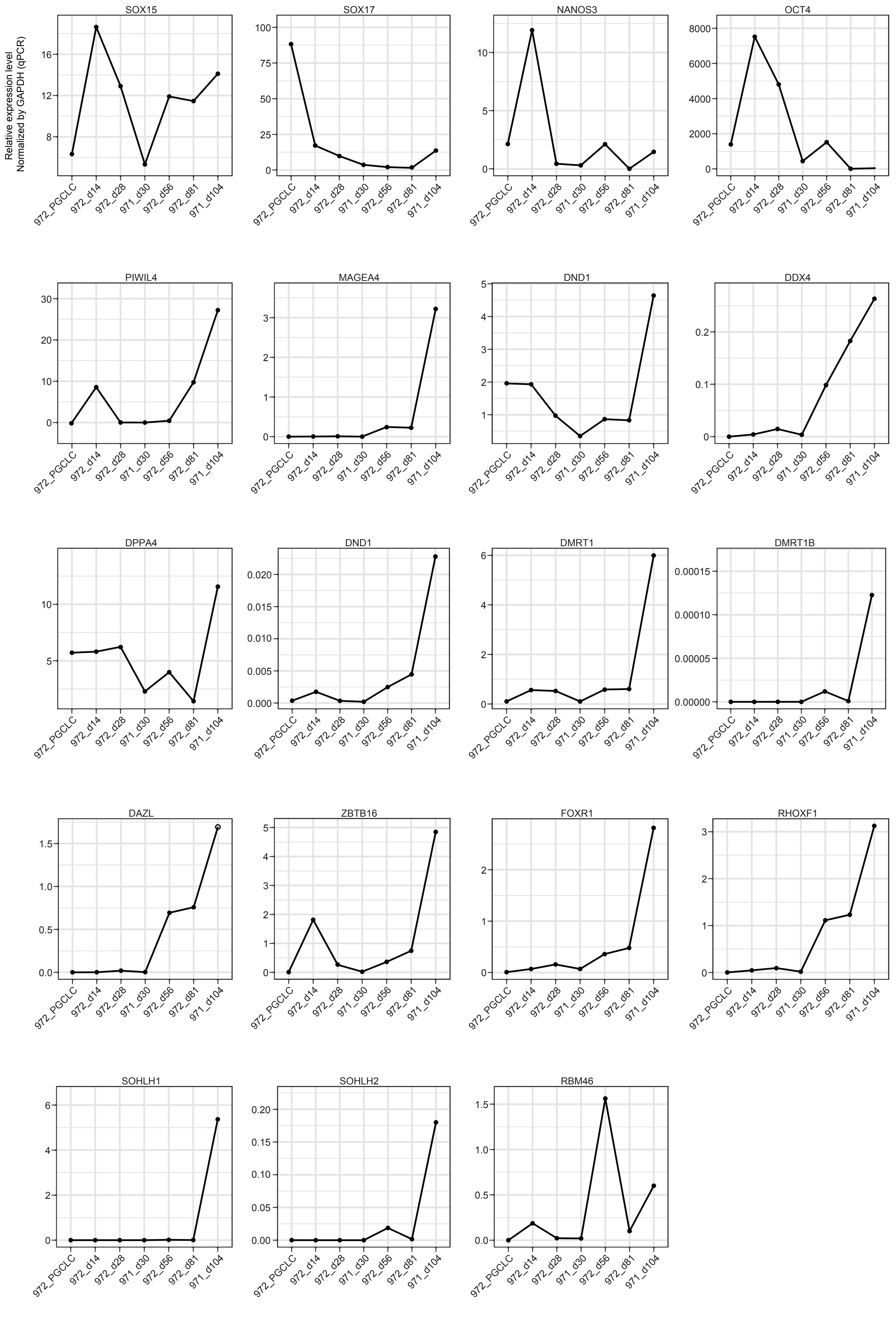
Relative expression level
Normalized by GAPDH (qPCR)
SOX15
SOX17
NANOS3
OCT4
PIWIL4
MAGEA4
DND1
DDX4
DPPA4
DND1
DMRT1
DMRT1B
DAZL
ZBTB16
FOXR1
RHOXF1
SOHLH1
SOHLH2
RBM46
972_PGCLC
972_d14
972_d28
971_d30
972_d56
972_d81
971_d104

Supplemental Figure Legends

Figure S1. PGCLC generation from 971-STCE and 972-STCE iPSCs

A. Schematic representation of knock-in of T2A-tdTomato cassette into *SOX17* locus. The expected recombination confirmed by PCR analyses (left and center). Heterozygosity (971 and 972) or homozygosity (mRNA) was checked by PCR indicated on the right. **B.** *SOX17*-tdTomato (ST) and CAG-EGFP (CE) expression in d4_PGCLCs from 971-STCE and 972-STCE iPS cells. Scale bars: 100 µm (top) and 50 µm (bottom). **C.** FACS analysis of d4_PGCLCs from 971-STCE and 972-STCE iPS cells. Many cells are positive for *SOX17*-tdTomato fluorescence. Antibodies for BV421 (Y-axis) fluorescence were not used here. **D.** FACS analyses of d4, d8, d16 PGCLCs. Fluorescent observation of aggregates used for FACS analyses. N=1. **E.** BrdU incorporation into *SOX17*-tdTomato-positive cells of d8_ and d16_ PGCLC aggregates (bottom). BrdU were added 48 hr before the sampling for immunohistochemical analyses. Proliferating PGCLCs are indicated by white arrows in the merge panels. Scale bars: 20 µm. N=1. **F.** Expression changes of key genes during PGCLC culture. FACS-purified PGCLCs were examine by qPCR. Error bars represent S.D. (N=2) of cells of different passage numbers. cDNAs used in Figure 1C were examined.

Figure S2. Determining the conditions for PGCLC induction using mRNAs

A. Determining the optimal number of transfections. In each well of 12-well plate, $8x10^4$ iPSCs (mRNA iPSCs) were seeded. As shown in the schematic diagram, after the indicated number of mRNA transfections, cells were aggregated in the low binding 96 well plate. The results of two independent experiments are shown. The expression levels of *NANOS3* and *GAPDH* in aggregates (day 4) were determined using qPCR. Delta Ct values are shown. The indicated values are the average of technical duplicates or triplicates. The results of two independent experiments are shown. **B.** Determining the optimal cell number of iPSCs (mRNA-ST iPSCs) used for transfection. PGCLC induction was performed using a standard procedure (two successive day mRNA transfection and aggregate culture for four days). PGCLC aggregates shown on the top were analyzed by qPCR (bottom). The average of technical triplicates are shown (N=1) separately. **C.** *SOX17* and *BLIMP1* mRNAs were together transfected at various ratios. After the two successive day transfection, PGCLCs were generated using floating aggregate culture. d4_PGCLCs were used for the analyses. The average of technical triplicates are shown (N=1).

Figure S3. Single cell RNA-seq analyses of developing marmoset iPSCs (A), PGCLC aggregates (B), ovaries (C-E) and testes (F-H)

iPSC and Germ cell populations used for PGCLC developmental analyses are indicated.

Figure S4. Characteristics of marmoset PGCLCs

A. PCA plot analyses of *HOX* genes. See Figure 2A for information of cells. **B.** Co-expression of *SOX17* with other markers *TFAP2C/PRDM1/NANOS3* in PGCLCs. PGCLCs identified in the analyses of Figure 2E were examined. **C.** Species specific expression of transcription factors in human and marmoset PGCLCs (left) and iPSCs (right). PGCLCs and iPSCs extracted from 10X data were analyzed. Transcription factors were indicated in the MA plot.

Figure S5. The expression of genes involved in sex differentiation in E74 ovaries and testes

Figure S6. The expression of PGC and gonocyte marker genes in xrtestes

Immunofluorecent analyses of marker gene expression in developing xrtestes. Day4 (A) and Day 12 (B) PGCLCs from 971-STCE iPSCs were used for the generation of xrtestes. Days after the transplantation were indicated on the left side of the panels. Xrtestis experiments conducted were listed in Table S2. Scale bar: 20 μm. *PIWIL4*-positive cells are indicated by white arrows in (A). Cancerous cells are indicated by white arrows in (B). The cancerous cells highly express EGFP, though germ cells express it only weakly. In addition, they do not express a PGC marker (*TFAP2C*), which is expressed in almost all seminomas (Pauls et al., 2005).

Figure S7. qPCR analyses of PGC and gonocyte marker genes in xrtestes

qPCR analyses of marker gene expression in developing xrtestes (971-STCE and 972-STCE). Only one sample was examined in each stage, and the indicated values are the average of technical duplicates. The expression level was normalized to that of *GAPDH*. The value was further normalized to the expression level in the marmoset adult testes. The expression level in the adult testes corresponds to 1.

Table S1. Sample and cell line information used in this study

Samples used for 10x library generation

#	Cells or samples (cell line name)	Library type	No. of cells analyzed[a]	Animal ID	Sex	Oocyte ID	Sperm ID	Note
1	iPSC culture (mRNA iPS)	10x v3	4,138	I 2965F	F			Derived from liver cells.
2	PGCLC aggregate (mRNA iPS)	10x v3	7,284	I 2965F	F			Induced from #1
3	E74 ovaries	10x v2	8,001		F	I4698F or I5463F	I4213M or YI034M	
4	E74 testes	10x v2	8,664		M	I4698F or I5463F	I4213M or YI034M	
5	E82 ovaries	10x v3	5,970		F	I689F	YI810M	
6	E87 testes	10x v2	7,230		M	I4750F or I4962F	I5057M or I764M	
7	Newborn ovaries	10x v2	14,308	From RIKEN	F			
8	22 day testes	10x v2	9,789	I 880M	M			
9	3yr10mnth testes	10x v2	12,335	I 6093M	M			

[a] Number of cells after Cellranger analyses

Samples used for simultaneous scRNA/scBS-seq aanalyses

#	Cells or samples	Library type	No. of cells analyzed	Animal ID	Sex	Oocyte ID	Sperm ID	Note
9	iPS/PGCLC (971-STCE)	scRNA-seq/scBS-seq	29 scRNA-seq/30 scBS-seq	I 971M	M			Derived from ear cells.<br>(RNA-seq/0 BS-seq[b])<br>iPS 3/0[b]<br>d4_PGCLC 2/3[c]<br>d12_PGCLC 2/3[c]<br>d5_xrtestes 3/3<br>d10_xrtestes 2/3[c]<br>d15_xrtestes 1/2[c]<br>d20_xrtestes 3/4[c]<br>d25_xxrtestes 3/2[c,d]<br>d30_xrtestes 3/3<br>d104_xrtestes 8/8
10	iPS/PGCLC (972-STCE)			I 972M	M			Derived from ear cells.

[b] scRNA-seq was only conducted

[c] For one sample, scBS-seq was only conducted

[d] For two samples, scRNA-seq was only conducted

Table S2. Summary of transplantation experiments

Cell line	PGCLCs	Sampling	Status	Marker expression	scRNA/scBS-seq
971 STCE	d4	d5	reconstituted testes		○
971 STCE	d4	d10	reconstituted testes		○
971 STCE	d15	d10	reconstituted testes		
972 STCE	d4	d12	reconstituted testes		
971 STCE	d4	d14	reconstituted testes	TFAP2C(+)	
972 STCE	d4	d14	reconstituted testes	TFAP2C(+)	
972 STCE	d4	d14	reconstituted testes		
971 STCE	d4	d15	reconstituted testes	TFAP2C(+)	○
971 STCE	d4	d20	reconstituted testes	TFAP2C(+)	○
971 STCE	d15	d21	reconstituted testes	TFAP2C(+)	
971 STCE	d4	d25	reconstituted testes	TFAP2C(+)	○
972 STCE	d4	d28	reconstituted testes	TFAP2C(+), DDX4(+)	
972 STCE	d4	d28	reconstituted testes		
971 STCE	d4	d28	reconstituted testes		
971 STCE	d4	d28	canceration		
971 STCE	d4	d30	reconstituted testes	TFAP2C(+), DDX4(+)	○
971 STCE	d4	d40	reconstituted testes	TFAP2C(+), DDX4(+)	
971 STCE	d4	d40	canceration		
971 STCE	d4	d42	reconstituted testes		
971 STCE	d12	d43	reconstituted testes and partial canceration	TFAP2C(+), DDX4(+), MAGEA4(+)	
972 STCE	d4	d47	reconstituted testes		
972 STCE	d4	d56	reconstituted testes	TFAP2C(+), DDX4(+), MAGEA4(+)	
972 STCE	d4	d56	reconstituted testes		
972 STCE	d4	d63	canceration		
972 STCE	d4	d81	reconstituted testes	DDX4(+), MAGEA4(+)	
972 STCE	d4	d81	reconstituted testes		
971 STCE	d4	d84	reconstituted testes and partial canceration	TFAP2C(+), DDX4(+)	
971 STCE	d4	d84	reconstituted testes and partial canceration		
971 STCE	d4	d84	reconstituted testes	TFAP2C(+), DDX4(+)	
971 STCE	d4	d84	reconstituted testes	TFAP2C(+), DDX4(+)	
972 STCE	d4	d90	canceration		
972 STCE	d4	d90	canceration		
972 STCE	d4	d90	reconstituted testes	TFAP2C(+), DDX4(+)	

972 STCE	d4	d90	reconstituted testes and partial canceration	TFAP2C(+), DDX4(+)	
972 STCE	d4	d96	reconstituted testes		
971 STCE	d4	d104	reconstituted testes and partial canceration	DDX4(+), MAGEA4(+), PIWIL4(+)	○
971 STCE	d15	d109	canceration		
971 STCE	d4	d113	reconstituted testes		
972 STCE	d4	d137	canceration		
972 STCE	d4	d137	canceration		
972 STCE	d4	d165	canceration		
972 STCE	d4	d165	canceration		
972 STCE	d4	d165	canceration		

Table S3. Summary statics for simultaneous scRNA-seq and scBS-seq analyses

scBS-seq	% of DNAme	% of mapping	# of total reads	
PGCLCs_d4-1	62.4	22.2	9271248	*scBS-seq only
PGCLCs-d4-2	60.3	29.5	5395186	
PGCLCs-d4-3	60.6	31.5	7753392	
PGCLCs_d12-1	42.8	38.9	7654536	
PGCLCs_d12-2	48.6	28.1	6006990	
PGCLCs_d12-3	36.5	22.3	3636159	*scBS-seq only
d5-1	43.3	30.4	7842101	
d5-3	39.3	19.5	6601570	
d5-4	47.8	24.2	8367382	
d10-1	28.4	34.1	6996434	
d10-2	40.3	19.9	5676850	*scBS-seq only
d10-8	31.8	31.2	4445992	
d15-3	11.7	33.5	5630184	*scBS-seq only
d15-8	33.3	33.2	7078191	
d20-1	19.2	29.6	5579726	*scBS-seq only
d20-2	17.5	23.4	5201037	
d20-3	14.6	35.8	5801722	
d20-4	10.2	31.7	7145941	
d25-1	10.9	19.2	5130107	*scBS-seq only
d25-8	8	29.9	7365294	
d30-1	11.4	39.2	5419758	
d30-3	7.9	30.9	6966065	
d30-4	8.9	30.1	6806686	
d104-2	3.2	1.6	5378037	
d104-21	4.2	1.9	4513287	
d104-36	5.1	2.1	5780035	
d104-62	3.6	2.8	7248465	
d104-74	3.8	3.6	5372929	
d104-108	6.6	5	5413307	
d104-139	3.5	6.3	5284600	
d104-144	4.3	9.7	6700814	

total:31 cells

scRNA-seq	# of total genic UMIs	# of total genes	Index sequences	Cell barcodes	
PGCLC_d4-2	13265	2860	103:Biotin/CAAGCAGAAGACGGCATACGAGATAGAGTAGTGACTG GAGTTCAGACGTGTGCTCTTCCGATC	ACAGCTACACGTGAGA	
PGCLC_d4-3	55505	5832	103:Biotin/CAAGCAGAAGACGGCATACGAGATAGAGTAGTGACTG GAGTTCAGACGTGTGCTCTTCCGATC	AGCGTATAGACGACGT	
PGCLC_12-1	8917	2929	105:Biotin/CAAGCAGAAGACGGCATACGAGATGCCAATGTGACTG GAGTTCAGACGTGTGCTCTTCCGATC	CACATAGTCGCACTCT	
PGCLC_12-2	11069	3311	105:Biotin/CAAGCAGAAGACGGCATACGAGATGCCAATGTGACTG GAGTTCAGACGTGTGCTCTTCCGATC	CCTACACTCTACTATC	
d5-1	9196	1948	106:Biotin/CAAGCAGAAGACGGCATACGAGATCTTGTAGTGACTG GAGTTCAGACGTGTGCTCTTCCGATC	GACACGCTCAGTCAGT	
d5-3	4519	1769	105:Biotin/CAAGCAGAAGACGGCATACGAGATGCCAATGTGACTG GAGTTCAGACGTGTGCTCTTCCGATC	GCAATCACAATGTTGC	
d5-4	28842	4036	104:Biotin/CAAGCAGAAGACGGCATACGAGATGCCAATGTGACTG GAGTTCAGACGTGTGCTCTTCCGATC	GGTGCGTAGGTGCACA	
d10-1	11944	3055	104:Biotin/CAAGCAGAAGACGGCATACGAGATGCCAATGTGACTG GAGTTCAGACGTGTGCTCTTCCGATC	GTTCTCGTCTGCTGTC	
d10-8	23130	3551	104:Biotin/CAAGCAGAAGACGGCATACGAGATGCCAATGTGACTG GAGTTCAGACGTGTGCTCTTCCGATC	AGCGTATAGACGACGT	
d15-8	20608	3958	103:Biotin/CAAGCAGAAGACGGCATACGAGATAGAGTAGTGACTG GAGTTCAGACGTGTGCTCTTCCGATC	ATCATCTGTAGCGTAG	
d20-2	15090	3372	104:Biotin/CAAGCAGAAGACGGCATACGAGATGCCAATGTGACTG GAGTTCAGACGTGTGCTCTTCCGATC	CACATAGTCGCACTCT	
d20-3	17330	3941	103:Biotin/CAAGCAGAAGACGGCATACGAGATAGAGTAGTGACTG GAGTTCAGACGTGTGCTCTTCCGATC	CCTACACTCTACTATC	
d20-4	64849	3929	104:Biotin/CAAGCAGAAGACGGCATACGAGATGCCAATGTGACTG GAGTTCAGACGTGTGCTCTTCCGATC	CGAGCACAGATAGCAT	
d25-3	13434	2887	103:Biotin/CAAGCAGAAGACGGCATACGAGATAGAGTAGTGACTG GAGTTCAGACGTGTGCTCTTCCGATC	GACACGCTCAGTCAGT	*scRNA-seq only
d25-4	8558	2976	104:Biotin/CAAGCAGAAGACGGCATACGAGATGCCAATGTGACTG GAGTTCAGACGTGTGCTCTTCCGATC	GCAATCACAATGTTGC	*scRNA-seq only
d25-8	24894	4146	104:Biotin/CAAGCAGAAGACGGCATACGAGATGCCAATGTGACTG GAGTTCAGACGTGTGCTCTTCCGATC	CCTACACTCTACTATC	
d30-1	72746	6109	103:Biotin/CAAGCAGAAGACGGCATACGAGATAGAGTAGTGACTG GAGTTCAGACGTGTGCTCTTCCGATC	GGTGCGTAGGTGCACA	
d30-3	20653	4516	103:Biotin/CAAGCAGAAGACGGCATACGAGATAGAGTAGTGACTG GAGTTCAGACGTGTGCTCTTCCGATC	GTTCTCGTCTGCTGTC	
d30-4	16235	3492	103:Biotin/CAAGCAGAAGACGGCATACGAGATAGAGTAGTGACTG GAGTTCAGACGTGTGCTCTTCCGATC	TACTCATCACAGTCGC	
d104-2	48848	6112	105:Biotin/CAAGCAGAAGACGGCATACGAGATGCCAATGTGACTG GAGTTCAGACGTGTGCTCTTCCGATC	ACAGCTACACGTGAGA	
d104-21	14859	4370	106:Biotin/CAAGCAGAAGACGGCATACGAGATCTTGTAGTGACTG GAGTTCAGACGTGTGCTCTTCCGATC	CACATAGTCGCACTCT	
d104-36	31217	5183	105:Biotin/CAAGCAGAAGACGGCATACGAGATGCCAATGTGACTG GAGTTCAGACGTGTGCTCTTCCGATC	ATCATCTGTAGCGTAG	
d104-62	22689	4778	105:Biotin/CAAGCAGAAGACGGCATACGAGATGCCAATGTGACTG GAGTTCAGACGTGTGCTCTTCCGATC	TCGGTAATCACGCATA	
d104-74	12425	4039	106:Biotin/CAAGCAGAAGACGGCATACGAGATCTTGTAGTGACTG GAGTTCAGACGTGTGCTCTTCCGATC	GCAATCACAATGTTGC	
d104-108	12594	4458	106:Biotin/CAAGCAGAAGACGGCATACGAGATCTTGTAGTGACTG GAGTTCAGACGTGTGCTCTTCCGATC	GTTCTCGTCTGCTGTC	
d104-139	32917	3962	106:Biotin/CAAGCAGAAGACGGCATACGAGATCTTGTAGTGACTG GAGTTCAGACGTGTGCTCTTCCGATC	GGTGCGTAGGTGCACA	
d104-144	12421	4160	106:Biotin/CAAGCAGAAGACGGCATACGAGATCTTGTAGTGACTG GAGTTCAGACGTGTGCTCTTCCGATC	TGTCCCAGTTTGGCGC	
iPSC-1	26593	2868	106:Biotin/CAAGCAGAAGACGGCATACGAGATCTTGTAGTGACTG GAGTTCAGACGTGTGCTCTTCCGATC	ACAGCTACACGTGAGA	*scRNA-seq only
iPSC-2	81459	6495	106:Biotin/CAAGCAGAAGACGGCATACGAGATCTTGTAGTGACTG GAGTTCAGACGTGTGCTCTTCCGATC	AGCGTATAGACGACGT	*scRNA-seq only
iPSC-3	115155	6873	104:Biotin/CAAGCAGAAGACGGCATACGAGATGCCAATGTGACTG GAGTTCAGACGTGTGCTCTTCCGATC	ATCATCTGTAGCGTAG	*scRNA-seq only

total: 30 cells

Table S4. The list of primer sequences used in this study

#	Name	
1	Sox17_5UTR	CTCAGAGAGAACCCACCACC ATGAGCAGCCCGGATG
2	Sox17_R_Asc1	TTAAC GGCGCGCC TCACACATCAGGATAGTTGCAG
3	5UTR_HBA_Not1	GATCGTACGCGGCCGCTCTTCTGGTCCCCACAGACTCAGAGA GAACCCAC
4	Sox17 5' arm F	ACGCGTTACGTATCGGATCC TGCAGCACATGCAGGACCAC
5	Sox17 5' arm R	CTGCCCTCTCCGGATCCCACATCAGGATAGTTGCAGTAATAC AC
6	Sox17 3' arm F	CGCTAGCGAATTCTAGGATCC GGA GAG CTAAGGAAGTCCTCAG
7	Sox17 3' arm R	ACGAATTCAGATTCGGATCC AGCCTCTGTAGGCAGGTCAAG
8	sgRNA-Universal-rev-primer	GTTT GAATTC AAAAAAA GCACCGACTCGGTGCCACTTTTTCAAGTTGATAA CGGACTAGCCTTATTTTAACTTGCTATTTCTAGCTCTAA
9	pHL_Sox17_1	CTT GGATCC G AAGCAGTGTTACACACTTCC GTTTTAGA GCTA GAAA TAGCA
10	pHL_Sox17_2	CTT GGATCC G ACACACTTCCTGGAGAGCTA GTTTTAGA GCTA GAAA TAGCA
11	pHL_Sox17_3	CTT GGATCC G GAGGACTTCCTTAGCTCTCC GTTTTAGA GCTA GAAA TAGCA
12	Sox17_scr5F	CAAGTCGTGGAAGGCGCTGAC
13	tdTomatoR1	GAAGCGCATGAACTCTTTGATGACCTC
14	Sox17_scr3R	AGGCAAACTCAACGTTGAGGAGTG
15	human-b-actProR	GACATCTCTTGGGCACTGAG
16	SOX15-qPCR F	CTACTCGACAGCCTACTTGCC
17	SOX15-qPCR R	CTGGAGCCTAGGGTCACTCTG
18	PIWIL4-qPCR F	CAGGTTCCAGTGGAATACCTGTG
19	PIWIL4-qPCR R	CACATGGTACTGGTATAGCTG
20	MAGEA4-qPCR F	TGGGAGGAGCTGAGTGTGATG
21	MAGEA4-qPCR R	CAGAGATACATCTCCAAGTCACTC
22	DAZL-qPCR F	CTCCGGCTTATTCATCTGTAAACT
23	DAZL-qPCR R	GATGCACTCTTTTATCCCTGAAGT
24	DMRT1B-qPCR F	TGCAGTAGGCCCTGAGTACC
25	DMRT1B-qPCR R	GGTAGTAGCTTGGCTGGAAGTCTC
26	FOXR1-qPCR F	GCAGAAACTTGCCAGGTATAAACT
27	FOXR1-qPCR R	GATCCTCCTTCTTCATAAGTCCTG
28	RHOXF1-qPCR F	GTGTTAACAAGAAGGGAACTTGCT

29	RHOXF1-qPCR R	ATGTAGAAACTGTCATCTGGATCG
30	SOHLH1-qPCR F	CTCTGGTGACTGTGGGTCCTA
31	SOHLH1-qPCR R	CGTCAACTGTAGAACATTCTCCTT
32	ZBTB16-qPCR F	ACTATAGGGTGCACACAGGTGAGA
33	ZBTB16-qPCR R	GAGGTACGTCTTCTCTATCCTCCA
34	CREM-qPCR F	GTGGAACAATCCAGATTTCTAACC
35	CREM-qPCR R	GTAGTAGGAGCTCGGATCTGGTAA
36	DMRT1-qPCR F	ATTCTTACTACCCACCTCCCTCTT
37	DMRT1-qPCR R	ATGACAGGAGTGACTGTAAAGCTG
38	SOHLH2-qPCR F	CAGGACATGCAGGTGATATGAC
39	SOHLH2-qPCR R	CTAGAGATTCAGGGCAGGCAGA
40	DND1-qPCR F	GGGCAGATCGCTCTGCTC
41	DND1-qPCR R	CTCTCCACAGAGGTGTGATTG
42	SOX17-qPCR F	CCGAGCTGAGCAAGATGCTG
43	SOX17-qPCR R	GTGGTCCTGCATGTGCTGCAC
44	NANOS3-qPCR F	CTTCTGCCCACTCACTGGACAG
45	NANOS3-qPCR R	CTCAGACTTCCCGGCACCTCTG
46	DDX4-qPCR F	AAGTATTAACAGATGCTCAACAGGATGT
47	DDX4-qPCR R	TGAAGCCAGGAATGTATGCACTA
48	DPPA4-qPCR F	TGGGTAAGCAAAGGCACACAG
49	DPPA4-qPCR R	CTGGTGTCAGCAACTAAAGCTAAGCAC
50	GAPDH-qPCR F	TGCTGGCGCTGAGTATGTG
51	GAPDH-qPCR R	AGCCCCAGCCTTCTCCAT
52	SOX17-endo-F	ACCGCGACAGGCTAGAACAC
53	SOX17-endo-R	GGTCTGGCTCTGGTCGTCAC
54	SOX17-exo-F	CCCACAGACTCAGAGAGAAC
55	SOX17-exo-R	GGTCTGGCTCTGGTCGTCAC
56	NANOG-qPCR F	TGCTGAGATGCCTCACACAG
57	NANOG-qPCR R	TCACTGCAGGGCTACTCTCT
58	DPPA3-qPCR-F	CGGTCATCAGTTTCTGCTGTG
59	DPPA3-qPCR-R	TCCTGGTAAGTTTCTCCGGTG
60	OCT4-qPCR F	GGAACAAAACACGGAGGAGTC
61	OCT4-qPCR R	CAGGGTGATCCTCTTCTGCTTC
62	BLIMP1-qPCR F	CGGATATGACTCTGTGGACAGAG
63	BLIMP1-qPCR R	CACGCCAATAACCTCTTTGCTG
64	CB16UMI12-RT primer1	TCA GAC GTG TGC TCT TCC GAT CTA ATC GGT GTT GAT TCG NNN NNN NNN NNN TTT TTT TTT TTT TTT TTT TTT TTT T
65	BC16UMI12-RT primer2	TCA GAC GTG TGC TCT TCC GAT CTA CAG CTA CAC GTG AGA NNN NNN NNN NNN TTT TTT TTT TTT TTT TTT TTT TTT T

66	BC16UMI12-RT primer3	TCA GAC GTG TGC TCT TCC GAT CTA GCG TAT AGA CGA CGT NNN NNN NNN NNN TTT TTT TTT TTT TTT TTT TTT TTT T
67	BC16UMI12-RT primer4	TCA GAC GTG TGC TCT TCC GAT CTA TCA TCT GTA GCG TAG NNN NNN NNN NNN TTT TTT TTT TTT TTT TTT TTT TTT T
68	BC16UMI12-RT primer5	TCA GAC GTG TGC TCT TCC GAT CTC ACA TAG TCG CAC TCT NNN NNN NNN NNN TTT TTT TTT TTT TTT TTT TTT TTT T
69	BC16UMI12-RT primer6	TCA GAC GTG TGC TCT TCC GAT CTC CTA CAC TCT ACT ATC NNN NNN NNN NNN TTT TTT TTT TTT TTT TTT TTT TTT T
70	BC16UMI12-RT primer7	TCA GAC GTG TGC TCT TCC GAT CTC GAG CAC AGA TAG CAT NNN NNN NNN NNN TTT TTT TTT TTT TTT TTT TTT TTT T
71	BC16UMI12-RT primer8	TCA GAC GTG TGC TCT TCC GAT CTC TGA AGT AGT ATT GGA NNN NNN NNN NNN TTT TTT TTT TTT TTT TTT TTT TTT T
72	BC16UMI12-RT primer9	TCA GAC GTG TGC TCT TCC GAT CTG ACA CGC TCA GTC AGT NNN NNN NNN NNN TTT TTT TTT TTT TTT TTT TTT TTT T
73	BC16UMI12-RT primer10	TCA GAC GTG TGC TCT TCC GAT CTG CAA TCA CAA TGT TGC NNN NNN NNN NNN TTT TTT TTT TTT TTT TTT TTT TTT T
74	BC16UMI12-RT primer11	TCA GAC GTG TGC TCT TCC GAT CTG GTG CGT AGG TGC ACA NNN NNN NNN NNN TTT TTT TTT TTT TTT TTT TTT TTT T
75	BC16UMI12-RT primer12	TCA GAC GTG TGC TCT TCC GAT CTG TTC TCG TCT GCT GTC NNN NNN NNN NNN TTT TTT TTT TTT TTT TTT TTT TTT T
76	BC16UMI12-RT primer13	TCA GAC GTG TGC TCT TCC GAT CTT ACT CAT CAC AGT CGC NNN NNN NNN NNN TTT TTT TTT TTT TTT TTT TTT TTT T
77	BC16UMI12-RT primer14	TCA GAC GTG TGC TCT TCC GAT CTT CGG TAA TCA CGC ATA NNN NNN NNN NNN TTT TTT TTT TTT TTT TTT TTT TTT T
78	BC16UMI12-RT primer15	TCA GAC GTG TGC TCT TCC GAT CTT GTC CCA GTT TGG CGC NNN NNN NNN NNN TTT TTT TTT TTT TTT TTT TTT TTT T
79	BC16UMI12-RT primer16	TCA GAC GTG TGC TCT TCC GAT CTT TGA CTT GTA CTC GCG NNN NNN NNN NNN TTT TTT TTT TTT TTT TTT TTT TTT T
80	TSO primer	/5Me-isodC//iisodG//iMe-isodC/AAGCAGTGGTATCAACGCAGAGTACATrGrG+G
81	3' Anchoerd primer	GTGACTGGAGTTCAGACGTGTGCTCTTCCGATC
82	ISPCR primer	AAGCAGTGGTATCAACGCAGAGT
83	scBS-P5-N9-oligo1	CTACACGACGCTCTTCCGATCTNNNNNNNNN
84	scBS-oligo2	GACTGGAGTTCAGACGTGTGCTCTTCCGATCTNNNNNNNNN
85	Biotin-index-primer103	Biotin/CAAGCAGAAGACGGCATACGAGATAGAGTAGTGACTGGAGTTCAGACGTGTGCTCTTCCGATC

86	Biotin-index-primer104	Biotin/CAAGCAGAAGACGGCATACGAGATAGTCCAGTGACTGGAGTTCAGACGTGTGCTCTTCCGATC
87	Biotin-index-primer105	Biotin/CAAGCAGAAGACGGCATACGAGATGCCAATGTGACTGGAGTTCAGACGTGTGCTCTTCCGATC
88	Biotin-index-primer106	Biotin/CAAGCAGAAGACGGCATACGAGATCTTGTAGTGACTGGAGTTCAGACGTGTGCTCTTCCGATC
89	QP2	CAAGCAGAAGACGGCATACGA
90	Modified_P5 primer	AATGATACGGCGACCACCGAGATCTACAC[index]ACACTCTTTCCCTACACGAC

Supplemental Experimental Procedures

Marmoset housing and samplings

All animal experiments using marmosets and mice were approved by the Animal Committee of the Central Institute for Experimental Animals (CIEA) (Approval number; 17029A, 18031A, 19033A, 21002A, 21012A, and 21052A) and National Center for Child Health and Diseases (NCCHD). Marmosets (CLEA Japan) were housed in CIEA and NCCHD. The marmosets were housed in stainless steel cages (W436 x D750 x H765 mm to W910 x D750 x H2050 mm) under the following conditions: temperature, 27 °C, humidity 40%, room pressure, +20 hPa, light 12 h per day, and basic food CMS-1 (CLEA Japan).

To obtain fetal gonads (E74 and E82 ovaries and E87 testes), frozen or fresh early embryos (8-cell to morula) were transferred into recipient uteri (Takahashi et al., 2014). The developmental days were determined based on the day of ovulation when the serum progesterone of the recipient animals exceeded 10 ng/ml. Fetuses were obtained by C-sections. The pregnant female marmosets were pre-anesthetized with 0.04 mg/kg medetomidine (Domitor, Nippon Zenyaku Kogyo), 0.40 mg/kg midazolam (Dormicam, Astellas Pharma), and 0.40 mg/kg butorphanol (Vetorphale, Meiji Seika Pharma), then sedated by isoflurane (Isoflurane inhalation solution, Viatris) inhalation. While C-section, the animals were warmed on a warmer pad on the surgery table. The uterus was exteriorized via a midline laparotomy and lifted from the abdominal cavity, and the fetus was removed from the uterus. After C-section, the female marmoset's abdominal wall and skin were sutured and kept warm in the intensive care unit until awakened from anesthesia.

Testis from 22d marmosets was obtained by hemicastration under anesthesia by inhalation of 1–3% isoflurane. Newborn ovaries and adult testes were obtained from animals sacrificed for use in other experiments and for illnesses that could not be cured, respectively.

mRNA preparation

For *in vitro* transcription of *SOX17* mRNA, marmoset *SOX17* cDNA was PCR-amplified from testis cDNAs using a following set of primers: Sox17_5UTR and Sox17_R_Asc1. The PCR product was again amplified to include the human *HBA* gene 5' UTR by using a following set of primers: 5UTR_HBA_Not1 and Sox17_R_Asc1. The primer sequences are listed in Table S4. The product was digested using Asc1 and Not1 and then ligated to phBG vector (deposited in RIKEN BRC #RDB18330) (Watanabe et al., 2019) to generate phBG-SOX17 vector. phBG-SOX17 and previously generated phBG-P53DD (Addgene #149707) (Watanabe *et al.*, 2019) vectors were digested with HindIII for *in vitro* transcription using mMESSAGE mMACHINE T7 Ultra Kit (Ambion). *In vitro* transcribed mRNAs were purified using LiCl precipitation, and they were used for the transfection.

Induction of PGCLCs

iPSCs (lines 971 and 972) were induced from ear fibroblast cells as described previously (Watanabe et al., 2019). Lipofection-based mRNA transfection was performed to generate PGCLCs from iPSCs. The day before transfection (day 0), the iPSCs were dissociated into single cells by Accumax (17087-54; Nacalai Tesque) at 37 °C and then 5.0×10^4 marmoset iPSCs were plated per one well of 12-well cell culture plates in MEF-conditioned medium containing 1.25 µg/ml iMatrix-511 silk (892021; FUJIFILM) and 10 µM Y-27632 (08945-42; Nacalai Tesque). The next day (day 1), the medium was replaced with 500 µL of the new conditioned medium. For transfection, two tubes containing 62.5 µL of OPTI-MEM (A4124801; Invitrogen) were prepared. In one tube, RNAs [0.2 µg of SOX17 mRNA, 0.05 µg of P53DD mRNA, 0.075 µg of the mixture of E3, K3, B18R mRNAs (00-0076; Reprocell)] were added. In the other tube, 1.5 µL of Lipofectoamine RNAiMax Transfection Reagent (13778150; Invitrogen) was added. After mixing well, the solutions in the two tubes were mixed, and the mixture was allowed to stand for 10 min. Next, the mixture was added dropwise into one well of a 12-well plate. The next day (day 2), the medium was replaced with 500 µL of new conditioned medium, and RNA transfection was performed again. Medium change and RNA transfection was performed at 10 am and 4 pm, respectively.

On the morning of day 3 (~10 am), cells were dissociated using Accumax. Subsequently, ~5,000 cells were plated in each well of a Nunclon Spher 96-Well plate (174929; Thermo Fisher Scientific) in 100 µL of aRB27 (1% B27/2 mM L-glutamine/1× non-essential amino acids/1× antibiotic-antimycotic in advanced RPMI medium) containing 50 ng/mL EGF (236-EG-200; R&D Systems), 100 ng/mL SCF (455-MC-050; R&D Systems), 1,000 U/mL LIF (LIF1010; Merck), 400 ng/mL BMP4 (314-BP-500; R&D Systems), and 10 µM Y-27632. The medium was changed every 4 days. BrdU (30 µg/ml) was added 48 hr before the sampling.

Lentivirus preparation and infection

Lentivirus was prepared using a protocol on the RIKEN website (https://dnaconda.riken.jp/Form_PDF/lntPrepen.pdf). Briefly, CS-CA-GFP (RDB05964), pCMV-VSV-G-RSV-Rev (RDB04393), and pCAG-HIVgp (RDB04394) were co-transfected into HEK293T cells. The supernatant was collected to enrich the lentivirus using LentiX-concentrator (Takara, 631231). The virus was stored at -80ºC until use. For the infection of the virus, the virus was added to the medium together with 4 µg/ml of polybrene. Then, spinfection was performed at 800 xg for 30 minutes at 32ºC.

Knock-in of reporter gene into *SOX17* gene locus

To visualize the generation of PGCLCs, T2A-tdTomato was inserted into the C-terminus of the *SOX17* gene. To make the targeting vector, 820-bp 5' and 669-bp 3' arms were amplified from the genome and inserted into the BamHI-digested KW1521 vector using an In-fusion HD cloning kit (Takara, 639648). The KW1521 vector contains T2A-tdTomato cassette and human beta-actin promoter driven Neomycin resistant gene cassette for the selection. For generating sgRNA-expressing vectors, PCR was performed using a common primer (sgRNA-Universal-rev-primer) and specific primers (pHL_Sox17_1, pHL_Sox17_2, pHL_Sox17_3). PCR products were then digested with EcoRI and BamHI, and ligated to the EcoRI and BamHI sites of pHL-H1-ccdB-mEF1a-RiH vector (Addgene #60601) (Li et al., 2015).

For homologous recombination, iPS cells were plated at the concentration of 8×10^4 cells per one well of 6-well plate at one day before the transfection (Day 0). Transfection was performed using the following plasmid DNAs [1 µg of targeting vector, 0.5 µg of pHL-EF1a-SphcCas9-iP-A (Addgene #60599) (Li *et al.*, 2015), and 167 ng each of three sgRNA vectors (pHL_H1_Sox17_1, pHL_H1_Sox17_2, pHL_H1_Sox17_3)] using Lipofectoamine 3000 reagent (Thermo Fisher Scientific, L3000008) or Lipofectoamine stem transfection reagent (Thermo Fisher Scientific, STEM00015) according to the manufacture's protocol. The day after the transfection (Day 2), cells were newly plated onto one well of a six-well plate. From Day 3, the selection was performed using 150 µg/ml G418. Colony pickup was performed ~Day 12.

For isolation of DNA for genotyping, cells were treated with DNA isolation buffer [100 mM Tris-HCl (PH 7.6)/100 mM NaCl/10 mM EDTA (PH 8.0)/0.5% SDS] containing 100 µg/ml Proteinase K at 50°C. After the phenol-chloroform extraction, DNA was precipitated by adding an equal volume of isopropanol at room temperature. PCR was performed to detect knock-in using the following sets of primers: Sox17_scr5F and tdTomatoR1 (recombination at the coding region side), Sox17_scr3R and human-b-actProR (recombination at the 3' UTR side), and Sox17_scr5F and Sox17_scr3R (for detecting entire knock-in insertion or wildtype bands).

Karyotype analysis

The iPSCs were cultured for 3 h in a medium containing 0.1 µg/mL colcemid (15212012; Gibco) and dissociated into single cells by treatment with Accumax. After collection, the cells were treated with 0.075 M KCl for 30 min at 25°C. Subsequently, the cells were fixed using a fixing solution (70% methanol/30% acetic acid). The fixed cells were then spread onto a glass slide using HANABI and stained with Hoechst 33342 (H1399; Invitrogen). Karyotype images were obtained using a Leica 6000B microscope. All three iPSC lines used in this study had normal karyotype.

mRNA ST: 46 XX 80% (5/5)

971 STCE: 46 XY 87.5% (7/8)

972 STCE: 46 XY 87.5% (7/8)

FACS purification of PGCLCs

PGCLC aggregates were dissociated with 0.25% Trypsin-EDTA for 10 minutes at 37 °C. The reaction was stopped by adding an equal amount of 10% KSR/alpha-MEM. DNaseI was added at the final concentration of 25 μg/ml, and cells were left at room temperature for 1 minute before centrifuge. After adding FACS buffer containing DAPI for removing dead cells, cells were filtered using a 60 μm cell strainer. tdTomato-positive cells were purified using SONY SH800 using purity mode.

RT-qPCR

RNA was extracted using TRIzol Reagent (15596026; Invitrogen). RNA was converted to cDNA using Superscript IV Reverse Transcriptase VILO Master Mix with ezDNase enzyme (11766050; Invitrogen), and gene expression analysis was performed using the primers listed in Table S4.

Immunofluorescence

xrtestes collected from kidneys and testes were fixed in 4% PFA (09154-56; Nacalai Tesque), and then they were embedded into paraffin blocks. Afterward, sections (4 μm) were deparaffinized and rehydrated using a xylene and ethanol series. For antigen retrieval, slides were heated to 95 °C for 10 min in 0.01 M citrate buffer (pH 6.0). The sections were blocked using a primary antibody diluent (AR9352; Leica Biosystems) and incubated overnight at 4 °C with the following primary antibodies: Rabbit-anti-GFP (308SS; 1:1,000; Novus Biologicals), Goat-anti-GFP (ab5450; 1:3,000; Abcam), anti-LAMININ (L9393; 1:200; Sigma-Aldrich), anti-3 β -HSD (sc-515120; 1:1,500; Santa Cruz Biotechnology Inc), anti-WT1 (ab89901; 1:500; Abcam), anti-MKI67 (NCL-ki67p; 1:50; Leica Biosystems), anti-TFAP2C (sc-12762; 1:100; Santa Cruz Biotechnology Inc), anti-DDX4 (AF2030; 1:500; R&D Systems), anti-MAGEA3/4 (MABC1150; 1:250; Merck), anti-PDPN (337001; 1:200; Biolegend), anti-BrdU (Bu20a; 1:200; Dako), and anti-PIWIL4 (GP1831 produced in a guinea pig; AHSSFRATEVGRTQD-Cys; 1:50). Subsequently, the sections were incubated with species-specific secondary antibodies for 60 min at room temperature.

***In vivo* differentiation**

To isolate fetal testicular somatic cells, pregnant ICR females were sacrificed, and embryos were collected at E13.5. Fetal male testes were distinguished from ovaries by their appearance, and the mesonephros attached to the testes were removed using a tungsten needle. Thereafter, the isolated

testes were treated with 0.25% trypsin EDTA (25200056; Nacalai Tesque) for 10 min at 37 °C for dissociation into single cells. To remove endogenous mouse PGCs, cells were plated in a cell culture plate in MEM α (12571063; Gibco) containing 10% KSR (10828-028; Gibco) and cultured for 6 h (Ohinata et al., 2009). PGCs usually do not attach to plates; the attached cells were removed from the culture plate using 0.25% trypsin/EDTA and collected as fetal testicular somatic cells. The proportions of germ cells in the attached and unattached fractions were 3.3% and 69.1%, respectively (determined by *DDX4* immunostaining).

Marmoset d4 or d12_PGCLCs were purified using *SOX17*-T2A-tdTomato fluorescence and FACS. The collected d4 or d12_PGCLCs (5.0×10^3 cells) were mixed with E13.5 fetal testicular somatic cells (5.0×10^4 cells) and plated in an ultra-low attachment 96-well plate (174929; Thermo Fisher Scientific) with MEM α containing 10% KSR, 1× antibiotic-antimycotic and 10 μM Y27632. The next day, cell aggregates were transplanted under the kidney capsule of NOG (NOD/Shi-scid, IL-2RγKO) mice to promote differentiation (Ito et al., 2002). Thereafter, NOG mice were anesthetized using medetomidine, midazolam, and butorphanol, and an incision was made in the peritoneum to expose the kidney capsule. Next, the kidney capsule was carefully incised using an injection needle under an inverted microscope, and cell aggregates were picked up using a glass capillary and transplanted into the kidney capsule.

Single cell RNA-seq (10X)

After dissection, mesonephros, epididymis and tunica albuginea were removed from testes and ovaries. Testes and ovaries were cut in pieces using forceps or scalpels. The iPS cells were dissociated using Accumax, while PGCLCs, ovaries (E74, E82, and newborn), and testes (E87 and 22d) were dissociated using 0.25% trypsin EDTA for 10 min at 35°C. Cell dissociation was promoted by gentle pipetting or tapping. Adult testes (3 years 10 months) were digested stepwise with 30 min collagenase 1 (1mg/mL) treatment and 10 min trypsin (0.25%) EDTA treatment. Trypsin reaction was stopped by adding 10% FCS/DMEM. DNaseI was, then, added at the final concentration of 20 μg/ml. Newborn ovaries were not completely digested by the above procedure, while others were digested almost completely. Cell suspension were filtered through 70 μm cell strainer. Cells were then washed using 0.04% BSA/PBS three times. For iPS cells, PGCLCs, and E82 ovaries, libraries were constructed using a Chromium Next GEM Single Cell 3′ GEM Library & Gel Bead Kit v3 (10x Genomics). Other libraries were constructed using a Chromium Single Cell 3′ Library and Gel Bead Kit v2 (10x Genomics), and sequencing was performed using a HiSeq4000 system (Illumina). One library was made from each stage.

To analyze 10x data, datasets were mapped to the common marmoset genome, calJac4 (or calJac3), using CellRanger. NCBI Callithrix jacchus Annotation Release 105 was used to obtain UMI count data. The UMI count data were analyzed using Seurat. Normalization and

standardization were performed using *NormalizeData* and *ScaleData* functions. Principal component analysis was performed using the inbuilt *RunPCA* function. Dimensionality reduction was visualized in 2D using *DimPlot*.

Comparison of *in vitro* datasets with *in vivo* dataset and Human datasets

Marmoset 10X datasets were mapped to the common marmoset genome (Callithrix jacchus 3.2.1) using CellRanger 3.0.2, which was used filter out low-quality cells and empty droplets. Gene annotation files for common marmoset genome (Caljac 3.2.1) were downloaded from Ensembl (release 91) and gene models extended similarly to the approach that was reported (Bergmann et al., 2022; Boroviak et al., 2018).

For comparison with human, several existing human 10x datasets were also analyzed, including germ cell samples from neonatal testes (Sohni et al., 2019) (GSE124263) and PGCLCs induced from iPSCs (Chen et al., 2019) (GSE140021). Smart-seq2 (SS2) datasets of male and female fetal germ cells (Li et al., 2017) (GSE86146) were incorporated in downstream analyses alongside single cell data from human iPSC and PGCLCs (Kojima et al., 2017) (GSE99350).

All samples that passed QC were analyzed using Seurat v3.1.2 (Butler et al., 2018). UMI data was normalized and standardised using the *NormalizeData* and *ScaleData* function using either the top 2,000, 5000 or 20,000 most varied genes for initial analysis. Principal component analysis was run using the inbuilt *RunPCA* function, with nonlinear dimensionality reduction techniques generated using RunUMAP. Dimensionality reduction was visualised in 2D using DimPlot.

Data from marmoset and humans were incorporated together for an integrative view of germ cell development. First our 10X samples from iPSCs were combined with samples from PGCLC aggregates to create a marmoset *in vitro* reference. Each gene in the marmoset data was annotated by its human orthologue based on the Ensembl BioMart database (https://m.ensembl.org/info/data/biomart/index.html). These homologous genes were used for the integrative analyses of Human and marmoset data. The marmoset reference was integrated alongside human ESC and aggregate datasets (Chen *et al.*, 2019), consisting of two replicated cell lines, one with high PGCLC-competence and one with low PGCLC-competence. The five datasets (our dataset, two replicates high competence line in human, two replicates low competence line) were then jointly aligned in Seurat based on Canonical Correlation Analysis (CCA) and mutual nearest neighbor (MNN) approaches. Specifically, FindIntegrationAnchors was run using 4000 features and In*tegrateData* (with 20 dimensions) was used to calculate corrected gene expression matrices for the three datasets. Datasets were visualized using PCA and UMAP on the corrected gene expression matrix, with expression plots based on the uncorrected RNA slot expression. Unbiased clustering on the aligned marmoset-human dataset was done using the *FindClusters* function. Recent re-analysis of these datasets alongside

appropriate *in vivo* references (Castillo-Venzor et al., 2022) identified a number of transient and terminal cell populations in the human aggregates, including primitive-streak-like cells, mesoderm-like cells, amnion-like cells, endoderm-like cells, and PGC-like cells. Cell identity assignment in the marmoset datasets could be assigned by co-clustering with the annotated human cells.

Subsequently, marmoset iPSC and PGCLC cells were merged with 10X samples of marmoset PGCs to create a marmoset PGC developmental trajectory reference dataset, which was integrated with 10X samples from human neonate gem cell lineages merged with 10X samples of human iPSCs and PGCLCs. SS2 datasets from male and female germ cells were merged with SS2 samples of PGCLCs and iPSCs. Unbiased clustering on the aligned marmoset-human dataset was done using the *FindClusters* function, and individual clusters were annotated based on the dominant lineages in the cluster. Cell lineages for marmosets were first identified in individual samples based on the expression of known marker genes (Figure S3). These annotations were additionally checked against the aligned human annotations and showed strong agreement.

Finally, marmoset lineages were appended by developmental stage, and the average expression of lineages was calculated using *AverageExpression*. Row normalized expression values for an extended panel of known markers was visualized using pheatmap.

RNA and DNA purification from the same single cell

xrtestes were cut in pieces using scalpels and they were dissociated using 0.25% Trypisn-EDTA. xrtestis cells were resuspended in 0.04% BSA in PBS for single-cell pick up. Single-cell (EGFP+) was picked up by a mouth pipet and put into a PCR tube. Lysis buffer (1.14 U/μl Rnase Inhibitor, 0.54% Triton X-100) containing DynaBeads MyOne Carboxylic acid (Thermo Fisher) was added. Beads bound to the nucleus and supernatant corresponding to the cytoplasmic fraction were separated. DNA isolation buffer (20 mM Tris-EDTA, 20 mM KCl, 0.3% Triton X-100, 1mg/ml Proteinase K, 0.1 pg/μl λDNA, 2 ng/μl carrier RNA) was added to the beads, and they were incubated at 50°C for 10 minutes. Proteinase was then heat-inactivated by 75°C for 30 minutes.

Single-cell RNA-seq of the cytoplasmic fraction

Before cDNA synthesis, ERCC RNA (Invitrogen, 1:500,000) was added to the cytoplasmic RNA. cDNA was made from cytoplasmic RNAs using oligo dT primer with cell barcode and UMI sequences (CB16UMI12-RT), template switch (TS) oligo DNA, and SuperScript II Reverse transcriptase (Thermo Fisher). To prevent concatamerization of the TS oligo, non-natural nucleotides were added to its 5' end. PCR reaction was performed using 3' Anchoerd primer and ISPCR primers to amplify cDNA. After purification of cDNA using AMPure beads, the qPCR reaction was carried out using a primer pair that detected germ cell-specific genes (NANOS3 *and*

PIWIL4) for the selection of germ cell. After pooling 8 cDNAs with different barcodes, PCR was performed using a biotinylated primer bound to 5' end of oligodT sequence (Biotin-index-primer) and unmodified primer annealed to 5' end of TS oligo (ISPCR). Fragmentation was carried out using NEBNext Ultra II FS DNA Library Prep Kit for Illumina, and biotinylated DNA was purified using streptavidin beads. Adapter ligation and PCR-amplification (using QP2 and Modified_P5_primer) were then performed according to NEBNext Ultra II FS DNA Library Prep Kit for Illumina. After quality check using Bioanalyzer (Agilent), paired-end 150-bp sequencing was performed by Illumina HiSeqX. Primer sequences are listed in Table S4.

Single-cell BS-seq

Single-cell BS-seq was conducted based on published literatures (Bian et al., 2018; Smallwood et al., 2014; Zhou et al., 2019). Before the bisulfite reaction, non-methylated lamda DNA (Promega) was added to the DNA. For bisulfite conversion, MethylCode Bisulfite Conversion kit (Invitrogen) was used. For making single-cell BS-seq libraries, two rounds of random priming reaction were performed using a random primer with a nucleotide ratio of A:T:G:C = 4:4:1:1 (scBS-P5-N9-oligo1), which reduces bias toward preferential amplification of GC-rich sequences. After ExonucleaseI treatment for removing the remaining random primer, second-strand synthesis was performed using scBS-oligo2 primer. PCR amplification were then carried out using unique dual index primers (NEBNext Multiplex Oligos for Illumina, 96 Unique Dual Index). After quality check using a bioanalyzer (Agilent), paired-end 150-bp sequencing was performed by Illumina HiseqX.

scRNA/scBS-seq data analysis

Our custom scRNA libraries were designed for analyses using CellRanger. Prior to the analyses using CellRanger, Read1 and Read2 were switched by changing the file names. Datasets were mapped to the common marmoset genome calJac4 using CellRanger. For annotation of calJac4 genome, NCBI Callithrix jacchus Annotation Release 105 was used. UMI count data were analyzed using Seurat. The count matrix was created by *CreateSeuratObject* function. To extract the cells of interest, a *subset* function was used. Data from different libraries were combined using the *merge* function. To remove cells with a small number of UMIs, a subset function was used by setting a cutoff value of unique feature counts over 1500. Normalization and standardization were performed using the *NormalizeData* and *ScaleData* functions, respectively. Appropriate cell names were reassigned using the *changeindent* function. Variable genes were extracted by *FindVariableFeatures* and principal component analysis was run using the inbuilt *RunPCA* function. Heatmap was generated using a *pheatmap* package.

For scBS-seq analysis, quality of the library was checked by FastQC and trimmed away adaptor

sequence using the TrimGalore program using an option --quality 20 --stringency 3 --length 50 --clip_R1 9 --clip_R2 9 --paired --trim1 --phred33. Reads were mapped to the reference genome using Bismark with a command bismark --bowtie2 --fastq --non_directional --un. Duplicate reads were removed with *deduplicate* function in Bismark (deduplicate_bismark –bam). Deduplicated BAM files were then converted to bedGraph using *methylation extractor* (bismark_methylation_extractor -p --bedGraph -counts).

For retrotransposon analyses, the coordinates of 8,251 full-length LINE1 and 2,275 full length Platy-1 elements were retrieved from L1 base2 (l1base.charite.de/l1base.php) and a published literature(Konkel et al., 2016), respectively. The calJac3 coordinates were converted to calJac4 coordinates using a liftover tool in UCSC (Lift Genome Annotations (ucsc.edu)). The coordinates for three major retrotransposon classes were retrieved from repeatmasker file downloaded from UCSC. Methylation data in bedgraph file format were used to calculate the average methylation levels.

Supplemental References

Bergmann, S., Penfold, C.A., Slatery, E., Siriwardena, D., Drummer, C., Clark, S., Strawbridge, S.E., Kishimoto, K., Vickers, A., Tewary, M., et al. (2022). Spatial profiling of early primate gastrulation in utero. Nature. 10.1038/s41586-022-04953-1.

Bian, S., Hou, Y., Zhou, X., Li, X., Yong, J., Wang, Y., Wang, W., Yan, J., Hu, B., Guo, H., et al. (2018). Single-cell multiomics sequencing and analyses of human colorectal cancer. Science *362*, 1060-1063. 10.1126/science.aao3791.

Boroviak, T., Stirparo, G.G., Dietmann, S., Hernando-Herraez, I., Mohammed, H., Reik, W., Smith, A., Sasaki, E., Nichols, J., and Bertone, P. (2018). Single cell transcriptome analysis of human, marmoset and mouse embryos reveals common and divergent features of preimplantation development. Development *145*. 10.1242/dev.167833.

Butler, A., Hoffman, P., Smibert, P., Papalexi, E., and Satija, R. (2018). Integrating single-cell transcriptomic data across different conditions, technologies, and species. Nat Biotechnol *36*, 411-420. 10.1038/nbt.4096.

Castillo-Venzor, A., Penfold, C.A., Morgan, M.D., Tang, W.W.C., Kobayashi, T., Wong, F.C.K., Bergmann, S., Slatery, E., Boroviak, T.E., Marioni, J.C., and Surani, M.A. (2022). Origin and segregation of the human germline. bioRxiv, 2022.2007.2006.498671. 10.1101/2022.07.06.498671.

Chen, D., Sun, N., Hou, L., Kim, R., Faith, J., Aslanyan, M., Tao, Y., Zheng, Y., Fu, J., Liu, W., et al. (2019). Human Primordial Germ Cells Are Specified from Lineage-Primed Progenitors. Cell Rep *29*, 4568-4582 e4565. 10.1016/j.celrep.2019.11.083.

Ito, M., Hiramatsu, H., Kobayashi, K., Suzue, K., Kawahata, M., Hioki, K., Ueyama, Y., Koyanagi, Y., Sugamura, K., Tsuji, K., et al. (2002). NOD/SCID/gamma(c)(null) mouse: an excellent recipient mouse model for engraftment of human cells. Blood *100*, 3175-3182. 10.1182/blood-2001-12-0207.

Kojima, Y., Sasaki, K., Yokobayashi, S., Sakai, Y., Nakamura, T., Yabuta, Y., Nakaki, F., Nagaoka, S., Woltjen, K., Hotta, A., et al. (2017). Evolutionarily Distinctive Transcriptional and Signaling Programs Drive Human Germ Cell Lineage Specification from Pluripotent Stem Cells. Cell Stem Cell *21*, 517-532 e515. 10.1016/j.stem.2017.09.005.

Konkel, M.K., Ullmer, B., Arceneaux, E.L., Sanampudi, S., Brantley, S.A., Hubley, R., Smit, A.F., and Batzer, M.A. (2016). Discovery of a new repeat family in the Callithrix jacchus genome. Genome Res *26*, 649-659. 10.1101/gr.199075.115.

Li, H.L., Fujimoto, N., Sasakawa, N., Shirai, S., Ohkame, T., Sakuma, T., Tanaka, M., Amano, N., Watanabe, A., Sakurai, H., et al. (2015). Precise correction of the dystrophin gene in duchenne muscular dystrophy patient induced pluripotent stem cells by TALEN and CRISPR-Cas9. Stem Cell Reports *4*, 143-154. 10.1016/j.stemcr.2014.10.013.

Li, L., Dong, J., Yan, L., Yong, J., Liu, X., Hu, Y., Fan, X., Wu, X., Guo, H., Wang, X., et al. (2017). Single-Cell RNA-Seq Analysis Maps Development of Human Germline Cells and Gonadal Niche Interactions. Cell Stem Cell *20*, 858-873 e854. 10.1016/j.stem.2017.03.007.

Ohinata, Y., Ohta, H., Shigeta, M., Yamanaka, K., Wakayama, T., and Saitou, M. (2009). A signaling principle for the specification of the germ cell lineage in mice. Cell *137*, 571-584. 10.1016/j.cell.2009.03.014.

Pauls, K., Jager, R., Weber, S., Wardelmann, E., Koch, A., Buttner, R., and Schorle, H. (2005). Transcription factor AP-2gamma, a novel marker of gonocytes and seminomatous germ cell tumors. Int J Cancer *115*, 470-477. 10.1002/ijc.20913.

Smallwood, S.A., Lee, H.J., Angermueller, C., Krueger, F., Saadeh, H., Peat, J., Andrews, S.R., Stegle, O., Reik, W., and Kelsey, G. (2014). Single-cell genome-wide bisulfite sequencing for assessing epigenetic heterogeneity. Nat Methods *11*, 817-820. 10.1038/nmeth.3035.

Sohni, A., Tan, K., Song, H.W., Burow, D., de Rooij, D.G., Laurent, L., Hsieh, T.C., Rabah, R., Hammoud, S.S., Vicini, E., and Wilkinson, M.F. (2019). The Neonatal and Adult Human Testis Defined at the Single-Cell Level. Cell Rep *26*, 1501-1517 e1504. 10.1016/j.celrep.2019.01.045.

Takahashi, T., Hanazawa, K., Inoue, T., Sato, K., Sedohara, A., Okahara, J., Suemizu, H., Yagihashi, C., Yamamoto, M., Eto, T., et al. (2014). Birth of healthy offspring following ICSI in in vitro-matured common marmoset (Callithrix jacchus) oocytes. PLoS One *9*, e95560. 10.1371/journal.pone.0095560.

Watanabe, T., Yamazaki, S., Yoneda, N., Shinohara, H., Tomioka, I., Higuchi, Y., Yagoto, M., Ema, M., Suemizu, H., Kawai, K., and Sasaki, E. (2019). Highly efficient induction of primate iPS cells by combining RNA transfection and chemical compounds. Genes Cells *24*, 473-484. 10.1111/gtc.12702.

Zhou, F., Wang, R., Yuan, P., Ren, Y., Mao, Y., Li, R., Lian, Y., Li, J., Wen, L., Yan, L., et al. (2019). Reconstituting the transcriptome and DNA methylome landscapes of human implantation. Nature *572*, 660-664. 10.1038/s41586-019-1500-0.
