# Supplementary material for: mRNA-based generation of marmoset PGCLCs capable of differentiation into gonocyte-like cells
Source: Stem Cell Reports. 2023 Sep 7;18(10):1987–2002. doi: 10.1016/j.stemcr.2023.08.006 (PMC10656353; doi:10.1016/j.stemcr.2023.08.006)
Supplement: Document S2. Article plus supplemental information [file mmc2.pdf]

Stem Cell Reports
Resource
OPEN ACCESS

mRNA-based generation of marmoset PGCLCs capable of differentiation into gonocyte-like cells

Musashi Kubiura-Ichimaru,[1,2] Christopher Penfold,[3,4,5,6] Kazuaki Kojima,[1,7] Constance Dollet,[1,7] Haruka Yabukami,[8] Katsunori Semi,[9] Yasuhiro Takashima,[9] Thorsten Boroviak,[6] Hideya Kawaji,[10,11] Knut Woltjen,[9] Aki Minoda,[8,12] Erika Sasaki,[1] and Toshiaki Watanabe[1,7,*]

[1]Central Institute for Experimental Animals, 3-25-12 Tonomachi, Kawasaki-ku, Kawasaki 210-0821, Japan
[2]Division of Molecular Genetics & Epigenetics, Department of Biomolecular Science, Faculty of Medicine, Saga University, 5-1-1 Nabeshima, Saga 849-8501, Japan
[3]Department of Physiology, Development and Neuroscience, University of Cambridge, Downing Site, Cambridge, UK
[4]Wellcome Trust-Cancer Research UK Gurdon Institute, University of Cambridge, Cambridge, UK
[5]Centre for Trophoblast Research, University of Cambridge, Downing Site, Cambridge CB2 3EG, UK
[6]Wellcome Trust – Medical Research Council Stem Cell Institute, University of Cambridge, Jeffrey Cheah Biomedical Centre, Puddicombe Way, Cambridge CB2 0AW, UK
[7]National Center for Child Health and Development, Tokyo 157-8535, Japan
[8]Laboratory for Cellular Epigenomics, RIKEN Center for Integrative Medical Sciences, Yokohama, Kanagawa 230-0045, Japan
[9]Department of Life Science Frontiers, Center for iPS Research and Application (CiRA), Kyoto University, Kyoto 606-8507, Japan
[10]Research Center for Genome & Medical Sciences, Tokyo Metropolitan Institute of Medical Science, Tokyo 156-8506, Japan
[11]Preventive Medicine and Applied Genomics Unit, RIKEN Center for Integrative Medical Sciences, Yokohama, Kanagawa 230-0045, Japan
[12]Department of Cell Biology, Faculty of Science, Radboud Institute for Molecular Life Sciences, Radboud University, Nijmegen, the Netherlands
*Correspondence: watanabe-tos@ncchd.go.jp
https://doi.org/10.1016/j.stemcr.2023.08.006

SUMMARY

Primate germ cell development remains largely unexplored due to limitations in sample collection and the long duration of development. In mice, primordial germ cell-like cells (PGCLCs) derived from pluripotent stem cells (PSCs) can develop into functional gametes by *in vitro* culture or *in vivo* transplantation. Such PGCLC-mediated induction of mature gametes in primates is highly useful for understanding human germ cell development. Since marmosets generate functional sperm earlier than other species, recapitulating the whole male germ cell development process is technically more feasible. Here, we induced the differentiation of iPSCs into gonocyte-like cells via PGCLCs in marmosets. First, we developed an mRNA transfection-based method to efficiently generate PGCLCs. Subsequently, to promote PGCLC differentiation, xenoreconstituted testes (xrtestes) were generated in the mouse kidney capsule. PGCLCs show progressive DNA demethylation and stepwise expression of developmental marker genes. This study provides an efficient platform for the study of marmoset germ cell development.

INTRODUCTION

Marmosets (*Callithrix jacchus*) are New World monkeys native to Brazil that are often used in biomedical research, particularly in brain science, owing to their small size, relative ease of handling, high reproductive ability, and close evolutionary relationship to humans. In addition to these characteristics, marmosets reach puberty within 1 year of birth. This relatively short period of sexual maturation makes marmosets an ideal model organism for studying primate germ cell development. However, the high cost of studies using marmosets limits the number of animal experiments. Therefore, to complement *in vivo* studies, it is important to develop a tractable marmoset germ cell developmental system using pluripotent stem cells (PSCs).

Several studies have reported the induction of primordial germ cell-like cells (PGCLCs) from PSCs in primates (humans, macaques, and marmosets) (Irie et al., 2015; Sakai et al., 2020; Sasaki et al., 2015; Sosa et al., 2018; Yoshimatsu et al., 2021). Although complete differentiation of PGCLCs into sperm or eggs has been successful in mice, it has not yet been achieved in any primate species. In humans, PGCLCs differentiate into gonocytes after a few months of the *in vitro* culture of xenoreconstituted testes (xrtestes) generated from human PGCLCs and mouse embryonic gonadal somatic cells (Hwang et al., 2020). Furthermore, in rhesus macaques, male PGCLCs differentiate into MAGEA4-positive gonocytes by homologous transplantation into adult testes and xenotransplantation into seminiferous tubules of mouse testes (Sosa et al., 2018). Because both extrinsic and intrinsic factors determine developmental speed, the duration of germ cell development in the PGCLC system is likely to be influenced by the *in vivo* development timetable. Although early embryonic development is delayed in marmosets (Phillips, 1976), they reach sexual maturation earlier than other primates. Therefore, the marmoset PGCLC system may be more feasible for recapitulating the entire primate germ cell development process. Although PGCLCs have been generated in marmosets, the reported method requires *a priori* transgene integration for the forced expression of *SOX17* and *PRDM1* (Yoshimatsu et al., 2021). Furthermore, there are no reports on the differentiation of PGCLCs into *MAGEA4*-positive gonocytes in marmosets.

This is an open access article under the CC BY license (http://creativecommons.org/licenses/by/4.0/).

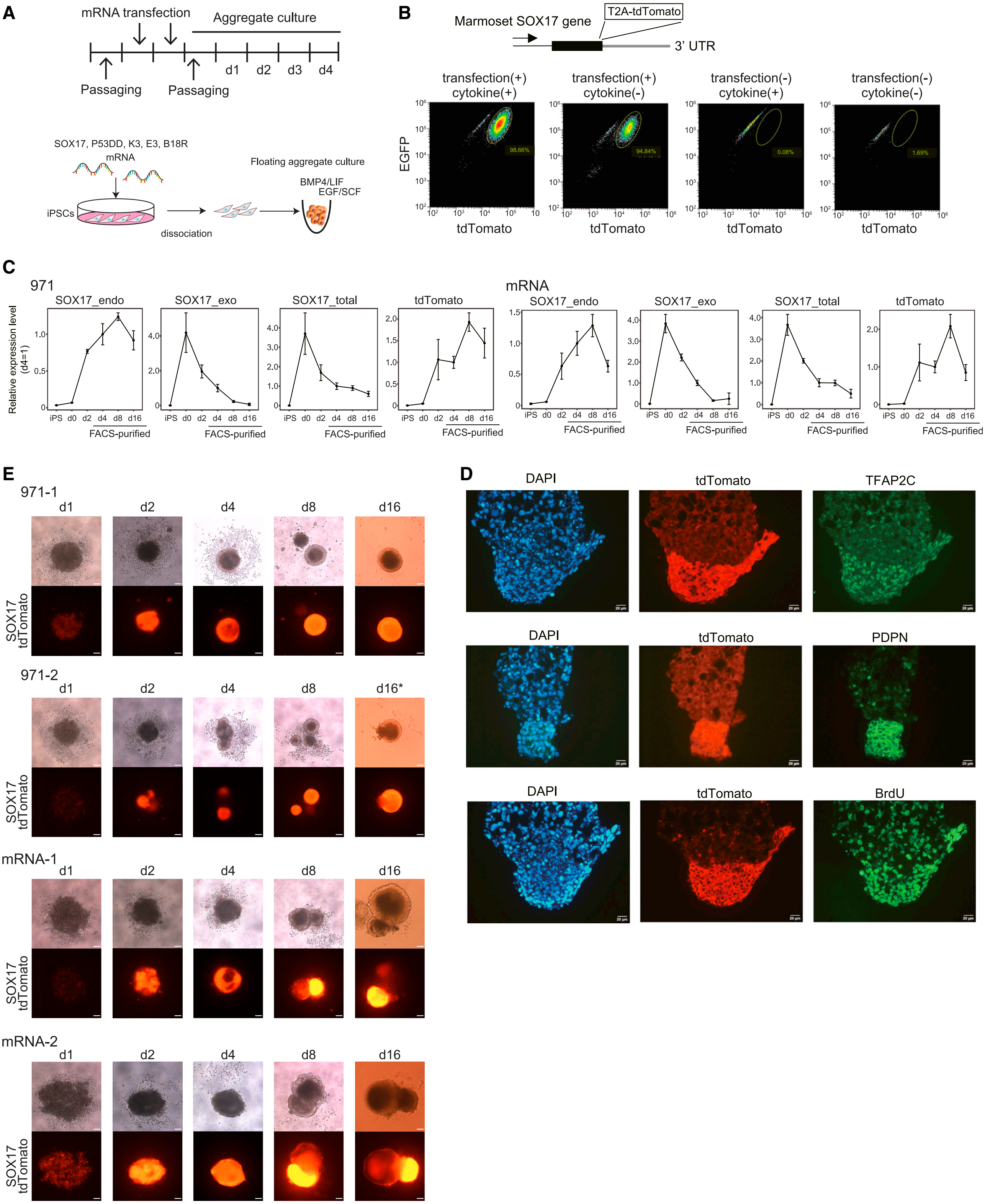

(legend on next page)

In addition to examining germ cell development, this PGCLC-initiated germ cell developmental system is useful for generating genetically modified animals. This is enabled by gamete production using this system from genetically modified PSCs. All genetically modified marmosets generated to date have been produced from zygotes (Park and Sasaki, 2020). Cultured cell-based systems, such as the PGCLC-mediated system, enable the creation of animals with complex genetic modifications such as reporter gene knock-in and multiple modifications. Furthermore, a cell-based system ensures the production of the expected genetic modifications. Thus, establishing a germ cell developmental system from PGCLCs to produce functional gametes may accelerate primate research.

In this study, to develop a PGCLC-initiated germ cell developmental system in marmosets, we developed a novel mRNA transfection-based method to convert PSCs into PGCLCs. Furthermore, these PGCLCs were differentiated into a gonocyte-like state using a transplantation approach. Our results provide the basis for studying marmoset gametogenesis using PGCLCs.

RESULTS

mRNA transfection-based induction of marmoset PGCLCs

To monitor the differentiation into PGC-like state, we inserted T2A-tdTomato into the C terminus of *SOX17* gene (*SOX17*-tdTomato [ST]) in one female (mRNA ST) and two male iPSC lines harboring CAG-EGFP (CE) transgenes (971 STCE and 972 STCE) (Figure S1A). We have previously reported mRNA transfection-based methods for marmoset induced PSC (iPSC) induction (Watanabe et al., 2019). To generate PGCLCs from iPSCs, we performed mRNA transfection. Because *SOX17* has been reported to have critical functions in PGCLC induction in humans (Irie et al., 2015; Kobayashi et al., 2017), we selected this mRNA for transfection. To alleviate the damage caused by mRNA transfection in cells, we transfected cells with interferon suppressors (vaccinia virus *E3*, *K3*, and *B18R* mRNAs) (Poleganov et al., 2015) and an apoptosis suppressor (mouse P53DD mRNA) (Hong et al., 2009).

After 2 successive days of transfection into 971 STCE iPSCs, the transfected cells were seeded onto low-attachment 96-well plates to form aggregates in medium containing LIF, EGF, stem cell factor (SCF), and BMP4 based on the procedures for human PGCLC induction (Figure 1A) (Hwang et al., 2020; Irie et al., 2015; Kojima et al., 2017; Sasaki et al., 2015; Yamashiro et al., 2018). Four days after making the aggregate, ST expression was observed in many cells in the aggregates (Figures 1B, S1B, and S1C), although the efficiency was somewhat varied among trials. ST expression was correlated with the endogenous *SOX17* expression (Figure 1C). Immunofluorescence analyses showed that these ST-positive cells co-expressed *TFAP2C* and *PDPN* (Figure 1D), suggesting that they are PGCLCs. In contrast, when mRNA transfection was omitted, ST expression was not observed (Figure 1B). This result indicates that mRNA transfection plays critical roles in PGCLC induction. Omission of growth factors slightly reduced the number of ST-positive cells and the level of ST fluorescence (Figure 1B). Thus, growth factors likely play some roles in PGCLC induction and/or maintenance in our induction system.

To examine the time course change, aggregate culture was continued with medium change every 4 days using a male iPSC line harboring *SOX17*-tdTomato transgene (971 STCE). ST expression was first observed 24 h after PGCLC aggregate formation (Figure 1E). A proportion of cells expressing ST fluorescence seemingly reached maximal levels at either day 4 or 8 (Figures 1E and S1D). Then, the proportions of the positive cells decreased at day 16 when we stopped the culture. However, a significant portion of cells still expressed ST, even on day 16. In ST-positive cells, exogenous *SOX*17 mRNAs were still observed at day 4, but they were barely detected by day 8 (Figure 1C), suggesting autonomous regulation of gene regulatory network at day 8. Proliferation of the ST-positive cell was observed on days 4, 8, and 16

Figure 1. mRNA-based generation of PGCLCs from iPSCs

(A) Schematic diagram for mRNA-mediated induction of PGCLCs from iPSCs.

(B) Requirement of mRNA transfection and growth factors. A T2A-tdTomato cassette was inserted into the C terminus of the *SOX17* gene to monitor differentiation into PGCLCs. The four charts represent the results of induction with the presence/absence of mRNA transfection and cytokines (n = 1).

(C) Time course qPCR analyses of total/exogenous/endogenous *SOX17* and tdTomato mRNA expression. PGCLC aggregates of two different cell lines (971 STCE and mRNA ST) were cultured for 16 days. A primer set detecting both exogenous and endogenous *SOX17* mRNAs was used for total *SOX17*. Error bars represent SD of the results of cells of different passage numbers (n = 2).

(D) Immunohistochemical analyses of the day 4 PGCLC aggregates. The expression of PGC markers (*TFAP2C*, *PDPN*) and BrdU incorporation were examined in *SOX17*-tdTomato-positive cells. BrdU was added to the medium 48 h before the sampling. Scale bars, 20 μm. n = 1.

(E) Time course examination of *SOX17*-tdTomato fluorescence (971 STCE and mRNA ST). Shown are PGCLC aggregates examined by the qPCR analyses in (C). Asterisk in 971-2: different PGCLC aggregates were shown for day 16 and the other stages. Scale bars, 100 μm.

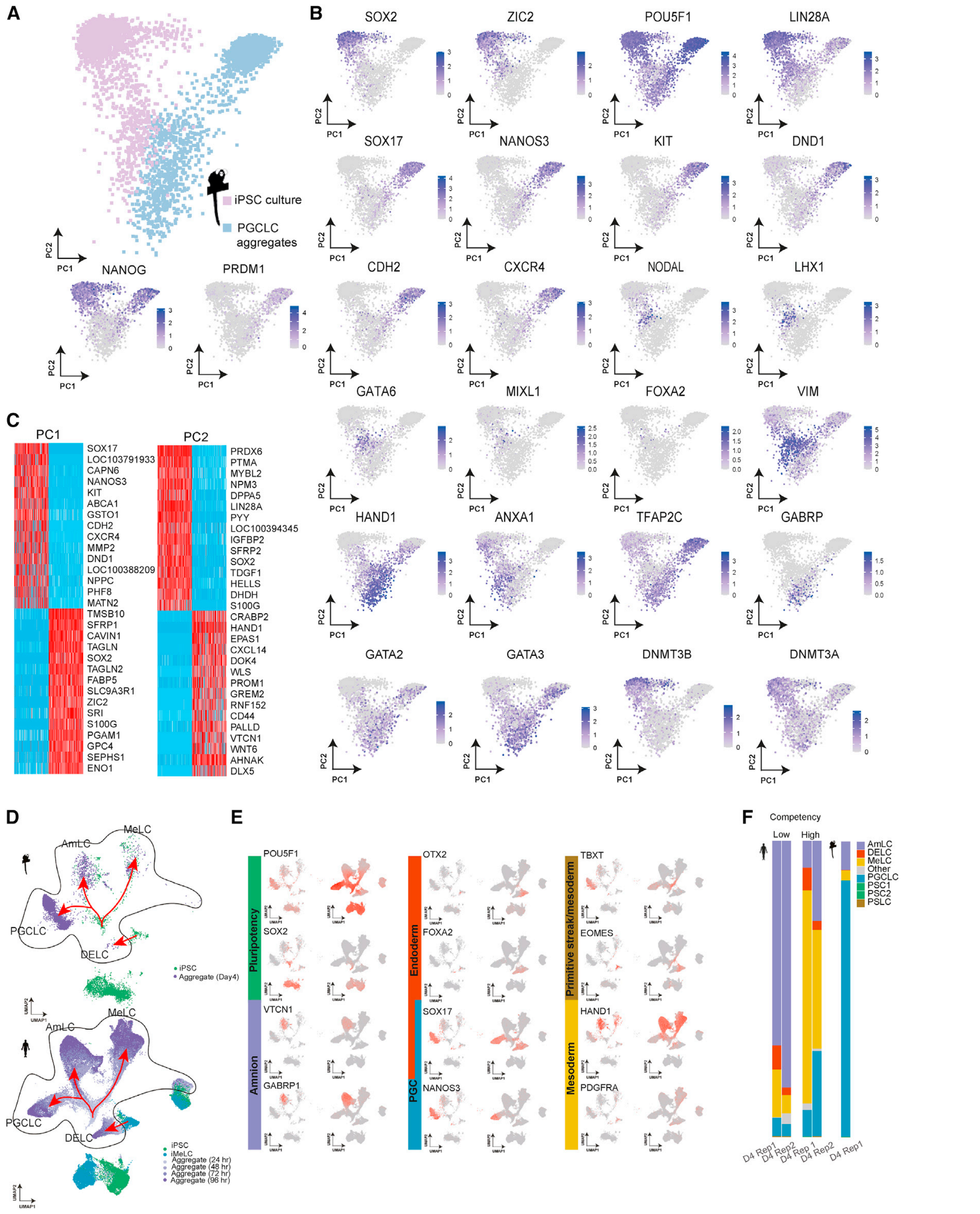

Figure 2. Gene expression analyses of marmoset PGCLCs induced from iPSCs
(A) Single-cell analyses of iPSCs (n = 1) and PGCLCs (n = 1).
(B) In addition to the iPSC (*SOX2* and *ZIC2*) and PGCLC markers (*SOX17*, *KIT*, *NANOS3*, and *DND1*), mesoendoderm-like cells (*NODAL* and *MIXL1*), a small number of endoderm-like cells (*SOX17* and *FOXA2*), mesenchymal-like cells (*HAND1*, *ANXA1*, and *VIM*), and amnion-like cells (*HAND1*, *TFA2PC*, and *GABRP*) were present.

(legend continued on next page)

(Figures 1D and S1E). The expression of PGC and pluripotent marker genes were observed in ST-positive cells during this period, although some decreases were observed on day 16 (Figure S1F).

The optimal number of mRNA transfections was determined to be two. Two successive days of transfections resulted in higher *NANOS3* expression in day 4 aggregates (d4_PGCLC) than did single transfection, but 3 successive day transfections did not result in increased *NANOS3* expression (Figure S2A). The optimal number of transfected cells was determined to be 50,000 cells per well in a 12-well plate (Figure S2B). Using an increased number of cells (100,000 cells) resulted in a decreased PGCLC induction efficiency, likely due to an insufficient number of mRNAs distributed into each cell when a larger quantity was used. In contrast to human PGCLC induction (Kobayashi et al., 2017), addition of *BLIMP1* mRNA to *SOX17* mRNA did not cause positive effects on marmoset PGCLC induction (Figure S2C). Therefore, *SOX17* mRNA was only used together with the determined conditions (2 successive days of transfection and 50,000 cells for transfection) for PGCLC generation in this study.

Single-cell analysis of iPSCs and PGCLCs

To examine whether induced cells showed gene expression typical of PGCs, single-cell RNA sequencing (scRNA-seq) libraries were constructed from iPSCs (mRNA iPSCs) and d4_PGCLC aggregates (derived from mRNA iPSCs) (Table S1). The *SOX2*-positive iPSC population constituted ~80% of cells in the iPSC library, and the *NANOS3*-positive PGCLC population constituted 70%–80% of cells in the PGCLC library (Figures S3A and S3B). Principal-component analysis (PCA) was conducted by assembling all cells from both libraries. This analysis clearly separated the iPSC and PGCLC populations (Figure 2A), which were located on opposite sides of PC1. Well-characterized iPSC (*SOX2* and *ZIC2*) and PGC markers (*SOX17*, *NANOS3*, *KIT*, and *DND1*) were identified in the genes contributing to PC1 (Figures 2B and 2C). PC2 separated iPSC/PGCLC from other cell types, and iPSCs and PGCLCs were located on the upper part of the PC2 (Figure 2B). In the middle and lower parts of PC2, cells expressing well-known marker genes for somatic lineages (e.g., *NODAL*, *LHX1*, *HAND1*, *GABRP*, and *HOX* genes) were found (Figures 2B, 2C, and S4A).

To characterize cells that are present in our PGCLC aggregate, we aligned our dataset to a published dataset of PGCLC aggregates (Chen et al., 2019), which has been reported to include several cell types other than PGCLCs. Based on marker gene expressions reported in the studies of human and marmoset embryos (Bergmann et al., 2022; Tyser et al., 2021), four major populations of cells were identified in both marmoset and human PGCLC aggregates (Figures 2D and 2E): PGCLC (*SOX17* and *NANOS3*), amnion-like cells (AmLCs) (*HAND1*, *TFA2PC*, and *GABRP*), mesoderm-like cells (MeLCs) (*TBXT*, *EOMES*, *HAND1*, and *PDGFRA*), and a small number of definitive endoderm-like cells (DELCs) (*SOX17*, *OTX2*, and *FOXA2*). *SOX17* expression was, like humans, observed in DELCs as well as in PGCLCs; however, DELCs seemed to be already present in iPSCs rather than be newly induced. The rate of PGCLCs in our PGCLC aggregate was again estimated to be 70%–80% (Figure 2F). In marmoset PGCLCs, co-expression of *SOX17* and other germ cell marker genes (*BLIMP1/NANOS3/TFAP2C*) was observed (Figure S4B), suggesting conservation of the core PGC network. At the same time, we observed some differences in key transcription factor expression between marmoset and human PGCLCs: *POU5F1* was downregulated, while *NANOG* was upregulated in marmoset PGCLCs (Figure S4C). In somatic cells in the aggregates, *POU5F1* expression, although weak, was also observed (Figures 2B and 2E). In human systems (Castillo-Venzor et al., 2022), *POU5F1* expression is downregulated in other lineages, including DELCs, MeLCs, and AmLCs, although this downregulation itself is gradual. Therefore, some *POU5F1* may also persist early after their specification in marmosets. *NANOG*, a well-known iPSC and PGCLC marker, was expressed specifically in these cells, while *SOX2* is downregulated in both PGCLCs and somatic lineages (Figures 2B and 2E).

Single-cell analysis of developing gonads

Before setting out to advance the development of PGCLCs, we collected information on *in vivo* developing germ cells as a reference. We thus prepared scRNA-seq libraries from developing marmoset gonads. Ovaries from embryonic day 74 (E74), E82, and newborn marmosets and testes from E74, E87, 22 day olds (22d), and 3-year- and 10-month-old marmosets were subjected to single-cell analyses (Table S1). After tSNE plotting, the germ cells of interest were extracted

(C) Well-characterized iPSC markers (*SOX2* and *ZIC2*) and PGC markers (*SOX17*, *NANOS3*, *KIT*, and *DND1*) were found in the genes contributing to PC1.

(D) Alignment to human dataset suggests four key lineages in our aggregate: PGCLCs, mesoderm-like cells (MeLCs), amnion-like cells (AmLC), and definitive endoderm-like cells (DELCs).

(E) These subpopulations express appropriate markers identified in human systems (Tyser et al., 2021) or marmoset *in vivo* embryos (Bergmann et al., 2022).

(F) The relative proportion of each cell type in PGCLC aggregates, which shows an approximately 70%–80% efficiency of PGCLC induction in our approach.

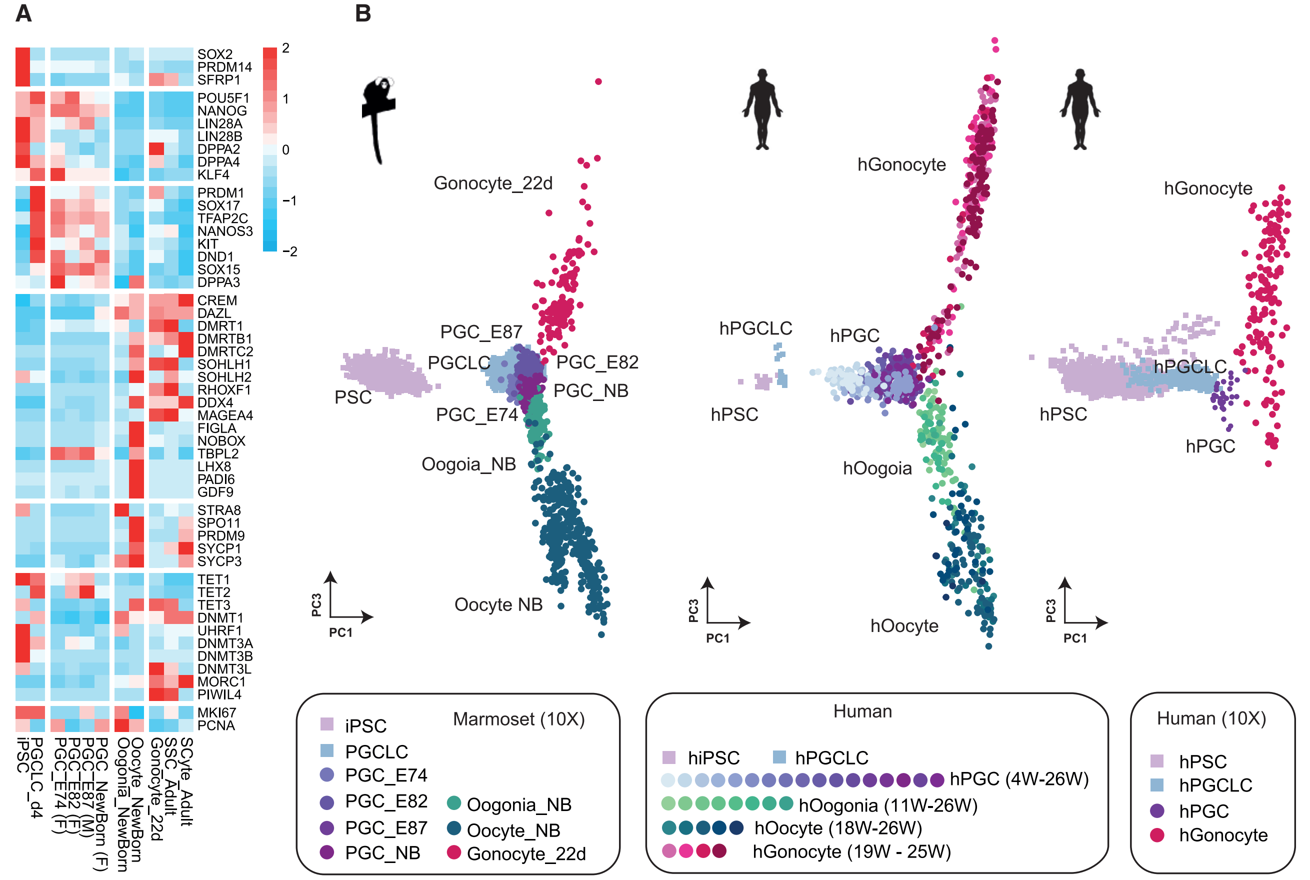

Figure 3. Gene expression analyses of PGCLCs and developmental germ cells

(A) Heatmap analyses of marker genes. iPSCs, PGCLCs, and marmoset endogenous developmental germ cells were examined. For expression levels in these cell types, cells expressing iPSC or germ cell markers were extracted from each library (Figure S3). Relative expression levels were shown (n = 1).

(B) PCA of PSCs, PGCLCs, and developing germ cells from and marmosets (left) and humans (center and right). Published human data were downloaded (Chen et al., 2019; Kojima et al., 2017; Li et al., 2017; Sohni et al., 2019) and analyzed together with marmoset data. Human data are shown in two separate panels according to the scRNA-seq platform. PSCs and germ cells were extracted from each 10x library based on marker gene expression. The anchoring function was used to integrate datasets of different platforms.

from each library (Figure S3). PGCs marked by *POU5F1* expression were extracted from fetal ovaries (E74 and E82) and testes (E87) (Figure S3). In E74 fetal testes, only a small number of PGCs were present, and they did not form a distinct cluster (Figure S3). Therefore, we did not extract the PGCs from this library. The body size of the E74 male was apparently smaller than that of its sibling E74 female. The small number of PGCs in E74 testes is, therefore, probably because many PGCs are still migrating and have not yet arrived at this stage (Aeckerle et al., 2015). In the somatic cells of E74 testes and ovaries, the expression of genes involved in initial sex differentiation was observed (*SRY*, *SOX9*, and *AMH* in testes, and *FOXL2* in ovaries) (Figure S5A), suggesting that sex differentiation is already initiated. Consistent with a previous report (Fereydouni et al., 2014), a wide range of cells (PGCs, oogonia, and early oocytes) were found in newborn ovaries (Figure S3). In 22d testes, germ cells were already differentiated into gonocytes, as shown by the decreased expression of *POU5F1* and elevated expression of *DDX4*. These expression patterns in 22d testis germ cells align with previously published studies (Albert et al., 2010; McKinnell et al., 2013; Mitchell et al., 2008).

PGCLCs align with *in vivo* PGCs

To examine whether our induced PGCLCs showed similar gene expression patterns to *in vivo* PGCs, we compared the expression of representative marker genes between *in vivo* germ cells and our PGCLCs (Figure 3A). A similar pattern was observed between *in vivo* PGCs and PGCLCs for the key marker genes. Both *in vivo* PGCs and PGCLCs showed the expression common marker genes for PSCs and PGCs (e.g., *POU5F1*, *NANOG*, *LIN28A*, *DPPA4*, and

KLF4) and PGC marker genes (*PRDM1*, *SOX17*, *TFAP2C*, *NANOS3*, *KIT*, *DND1*, and *SOX15*). However, none of the PSC marker genes (e.g., *SOX2*), gonocyte/oogonium genes (e.g., *DAZL* and *SOHLH1*), oocyte genes (e.g., *FIGLA* and *NOBOX*), or meiotic genes (e.g., *STRA8* and *SPO11*) were expressed in both *in vivo* PGCs and PGCLCs. Some differences in gene expression levels were observed between *in vivo* PGCs and PGCLCs. For example, *TBPL2* was upregulated in both male and female *in vivo* PGCs compared with PGCLCs (Figure 3A). This difference may be, at least in part, explained by the difference in developmental stages between *in vivo* PGCs (late PGCs) and PGCLCs (early PGCs).

To obtain further evidence that our PGCLCs were indeed PGC-like, PCA was conducted using marmoset iPSCs, PGCLCs, and *in vivo* germ cells (PGCs, gonocytes, oogonia, and oocytes). As expected, marmoset PGCLCs clustered together with *in vivo* PGCs (Figure 3B, right panel). Furthermore, we added human data to this analysis (Chen et al., 2019; Kojima et al., 2017; Li et al., 2017; Sohni et al., 2019) to obtain additional supporting evidence. Human PSCs, PGCLCs, and germ cells from various developmental stages aligned well with their corresponding cells in marmosets (Figure 3B). These results suggest that human and marmoset early germ cell development is overall conserved and that our PGCLCs were indeed reminiscent of *in vivo* PGCs.

Generation of xrtestes in mouse kidneys

To advance the development of marmoset PGCLCs, we used the xrtestis system that has been used to differentiate human PGCLCs (Hwang et al., 2020). However, instead of the *in vitro* air-liquid interface culture used in humans (Hwang et al., 2020; Yamashiro et al., 2018), we employed mouse kidney transplantation, as this system develops mouse reconstituted testes well (Matoba and Ogura, 2011). To recapitulate the male *in vivo* developmental process, we used two male iPSC lines harboring the ST and CE transgenes (971-STCE and 972-STCE) (Figure S1). To prepare xrtestes, FACS-purified d4_PGCLCs were mixed with E13.5 testis somatic cells for floating aggregate culture. The next day, the aggregates were transplanted into kidney capsules (Figure 4A).

Ten days after transplantation (day 10), the transplanted aggregates formed a testicular cord-like structure (Figures 4B–4D). xrtestes were successfully formed in most cases (the results of transplantation experiments are listed in Table S2). This structure was maintained for over 100 days (Figures 4C and 4D) unless cancer developed. In the xrtestes, PGCLC-derived cells (EGFP- and tdTomato-positive) and Sertoli cells (*WT1*-positive) were found within the cord structure (marked by *LAMININ*). In contrast, cells expressing the Leydig cell marker *HSDB* were found in interstitial regions (Figure 4C). Only a small number of PGCLC-derived cells was observed in the xrtestes until day 30 after transplantation. However, by day 30, the number of PGCLC-derived cells dramatically increased, occupying the entire circumference of each tubule (Figure 4D). Consistent with this massive increase in cell number, *MKI67* signals were observed in many PGCLC-derived cells from day 30 xrtestes (d30_xrtestes) (Figure 4E). Thus, PGCLCs are incorporated into the tubules of the xrtestes, and these PGCLC-derived cells actively proliferate within the tubules.

Differentiation of PGCLCs in xrtestes

Human PGCLCs develop into gonocyte-like cells over ~80 days (Hwang et al., 2020). We examined the expression of four well-characterized developmentally regulated marker genes (*TFAP2C*, *DDX4*, *MAGEA4*, and *PIWIL4*) in the xrtestes from several developmental points. Essentially similar results were obtained between the two different iPSC lines, 972-STCE and 971-STCE (Figures 5 and S6). *TFAP2C* (PGC marker) expression was observed in all PGCLC-derived cells before day 40 (Figures 5A and S6A). Subsequently, the number of PGCLC-derived cells expressing *TFAP2C* decreased dramatically. On day 81, none of the cells expressed this gene. No cells expressed gonocyte markers (*DDX4*, *MAGEA4*, and *PIWIL4*) on day 14. Almost all PGCLC-derived cells showed *DDX4* expression on day 28 (Figures 5B and S6A). On day 28, the expression of *MAGEA4* was also detected in only a small number of cells. As development progressed, the proportion of cells expressing *MAGEA4* increased, and all PGCLC-derived cells exhibited expression on days 81 and 104 (Figures 5C and S6A). *PIWIL4* was first, although weakly, observed in a small number of cells on days 81 and 104 (Figures 5D and S6A). Thus, these analyses revealed stepwise (in)activation of gonocyte (PGC) markers during xrtestis development, and the *in vivo* developmental pattern was recapitulated in our xrtestis system (Albert et al., 2010; Mitchell et al., 2008). qRT-PCR analyses also revealed the upregulation of these and some other gonocyte-expressed genes (*CREM*, *DMRT1*, *DMRT1B*, *DAZL*, *ZBTB16*, *FOXR1*, *RHOXF1*, *SOHLH1*, *SOHLH2*, and *RBM46*) in d56_, d81_, and d104_xrtestes (Figure S7). When d12_PGCLCs were used instead of d4_PGCLCs, *MAGEA4* expression was observed on day 43 after the transplantation (Figure S6B).

Demethylation of PGCLCs in xenoreconstituted testes

PGC development is accompanied by progressive loss of DNA methylation (Shirane et al., 2016). To determine DNA methylation status, we conducted single-cell bisulfite sequencing (scBS-seq) analyses in PGCLCs and PGCLC-derived cells in the xrtestes (Table S1 and S3). Simultaneously, RNA expression was analyzed in the same single cells using the cytoplasmic fraction. In d4_PGCLCs, the average DNA methylation level was 61.1% (Figure 6A). Interestingly, the level decreased to 45.7% in d12_PGCLCs, suggesting the occurrence of demethylation during long floating aggregate culture. The xrtestes were generated

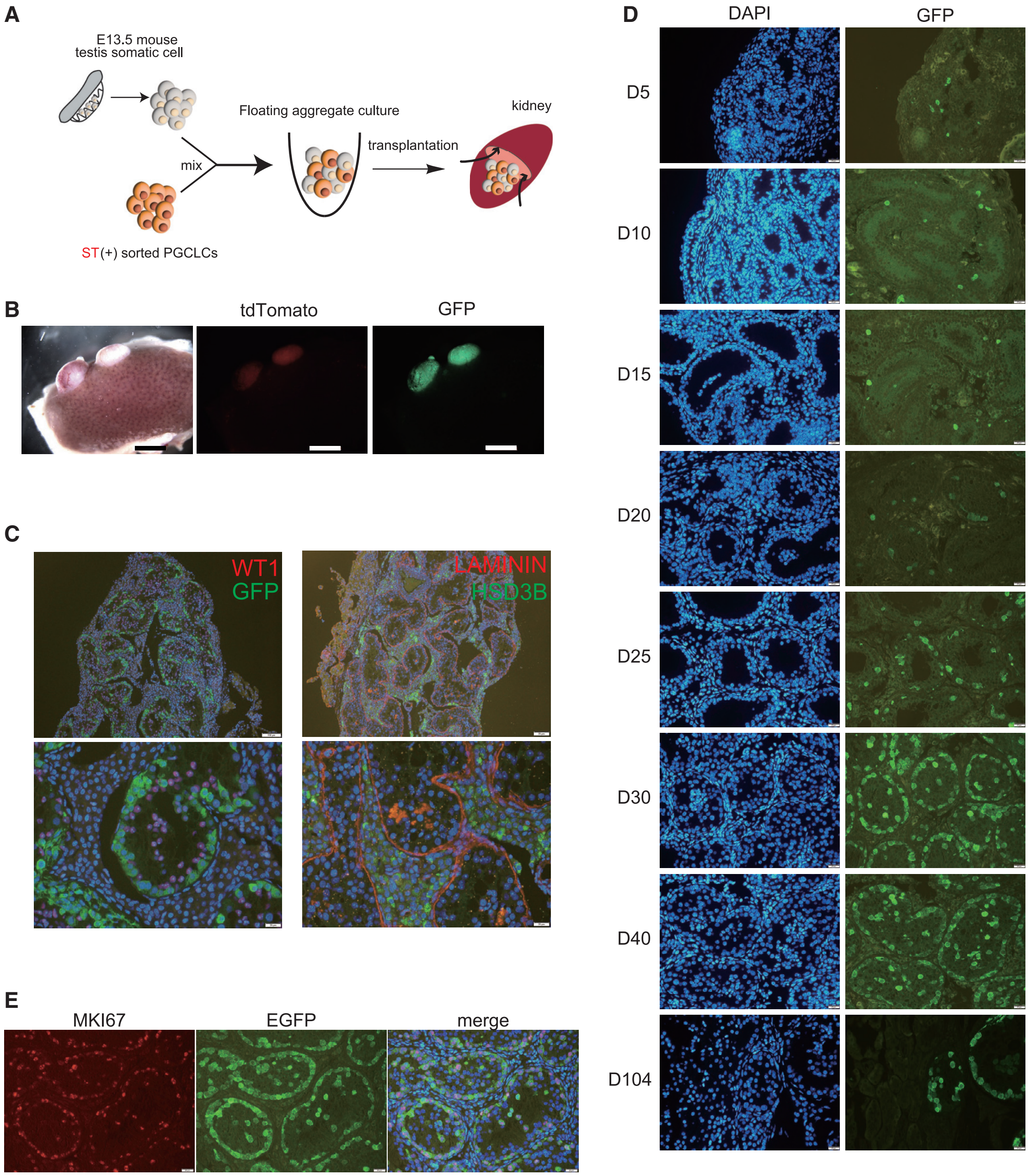

Figure 4. Proliferation of PGCLCs in xrtestis formed in mouse kidneys
(A) Scheme for differentiation of PGCLCs.
(B) d28_xrtestes formed under the kidney capsule. PGCLC (972-STCE)-derived cells express GFP and tdTomato. PGCLCs in xrtestes showed weaker tdTomato fluorescence than d4_PGCLCs. Scale bar, 2 mm.
(C) Reconstitution of testis structure (d104_xrtestes). Immunohistochemical analyses of the transplanted tissues. Markers: *LAMININ* (basement membrane), *HSD3B* (Leydig cells), and *WT1* (Sertoli cells). PGCLC (971-STCE)-derived cells express GFP. Scale bars, 100 μm (top) and 20 μm (bottom). Testis structure was also confirmed for xrtestes using 972-STCE.
(D) Time course change in the number of PGCLC (971-STCE)-derived cells during xrtestis development. Days after transplantation are indicated on the left side of the pictures. Scale bar, 20 μm. The same trend was observed in 972-STCE.
(E) Many PGCLC (971-STCE)-derived cells express *MKI67* in d30_xrtestes. Scale bar, 20 μm. Similar observations were made for 972-STCE.

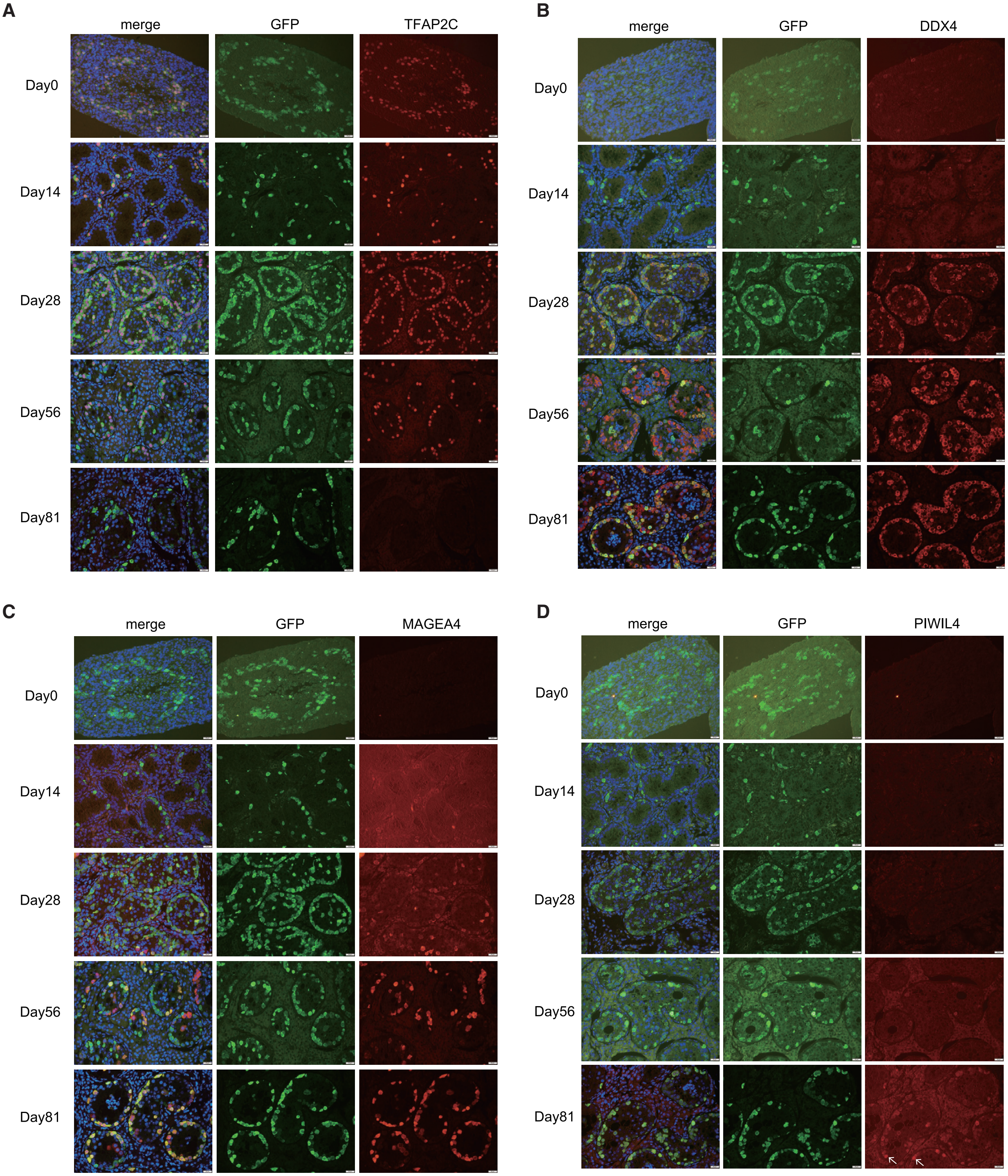

Figure 5. Differentiation of marmoset PGCLCs into gonocyte-like cells

(A–D) Immunofluorescence analyses of marker gene expression in developing xrtestes (972-STCE) and d5_PGCLC aggregates. *TFAP2C* (A), *DDX4* (B), *MAGEA4* (C), and *PIWIL4* (D). White arrows indicate nuclear *PIWIL4* staining. The staining patterns of *PIWIL4* were different from those of cytoplasmic EGFP staining. Scale bar, 20 μm. The results of all xrtestis experiments are summarized in Table S2.

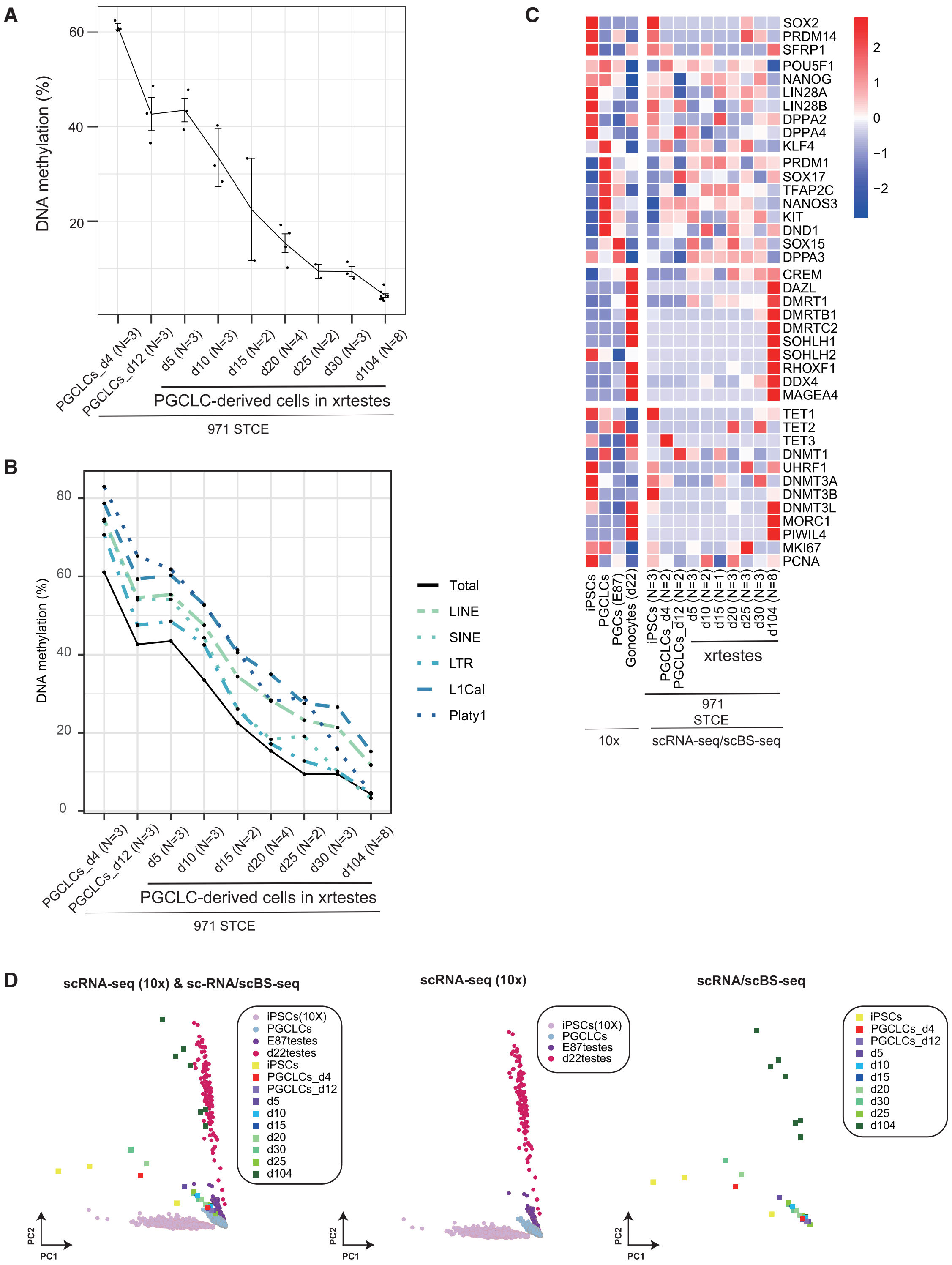

Figure 6. DNA methylation analyses during germ cell development from PGCLCs

(A) Single-cell analysis of DNA methylation during PGCLC development. PGCLCs_d4 and PGCLCs_d12 correspond to 4 and 12 days of floating aggregate culture, respectively. d5–d104 represent duration (days) after transplantation into mouse kidneys. Each dot represents a single cell. Cells examined in each stage were derived from xrtestes grown in one host mouse.

(B) Developmental dynamics of DNA methylation levels of retrotransposons. The average values are shown.

(legend continued on next page)

using d4_PGCLCs. DNA methylation levels decreased gradually in xrtestes (Figure 6A). Although we still detected some residual methylation in d30_xrtestes (9.4%), this level is close to the minimum level in d104_xrtestes (4.3%). Thus, DNA demethylation was recapitulated in our xrtestis system. However, the establishment of DNA methylation was likely not initiated, even in d104_xrtestes.

DNA methylation plays a critical role in the repression of retrotransposons in germline cells. In both mice and humans, active and young retrotransposons (e.g., IAP and LINE1 in mice, Alu and LINE1 in humans) show relatively high levels of residual DNA methylation in demethylated PGC genomes (Guo et al., 2015; Kobayashi et al., 2013; Seisenberger et al., 2012). Two types of potentially active retrotransposons exist in the marmoset genome. One is the LINE1 element, and the other is a very short ~100-bp SINE element named Platy-1 (Konkel et al., 2016). The DNA methylation dynamics of these two active retrotransposons (LINE1 and Platy-1) and three major classes of retrotransposons sequences (LINE, LTR, and SINE) were examined. In d4_PGCLCs, all three major retrotransposon sequences (LINE, 76.6%; LTR, 70.6%; SINE, 74.1%) showed higher levels than the genome average (61.1%), and the two active retrotransposons (LINE1: 78.7%, Platy-1: 82.9%) showed the highest levels (Figure 6B). As PGCLC development progressed, all retrotransposons lost DNA methylation with dynamics similar to those of the genomic average. In d104_xrtestes, DNA methylation levels of all the examined retrotransposons were decreased more than 5-fold (LINE1, 15.2% from 76.6%) to 17-fold (Platy-1, 4.7% from 82.9%). LINE1 (15.2%) and LINE (11.8%) still showed much higher levels than the genomic average (4.3%). On the other hands, other retrotransposons (LTR, 4.5%; SINE, 3.3%; Platy-1, 4.7%) showed similar levels to the genomic average (Figure 6B). Thus, a higher level of residual methylation was retained in LINE1 but not in Platy-1.

Comparison of germ cell development *in vivo* and in xrtestes

To correlate germ cell development in xrtestes with *in vivo* germ cell development, we analyzed the RNA expression of PGCLC-derived germ cells, in which we analyzed DNA methylation. Upon differentiation of iPSCs into PGCLCs, the expression of *UHRF1* (involved in DNA methylation maintenance) and *de novo* DNA methyltransferase *DNMT3A/3B/3L* decreased (Figure 6C). This decrease may be involved in the demethylation of the PGCLC genome. At the initial stage of xrtestis development (d5 – d30), pluripotent genes (*POU5F1*, *NANOG*, *LIN28A*, and *KLF4*) and PGC genes (*PRDM1*, *SOX17*, *TFAP2C*, *NANOS3*, *KIT*, *DND1*, *SOX15*, and *DPPA3*) were expressed. Their expression was decreased in d104_xrtestes. Instead, *DNMT3L*, *PIWIL4*, and *MORC1* were highly upregulated, establishing the stage for *de novo* methylation of retrotransposons. In addition, the expression of gonocyte genes (*CREM*, *DAZL*, *DMRT1*, *DMRTB1*, *DMRTC2*, *SOHLH1*, *SOHLH2*, *RHOXF1*, *DDX4*, and *MAGEA4*) was observed in d104_xrtestes as well as in gonocytes from *in vivo* 22d testes (Figure 6C). Some of them (*CREM*, *DMRT1*, and *DDX4*) were expressed from earlier stages of xrtestis development (Figures 6C and 5B for *DDX4*). These genes were expressed, although very weakly, in *in vivo* PGCs from E87 testes (Figure 6C) or E90 testes (see Figure 7 for *DDX4*), suggesting that developmentally regulated gene expression is recapitulated in the xrtestis system. PCA revealed that PGCLC-derived cells, except those from d104_xrtestes, closely aligned with E87 testis germ cells (Figure 6D). On the other hand, PGCLC-derived germ cells from d104_xrtestes clustered together with 22d testis germ cells. Thus, PGCLCs differentiate into gonocyte-like cells in the xrtestis.

To more precisely correlate PGCLC-derived cell development with *in vivo* germ cell development, immunohistochemichal analyses (TFAP2C, DDX4, MAGEA4, and PIWIL4) were conducted using testes from several developmental stages (E85, E90, E122, 2-day, 33-day, 3-month, 4-month, and 5-month testes) (Figure 7). In E85 and E90 testes, many germ cells co-expressed *TFAP2C* and *DDX4*, although a small number of cells expressed only *TFAP2C* (Figure 7B and data not shown). In E122 testes, many germ cells still co-expressed *TFAP2C* and *DDX4*, and *MAGEA4* expression was started in a small part of germ cells (similar to day 28, 30, and 40 xrtestes) (Figure 7C). In 2-day testes, many germ cells expressed *MAGEA4*, which is similar to day 56 xrtestes. Almost all (none) of germ cells express *MAGEA4* (*TFAP2C*) in 33-day testes. *PIWIL4* expression, which was weakly observed in day 81 and 104 xrtestes, was first observable in 3-month testes (Figures 5D, 7D, 7E, and S6A).

DISCUSSION

In this study, marmoset PGCLCs were generated from iPSCs using an mRNA-transfection-based method. This is the first report of PGCLC generation using mRNAs. Since

(C) Heatmap analyses of marker genes. Relative expression levels were shown. Apparently variable expression of pluripotent genes during xrtestis development is likely, at least in part, due to technical issues (small number of single cells, medium expression levels, and multiple stages analyzed.

(D) The data obtained by 10x and scRNA-seq/scBS-seq are shown together (left) or separately (center and right). The anchoring function was not used to integrate datasets of different platforms. There is a small displacement between the iPSCs of the 10x platform and iPSCs of the scRNA-seq/scBS-seq platform.

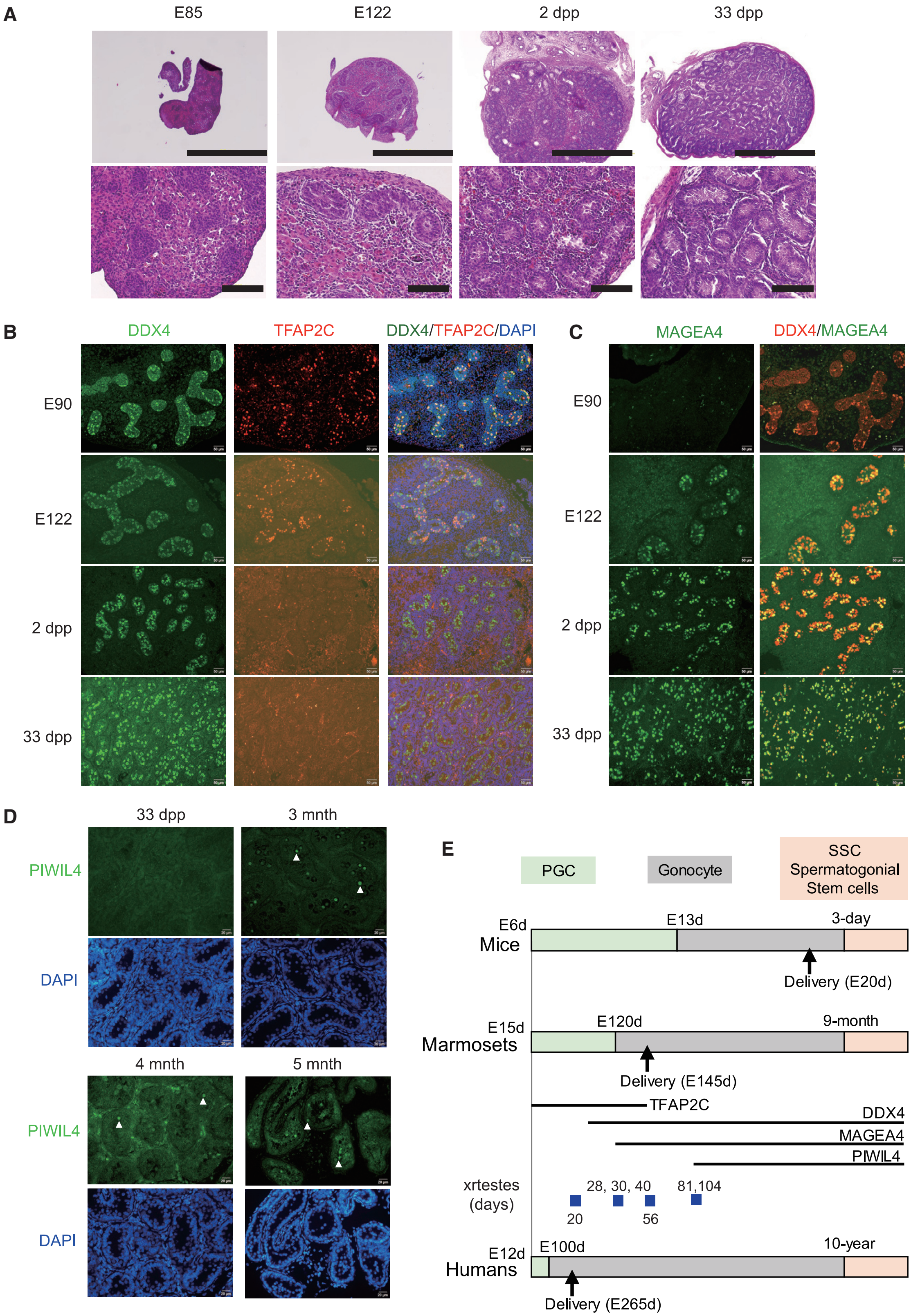

(legend on next page)

this method is simple and efficient, it may also be useful for other species. Furthermore, the generated PGCLCs differentiated into gonocyte-like cells in the xrtestes that were transplanted under the kidney capsules of immunodeficient mice. Stepwise expression of PGC and gonocyte marker genes was observed. DNA methylation was progressively lost and almost completely erased in the gonocyte-like cells. Thus, early germ cell development *in vivo* was recapitulated by our PGCLC-initiated system. This study provides a platform for developmental studies on marmoset germ cells and the generation of genetically modified marmosets.

We induced PGCLCs from iPSCs using a combination of *SOX17* mRNA transfection and subsequent floating aggregate culture. Our method was based on a report on PGCLC generation by *SOX17* overexpression using an inducible system (Irie et al., 2015; Kobayashi et al., 2017), which requires prior transgene integration. To omit this step, we used mRNA transfection-based overexpression. Although the induction rate was highly dependent on the iPSC lines (data not shown), as in humans (Chen et al., 2017), our induction efficiency usually reached >80% when highly competent lines were used (Figures 1B and S1). Since this efficiency is comparable with or higher than those of existing methods (Irie et al., 2015; Jo et al., 2022; Kobayashi et al., 2017; Sakai et al., 2020; Sasaki et al., 2015; Sosa et al., 2018; Yoshimatsu et al., 2021), we believe that the mRNA transfection method reported here serves as an alternative method.

After developing a solid foundation for PGCLC induction, we aimed to differentiate PGCLCs into a more advanced state. Matoba and Ogura (2011) reported that *in vivo* mouse PGCs developed into spermatids in reconstituted testes transplanted under the kidney capsule. This led us to examine whether immunodeficient mouse kidneys serve as suitable sites to develop xrtestes. The xrtestes developed well under the kidney capsule. Using this technique, PGC LCs in the xrtestes were found to differentiate into gonocyte-like cells. All PGCLC-derived cells from the d81_xrtestes were negative for the PGC marker *TFAP2C* (Figure 5). Given that a small number of the germ cells still express *TFAP2C* in newborn testes (Figure 7B), gonocyte-like cells in d81_ xrtestes are more developmentally advanced than newborn testis germ cells. However, our bisulfite sequencing analyses showed that PGCLC-derived cells in d81_ and d104_xrtestes did not undergo *de novo* DNA methylation (Figure 6). In marmoset testes, *de novo* DNA methylation is initiated at 4 months at the latest (Langenstroth-Rower et al., 2017), although the precise timing has not yet been determined. Therefore, gonocyte-like cells in d81_ and d104_xrtestes likely correspond to *in vivo* gonocytes between newborn and 4-month-old animals. *PIWIL4* expression was first observable in 3-month testes, but not in 2-month testes (Figures 7D and 7E). In d81_ and d104_xrtestes, weak *PIWIL4* expression was observed. Thus, these xrtestes likely correspond to 2- or 3-month testes *in vivo*. The kidney transplantation method reported here will be a robust *in vivo* method for male PGCLC differentiation in other species as well. Reconstituted embryonic ovaries from mice and cynomolgus monkeys develop well in mouse kidneys (Matoba and Ogura, 2011; Mizuta et al., 2022). Therefore, the kidney transplantation of (xeno)reconstituted ovaries may be also useful for advancement of female PGCLC development in marmosets and other species.

Successful marmoset PGCLC induction has been reported previously by Yoshimatsu et al. (2021). However, their induction efficiency (~40% for the two ESC lines and 1%–2% for the two iPSC lines) seemed to be not as high as ours. In addition, their study did not test the developmental potential of these PGCLCs. By contrast, PGCLCs were differentiated into gonocyte-like cells in our study. Their methods involved both transgene (*SOX17* and *BLIMP1*) overexpression and pre-ME/iMeLC induction steps, and it took 10 days (+prior transgene integration) for the procedure. Our method required only 6 days, and no prior transgene integration is required. Furthermore, we did not use *BLIMP1*, because the addition of *BLIMP1* mRNAs to *SOX17* mRNAs did not have any positive effect on PGCLC induction in our system (Figure S2C). Combinatorial expression of *BLIMP1* and *SOX17* has been reported to promote PGCLCs in humans (Kobayashi et al., 2017). Species difference or different methods used likely account for the discrepancy of the effect of *BLIMP1*. Recently, another group reported marmoset PGCLC induction from PSCs (Seita et al., 2022). Their induction efficiency was 40% at the highest using a similar method reported in cynomolgus monkeys and rabbits (Kobayashi et al., 2021; Sakai et al., 2020). They cultured PSCs in the presence of the WNT inhibitor IWR-1, and PGCLCs were directly induced from PSCs without undergoing pre-ME/iMeLC. Although their PGCLCs differentiated into *DDX4*-positive cells (corresponding to late PGCs or gonocytes), complete DNA

Figure 7. *In vivo* germ cell development in marmoset testes
(A) Histology of developing testes. Scale bars, 2 mm (top) and 200 μm (bottom). n = 1.
(B–D) Immunohistochemical analyses of the developing testes. Germ cell markers: *DDX4/TFAP2C* (B), *DDX4/MAGEA4* (C), and *PIWIL4* (D). Arrow heads in (D) indicate *PIWIL4*-positive cells. Scale bars, 20 μm. n = 1.
(E) Schematic diagram for germ cell development in mice, marmosets, and humans. The expression windows of *TFAP2C*, *DDX4*, *MAGEA4*, and *PIWIL4* in marmosets are shown. PGC to gonocyte transition was determined based on the disappearance of *TFAP2C* expression and the start of *MAGEA4* expression. Possible corresponding stages of xrtestis development are shown.

demethylation and the potential for differentiation into *MAGEA4*-positive gonocyte-like cells were not examined. Thus, our study provides two efficient and useful systems associated with marmoset PGCLCs: (1) an mRNA-transfection-based PGCLC induction system and (2) a kidney transplantation-based PGCLC to gonocyte-like cell differentiation system.

In contrast to the PSCs cultured in the presence of the WNT inhibitor IWR-1 (Seita et al., 2022), our iPSCs cultured in MEF-conditioned medium did not directly differentiate into PGC lineage upon aggregate formation. This difference in germline competency may be due to differences in culture conditions and/or cell lines. We speculate that *SOX17* mRNA overexpression makes iPSC transcriptome closer to PGC, and these iPSCs become preferentially differentiating into PGC lineage upon aggregate formation. Given that mRNA transfection only can induce PGCLCs (Figure 1B), cytokines are not essential for PGCLC induction in our system. However, omitting growth factors slightly reduced the number of PGCLCs induced. At least, SCF is required for the maintenance of PGCLCs. Further studies are needed to determine other cytokines used here play any positive roles in the *SOX17* mRNA-mediated induction of PGCLCs.

Long-term *SOX17* overexpression in human ESCs have been reported to give competency for differentiation into DE lineage (Seguin et al., 2008). In contrast, our short-term overexpression in marmoset iPSCs and aggregate formation resulted in exclusive differentiation into PGC lineage (Figures 2D and 2E). What is the reason for this difference? One possibility is the duration of *SOX17* overexpression. Consistent with this, we observed decreased efficiency of PGCLC induction by increasing the mRNA transfection period (Figure S2A). In addition, aggregate culture conditions may promote differentiation into PGC lineage. Another possibility is species-specific difference. Marmoset primed PSCs are suggested to be slightly earlier developmental stage than human primed PSCs (Bergmann et al., 2022). Interestingly, a recent study suggested that *SOX17* binding to PGC and DE promoter/enhancer was determined by the cofactors (PGC cofactors: *POU5F1*, *NANOG*, and *TFAP2C*; DE cofactors: *EOMES*, *SMAD2/3/4*, *FOXA1/A2*, and *ZIC2/3/5*) (Tang et al., 2022). The earlier state of marmoset PSCs, therefore, might promote directing *SOX17* to PGC promoter/enhancer in preference to DE promoter/enhancer.

For the generation of functional gametes, the gonocyte-like cells generated in this study require further development. The next step is further differentiation into late gonocyte-like cells that undergo *de novo* DNA methylation. It is important to understand the cues that initiate *de novo* DNA methylation. Furthermore, the current protocol requires a long time to differentiate gonocyte-like cells from PGCLCs. Shortening this time is also an important next step. However, undertaking the normal demethylation process in PGCs is likely important for generating functional gametes. In fact, bypassing this resulted in abnormal DNA methylation patterns in mouse oocytes (Hamazaki et al., 2021). Furthermore, *in vitro* PGCLC culture and the resultant prior erasure of DNA methylation have been reported to be essential for the spermatogenic potential of PGCLC-derived spermatogonial stem cells (Ishikura et al., 2021). DNA methylation dynamics revealed in this study are, in part, useful for determining which developmental stage can be bypassed without affecting the DNA demethylation process. Our study provides a solid foundation for complete generation of gametes from pluripotent stem cells.

EXPERIMENTAL PROCEDURES

Resource availability

Corresponding author

Further information should be requested from the corresponding author, Toshiaki Watanabe (watanabe-tos@ncchd.go.jp).

Materials availability

Materials can be requested from the corresponding author.

Data and code availability

Single-cell data generated by 10x Genomics platform were deposited to ArrayExpress (accession no. E-MTAB-12123) and scRNA-seq/scBS-seq data were registered to DDBJ (accession nos. DRA014666 and DRA014672). Data and code will be shared with the research community upon request.

Cell culture

Marmoset iPSCs were cultured in MEF-conditioned primate ES cell medium (REPROCELL, RCHEMD001) containing bFGF (REPROCELL, RCHEOT003) and 1× antibiotic-antimycotic (Nacalai Tesque, 0289-54) (Watanabe et al., 2019). The methods for the induction of PGCLCs are found in the supplemental information.

Single-cell library generation and data analyses

The sample information is summarized in Table S1. Summary statistics for simultaneous scRNA-seq and scBS-seq analyses are found in Table S3. See supplemental information for the library generation and the analyses.

SUPPLEMENTAL INFORMATION

Supplemental information can be found online at https://doi.org/10.1016/j.stemcr.2023.08.006.

ACKNOWLEDGMENTS

We thank Tomomi Shimogori and Kita Yoshiaki for marmoset samples, Akihiro Umezawa and Hidenobu Soejima for their encouragement, Yasufumi Sakakibara and Kengo Sato for their support with computational resources, Fuchou Tang, Rui Wang, and Shin-ichi Tomizawa for single-cell bisulfite-seq protocols, Akiyumi Tashiro for making comments on our manuscript, Kenji Kawai for making paraffin blocks, Haruka Shinohara for advice on karyotyping, and veterinarians and animal technicians in CIEA for marmoset and

mouse housing. This research was supported by AMED and KAKENHI under the grant nos. JP19gm6310010, JP20gm6310010, JP21gm6310010, and JP22gm6310010 (AMED to T.W.), JP18dm 020765 (AMED to E.S.), 20H05764, 20H03177, and 22K18356 (KAK ENHI to T.W.).

AUTHOR CONTRIBUTIONS

M.K.-I., C.D., and T.W. performed the experiments. C.P., T.B., M.K.-I., and T.W. performed informatics analyses. M.K.-I. and T.W. conceived of the study, designed the experiments, and wrote the manuscript. K.K., K.S., Y.T., and K.W. provided materials and information. E.S. shared the equipment and samples. H.Y., H.K., A.M., K.K., M.K.-I., and T.W. generated sequencing libraries. All authors read and approved the final manuscripts.

DECLARATION OF INTERESTS

The authors declare competing interests.

Received: August 23, 2022
Revised: August 9, 2023
Accepted: August 10, 2023
Published: September 7, 2023

REFERENCES

Aeckerle, N., Drummer, C., Debowski, K., Viebahn, C., and Behr, R. (2015). Primordial germ cell development in the marmoset monkey as revealed by pluripotency factor expression: suggestion of a novel model of embryonic germ cell translocation. Mol. Hum. Reprod. *21*, 66–80. https://doi.org/10.1093/molehr/gau088.

Albert, S., Ehmcke, J., Wistuba, J., Eildermann, K., Behr, R., Schlatt, S., and Gromoll, J. (2010). Germ cell dynamics in the testis of the postnatal common marmoset monkey (Callithrix jacchus). Reproduction *140*, 733–742. https://doi.org/10.1530/REP-10-0235.

Bergmann, S., Penfold, C.A., Slatery, E., Siriwardena, D., Drummer, C., Clark, S., Strawbridge, S.E., Kishimoto, K., Vickers, A., Tewary, M., et al. (2022). Spatial profiling of early primate gastrulation in utero. Nature *609*, 136–143. https://doi.org/10.1038/s41586-022-04953-1.

Castillo-Venzor, A., Penfold, C.A., Morgan, M.D., Tang, W.W.C., Kobayashi, T., Wong, F.C.K., Bergmann, S., Slatery, E., Boroviak, T.E., Marioni, J.C., and Surani, M.A. (2022). Origin and segregation of the human germline. Preprint at bioRxiv. https://doi.org/10.1101/2022.07.06.498671.

Chen, D., Liu, W., Lukianchikov, A., Hancock, G.V., Zimmerman, J., Lowe, M.G., Kim, R., Galic, Z., Irie, N., Surani, M.A., et al. (2017). Germline competency of human embryonic stem cells depends on eomesodermin. Biol. Reprod. *97*, 850–861. https://doi.org/10.1093/biolre/iox138.

Chen, D., Sun, N., Hou, L., Kim, R., Faith, J., Aslanyan, M., Tao, Y., Zheng, Y., Fu, J., Liu, W., et al. (2019). Human primordial germ cells are specified from lineage-primed progenitors. Cell Rep. *29*, 4568–4582.e5. https://doi.org/10.1016/j.celrep.2019.11.083.

Fereydouni, B., Drummer, C., Aeckerle, N., Schlatt, S., and Behr, R. (2014). The neonatal marmoset monkey ovary is very primitive exhibiting many oogonia. Reproduction *148*, 237–247. https://doi.org/10.1530/REP-14-0068.

Guo, F., Yan, L., Guo, H., Li, L., Hu, B., Zhao, Y., Yong, J., Hu, Y., Wang, X., Wei, Y., et al. (2015). The transcriptome and DNA methylome landscapes of human primordial germ cells. Cell *161*, 1437–1452. https://doi.org/10.1016/j.cell.2015.05.015.

Hamazaki, N., Kyogoku, H., Araki, H., Miura, F., Horikawa, C., Hamada, N., Shimamoto, S., Hikabe, O., Nakashima, K., Kitajima, T.S., et al. (2021). Reconstitution of the oocyte transcriptional network with transcription factors. Nature *589*, 264–269. https://doi.org/10.1038/s41586-020-3027-9.

Hong, H., Takahashi, K., Ichisaka, T., Aoi, T., Kanagawa, O., Nakagawa, M., Okita, K., and Yamanaka, S. (2009). Suppression of induced pluripotent stem cell generation by the p53-p21 pathway. Nature *460*, 1132–1135. https://doi.org/10.1038/nature08235.

Hwang, Y.S., Suzuki, S., Seita, Y., Ito, J., Sakata, Y., Aso, H., Sato, K., Hermann, B.P., and Sasaki, K. (2020). Reconstitution of prospermatogonial specification in vitro from human induced pluripotent stem cells. Nat. Commun. *11*, 5656. https://doi.org/10.1038/s41467-020-19350-3.

Irie, N., Weinberger, L., Tang, W.W.C., Kobayashi, T., Viukov, S., Manor, Y.S., Dietmann, S., Hanna, J.H., and Surani, M.A. (2015). SOX17 is a critical specifier of human primordial germ cell fate. Cell *160*, 253–268. https://doi.org/10.1016/j.cell.2014.12.013.

Ishikura, Y., Ohta, H., Sato, T., Murase, Y., Yabuta, Y., Kojima, Y., Yamashiro, C., Nakamura, T., Yamamoto, T., Ogawa, T., and Saitou, M. (2021). In vitro reconstitution of the whole male germ-cell development from mouse pluripotent stem cells. Cell Stem Cell *28*, 2167–2179.e9. https://doi.org/10.1016/j.stem.2021.08.005.

Jo, K., Teague, S., Chen, B., Khan, H.A., Freeburne, E., Li, H., Li, B., Ran, R., Spence, J.R., and Heemskerk, I. (2022). Efficient differentiation of human primordial germ cells through geometric control reveals a key role for Nodal signaling. Elife *11*, e72811. https://doi.org/10.7554/eLife.72811.

Kobayashi, H., Sakurai, T., Miura, F., Imai, M., Mochiduki, K., Yanagisawa, E., Sakashita, A., Wakai, T., Suzuki, Y., Ito, T., et al. (2013). High-resolution DNA methylome analysis of primordial germ cells identifies gender-specific reprogramming in mice. Genome Res. *23*, 616–627. https://doi.org/10.1101/gr.148023.112.

Kobayashi, T., Castillo-Venzor, A., Penfold, C.A., Morgan, M., Mizuno, N., Tang, W.W.C., Osada, Y., Hirao, M., Yoshida, F., Sato, H., et al. (2021). Tracing the emergence of primordial germ cells from bilaminar disc rabbit embryos and pluripotent stem cells. Cell Rep. *37*, 109812. https://doi.org/10.1016/j.celrep.2021.109812.

Kobayashi, T., Zhang, H., Tang, W.W.C., Irie, N., Withey, S., Klisch, D., Sybirna, A., Dietmann, S., Contreras, D.A., Webb, R., et al. (2017). Principles of early human development and germ cell program from conserved model systems. Nature *546*, 416–420. https://doi.org/10.1038/nature22812.

Kojima, Y., Sasaki, K., Yokobayashi, S., Sakai, Y., Nakamura, T., Yabuta, Y., Nakaki, F., Nagaoka, S., Woltjen, K., Hotta, A., et al. (2017). Evolutionarily distinctive transcriptional and signaling programs drive human germ cell lineage specification from pluripotent stem cells. Cell Stem Cell *21*, 517–532.e5. https://doi.org/10.1016/j.stem.2017.09.005.

Konkel, M.K., Ullmer, B., Arceneaux, E.L., Sanampudi, S., Brantley, S.A., Hubley, R., Smit, A.F.A., and Batzer, M.A. (2016). Discovery of a new repeat family in the Callithrix jacchus genome. Genome Res. *26*, 649–659. https://doi.org/10.1101/gr.199075.115.

Langenstroth-Rower, D., Gromoll, J., Wistuba, J., Trondle, I., Laurentino, S., Schlatt, S., and Neuhaus, N. (2017). De novo methylation in male germ cells of the common marmoset monkey occurs during postnatal development and is maintained in vitro. Epigenetics *12*, 527–539. https://doi.org/10.1080/15592294.2016.1248007.

Li, L., Dong, J., Yan, L., Yong, J., Liu, X., Hu, Y., Fan, X., Wu, X., Guo, H., Wang, X., et al. (2017). Single-cell RNA-seq analysis maps development of human germline cells and gonadal niche interactions. Cell Stem Cell *20*, 858–873.e4. https://doi.org/10.1016/j.stem.2017.03.007.

Matoba, S., and Ogura, A. (2011). Generation of functional oocytes and spermatids from fetal primordial germ cells after ectopic transplantation in adult mice. Biol. Reprod. *84*, 631–638. https://doi.org/10.1095/biolreprod.110.087122.

McKinnell, C., Mitchell, R.T., Morris, K., Anderson, R.A., Kelnar, C.J.H., Wallace, W.H., and Sharpe, R.M. (2013). Perinatal germ cell development and differentiation in the male marmoset (Callithrix jacchus): similarities with the human and differences from the rat. Hum. Reprod. *28*, 886–896. https://doi.org/10.1093/humrep/des465.

Mitchell, R.T., Cowan, G., Morris, K.D., Anderson, R.A., Fraser, H.M., McKenzie, K.J., Wallace, W.H.B., Kelnar, C.J.H., Saunders, P.T.K., and Sharpe, R.M. (2008). Germ cell differentiation in the marmoset (Callithrix jacchus) during fetal and neonatal life closely parallels that in the human. Hum. Reprod. *23*, 2755–2765. https://doi.org/10.1093/humrep/den295.

Mizuta, K., Katou, Y., Nakakita, B., Kishine, A., Nosaka, Y., Saito, S., Iwatani, C., Tsuchiya, H., Kawamoto, I., Nakaya, M., et al. (2022). Ex vivo reconstitution of fetal oocyte development in humans and cynomolgus monkeys. EMBO J. *41*, e110815. https://doi.org/10.15252/embj.2022110815.

Park, J.E., and Sasaki, E. (2020). Assisted reproductive techniques and genetic manipulation in the common marmoset. ILAR J. *61*, 286–303. https://doi.org/10.1093/ilar/ilab002.

Phillips, I.R. (1976). The embryology of the common marmoset (Callithrix jacchus). Adv. Anat. Embryol. Cell Biol. *52*, 3–47.

Poleganov, M.A., Eminli, S., Beissert, T., Herz, S., Moon, J.I., Goldmann, J., Beyer, A., Heck, R., Burkhart, I., Barea Roldan, D., et al. (2015). Efficient reprogramming of human fibroblasts and blood-derived endothelial progenitor cells using nonmodified RNA for reprogramming and immune evasion. Hum. Gene Ther. *26*, 751–766. https://doi.org/10.1089/hum.2015.045.

Sakai, Y., Nakamura, T., Okamoto, I., Gyobu-Motani, S., Ohta, H., Yabuta, Y., Tsukiyama, T., Iwatani, C., Tsuchiya, H., Ema, M., et al. (2020). Induction of the germ cell fate from pluripotent stem cells in cynomolgus monkeysdagger. Biol. Reprod. *102*, 620–638. https://doi.org/10.1093/biolre/ioz205.

Sasaki, K., Yokobayashi, S., Nakamura, T., Okamoto, I., Yabuta, Y., Kurimoto, K., Ohta, H., Moritoki, Y., Iwatani, C., Tsuchiya, H., et al. (2015). Robust in vitro induction of human germ cell fate from pluripotent stem cells. Cell Stem Cell *17*, 178–194. https://doi.org/10.1016/j.stem.2015.06.014.

Seguin, C.A., Draper, J.S., Nagy, A., and Rossant, J. (2008). Establishment of endoderm progenitors by SOX transcription factor expression in human embryonic stem cells. Cell Stem Cell *3*, 182–195. https://doi.org/10.1016/j.stem.2008.06.018.

Seisenberger, S., Andrews, S., Krueger, F., Arand, J., Walter, J., Santos, F., Popp, C., Thienpont, B., Dean, W., and Reik, W. (2012). The dynamics of genome-wide DNA methylation reprogramming in mouse primordial germ cells. Mol. Cell *48*, 849–862. https://doi.org/10.1016/j.molcel.2012.11.001.

Seita, Y., Cheng, K., McCarrey, J.R., Yadu, N., Cheeseman, I., Bagwell, A., Ross, C.N., Santana-Toro, I., Yen, L.-H., Vargas, S., et al. (2022). Efficient generation of marmoset primordial germ cell-like cells using induced pluripotent stem cells. Preprint at bioRxiv. https://doi.org/10.1101/2022.07.25.501382.

Shirane, K., Kurimoto, K., Yabuta, Y., Yamaji, M., Satoh, J., Ito, S., Watanabe, A., Hayashi, K., Saitou, M., and Sasaki, H. (2016). Global landscape and regulatory principles of DNA methylation reprogramming for germ cell specification by mouse pluripotent stem cells. Dev. Cell *39*, 87–103. https://doi.org/10.1016/j.devcel.2016.08.008.

Sohni, A., Tan, K., Song, H.W., Burow, D., de Rooij, D.G., Laurent, L., Hsieh, T.C., Rabah, R., Hammoud, S.S., Vicini, E., and Wilkinson, M.F. (2019). The neonatal and adult human testis defined at the single-cell level. Cell Rep. *26*, 1501–1517.e4. https://doi.org/10.1016/j.celrep.2019.01.045.

Sosa, E., Chen, D., Rojas, E.J., Hennebold, J.D., Peters, K.A., Wu, Z., Lam, T.N., Mitchell, J.M., Sukhwani, M., Tailor, R.C., et al. (2018). Differentiation of primate primordial germ cell-like cells following transplantation into the adult gonadal niche. Nat. Commun. *9*, 5339. https://doi.org/10.1038/s41467-018-07740-7.

Tang, W.W.C., Castillo-Venzor, A., Gruhn, W.H., Kobayashi, T., Penfold, C.A., Morgan, M.D., Sun, D., Irie, N., and Surani, M.A. (2022). Sequential enhancer state remodelling defines human germline competence and specification. Nat. Cell Biol. *24*, 448–460. https://doi.org/10.1038/s41556-022-00878-z.

Tyser, R.C.V., Mahammadov, E., Nakanoh, S., Vallier, L., Scialdone, A., and Srinivas, S. (2021). Single-cell transcriptomic characterization of a gastrulating human embryo. Nature *600*, 285–289. https://doi.org/10.1038/s41586-021-04158-y.

Watanabe, T., Yamazaki, S., Yoneda, N., Shinohara, H., Tomioka, I., Higuchi, Y., Yagoto, M., Ema, M., Suemizu, H., Kawai, K., and Sasaki, E. (2019). Highly efficient induction of primate iPS cells by combining RNA transfection and chemical compounds. Gene Cell. *24*, 473–484. https://doi.org/10.1111/gtc.12702.

Yamashiro, C., Sasaki, K., Yabuta, Y., Kojima, Y., Nakamura, T., Okamoto, I., Yokobayashi, S., Murase, Y., Ishikura, Y., Shirane, K., et al. (2018). Generation of human oogonia from induced pluripotent stem cells in vitro. Science *362*, 356–360. https://doi.org/10.1126/science.aat1674.

Yoshimatsu, S., Nakajima, M., Iguchi, A., Sanosaka, T., Sato, T., Nakamura, M., Nakajima, R., Arai, E., Ishikawa, M., Imaizumi, K., et al. (2021). Non-viral induction of transgene-free iPSCs from somatic fibroblasts of multiple mammalian species. Stem Cell Rep. *16*, 754–770. https://doi.org/10.1016/j.stemcr.2021.03.002.

Stem Cell Reports, Volume *18*

Supplemental Information

mRNA-based generation of marmoset PGCLCs capable of differentiation into gonocyte-like cells

Musashi Kubiura-Ichimaru, Christopher Penfold, Kazuaki Kojima, Constance Dollet, Haruka Yabukami, Katsunori Semi, Yasuhiro Takashima, Thorsten Boroviak, Hideya Kawaji, Knut Woltjen, Aki Minoda, Erika Sasaki, and Toshiaki Watanabe

Figure S1

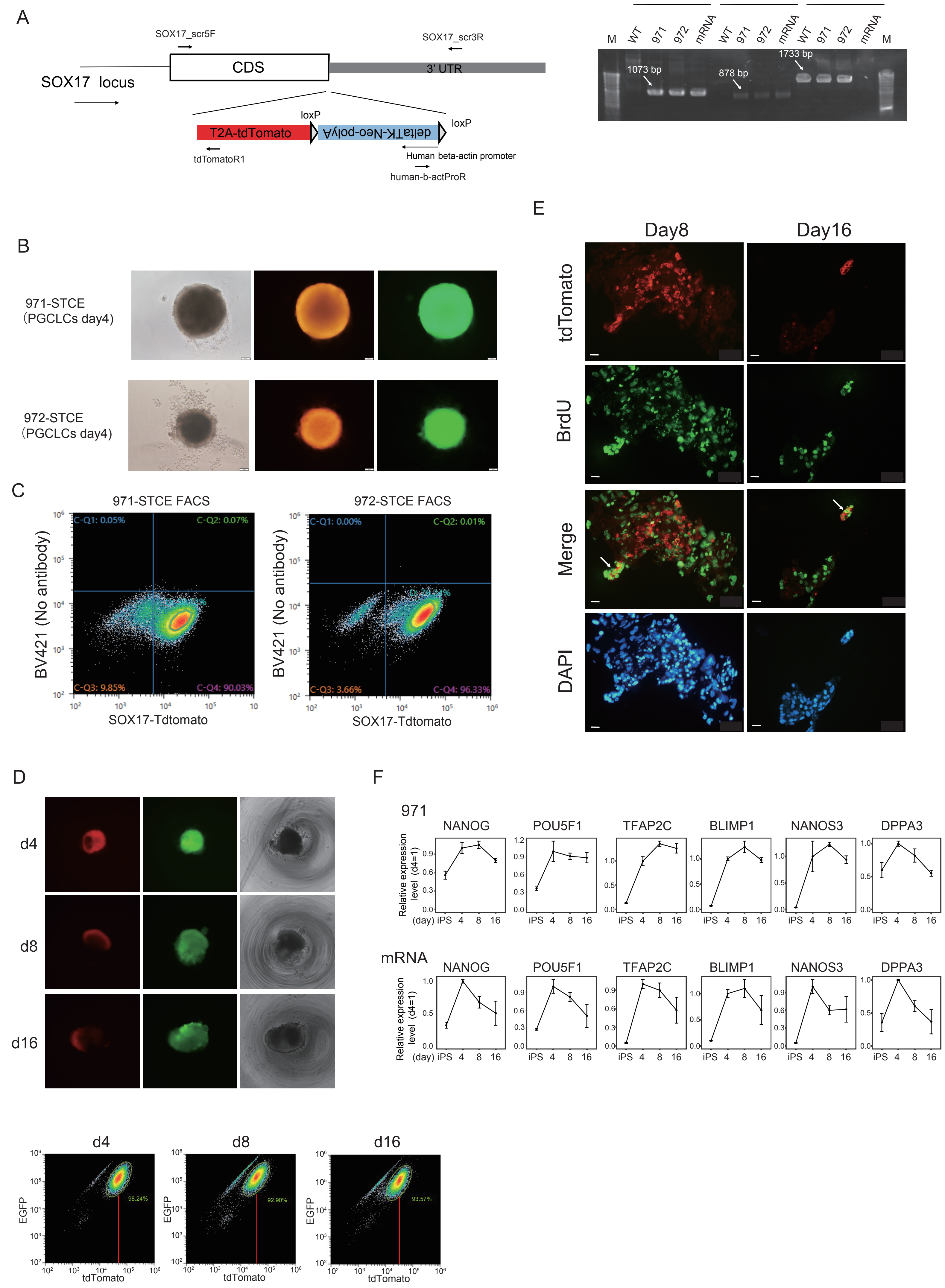
A
SOX17_scr5F
SOX17_scr3R
CDS
3' UTR
SOX17 locus
loxP
T2A-tdTomato
deltaTK-Neo-polyA
loxP
tdTomatoR1
Human beta-actin promoter
human-b-actProR
SOX17_scr5F tdTomatoR1
human-b-actProR SOX17_scr3R
SOX17_scr5F SOX17_scr3R
M WT 971 972 mRNA WT 971 972 mRNA WT 971 972 mRNA M
1073 bp
878 bp
1733 bp
B
971-STCE (PGCLCs day4)
972-STCE (PGCLCs day4)
C
971-STCE FACS
972-STCE FACS
BV421 (No antibody)
SOX17-Tdtomato
C-Q1: 0.05%
C-Q2: 0.07%
C-Q3: 9.85%
C-Q4: 90.03%
C-Q1: 0.00%
C-Q2: 0.01%
C-Q3: 3.66%
C-Q4: 96.33%
D
d4
d8
d16
EGFP
tdTomato
98.24%
92.90%
93.57%
E
Day8
Day16
tdTomato
BrdU
Merge
DAPI
F
971
mRNA
NANOG
POU5F1
TFAP2C
BLIMP1
NANOS3
DPPA3
Relative expression level (d4=1)
(day) iPS 4 8 16

Figure S2

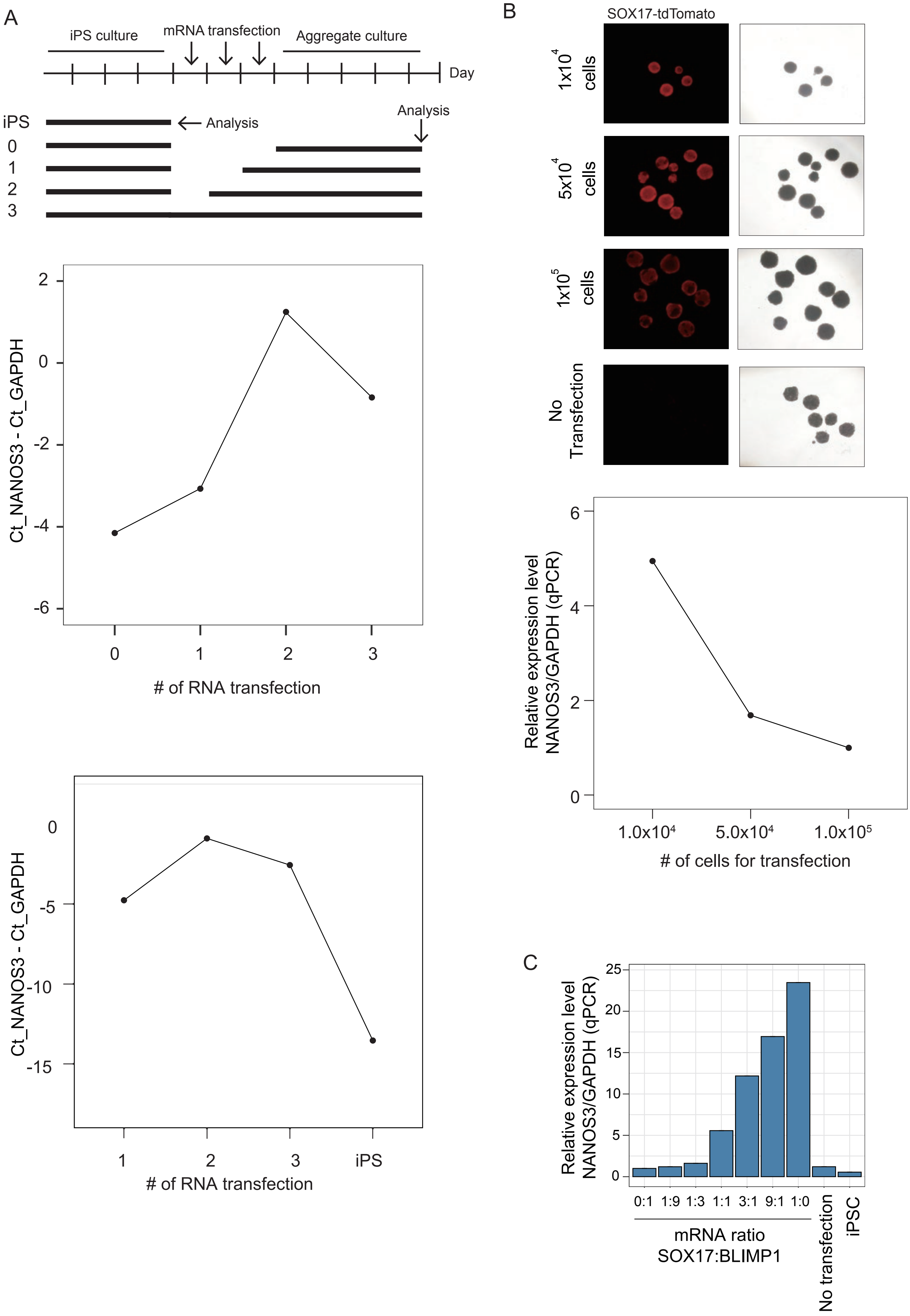

A
iPS culture
mRNA transfection
Aggregate culture
Day
iPS
0
1
2
3
Analysis
Analysis
Ct_NANOS3 - Ct_GAPDH
2
0
-2
-4
-6
0
1
2
3
# of RNA transfection
Ct_NANOS3 - Ct_GAPDH
0
-5
-10
-15
1
2
3
iPS
# of RNA transfection
B
SOX17-tdTomato
1x10^4 cells
5x10^4 cells
1x10^5 cells
No Transfection
Relative expression level NANOS3/GAPDH (qPCR)
6
4
2
0
1.0x10^4
5.0x10^4
1.0x10^5
# of cells for transfection
C
Relative expression level NANOS3/GAPDH (qPCR)
25
20
15
10
5
0
0:1
1:9
1:3
1:1
3:1
9:1
1:0
mRNA ratio
SOX17:BLIMP1
No transfection
iPSC

Figure S3

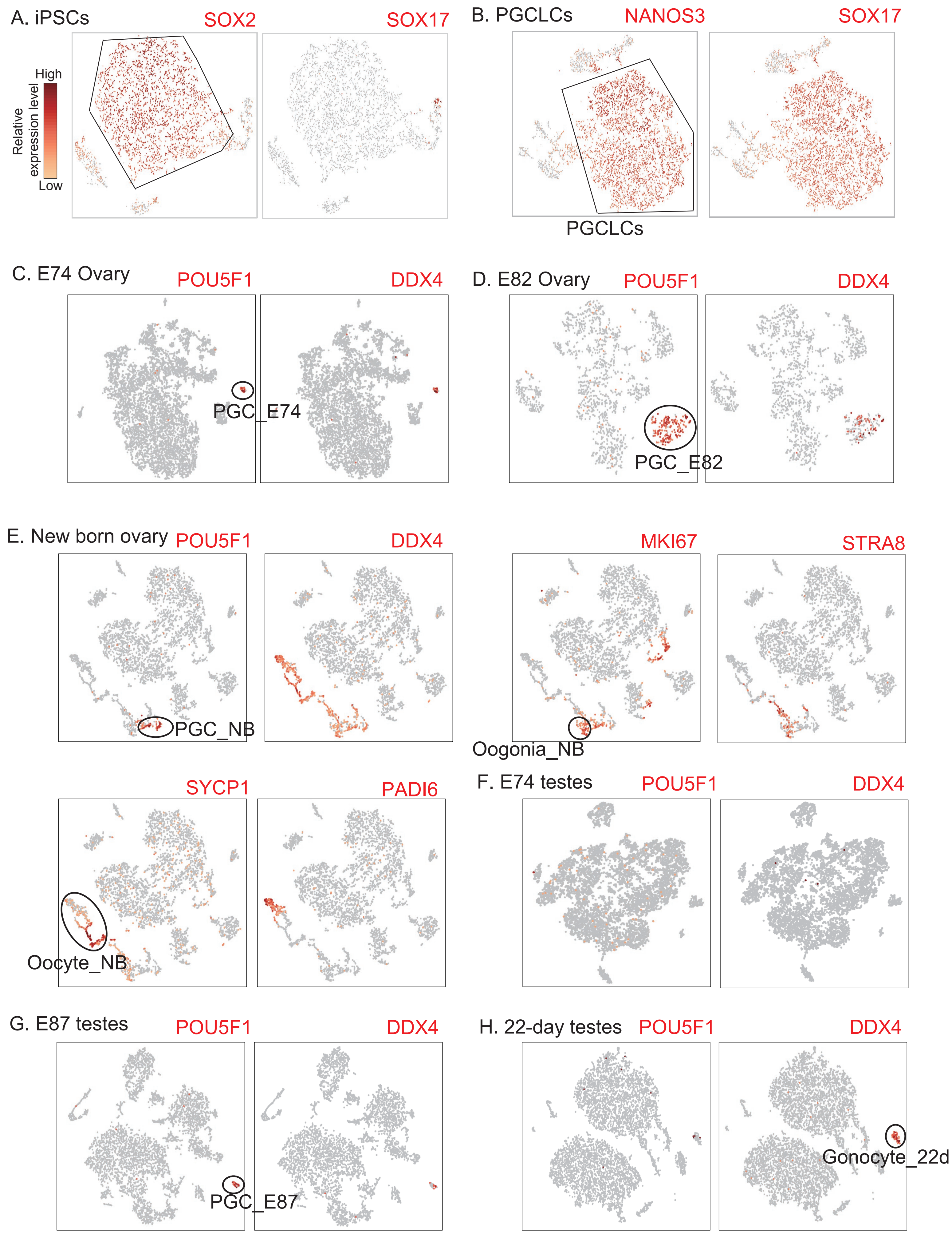

A. iPSCs
SOX2
SOX17
High
Relative expression level
Low
B. PGCLCs
NANOS3
SOX17
PGCLCs
C. E74 Ovary
POU5F1
DDX4
PGC_E74
D. E82 Ovary
POU5F1
DDX4
PGC_E82
E. New born ovary
POU5F1
DDX4
MKI67
STRA8
PGC_NB
Oogonia_NB
SYCP1
PADI6
Oocyte_NB
F. E74 testes
POU5F1
DDX4
G. E87 testes
POU5F1
DDX4
PGC_E87
H. 22-day testes
POU5F1
DDX4
Gonocyte_22d

Figure S4

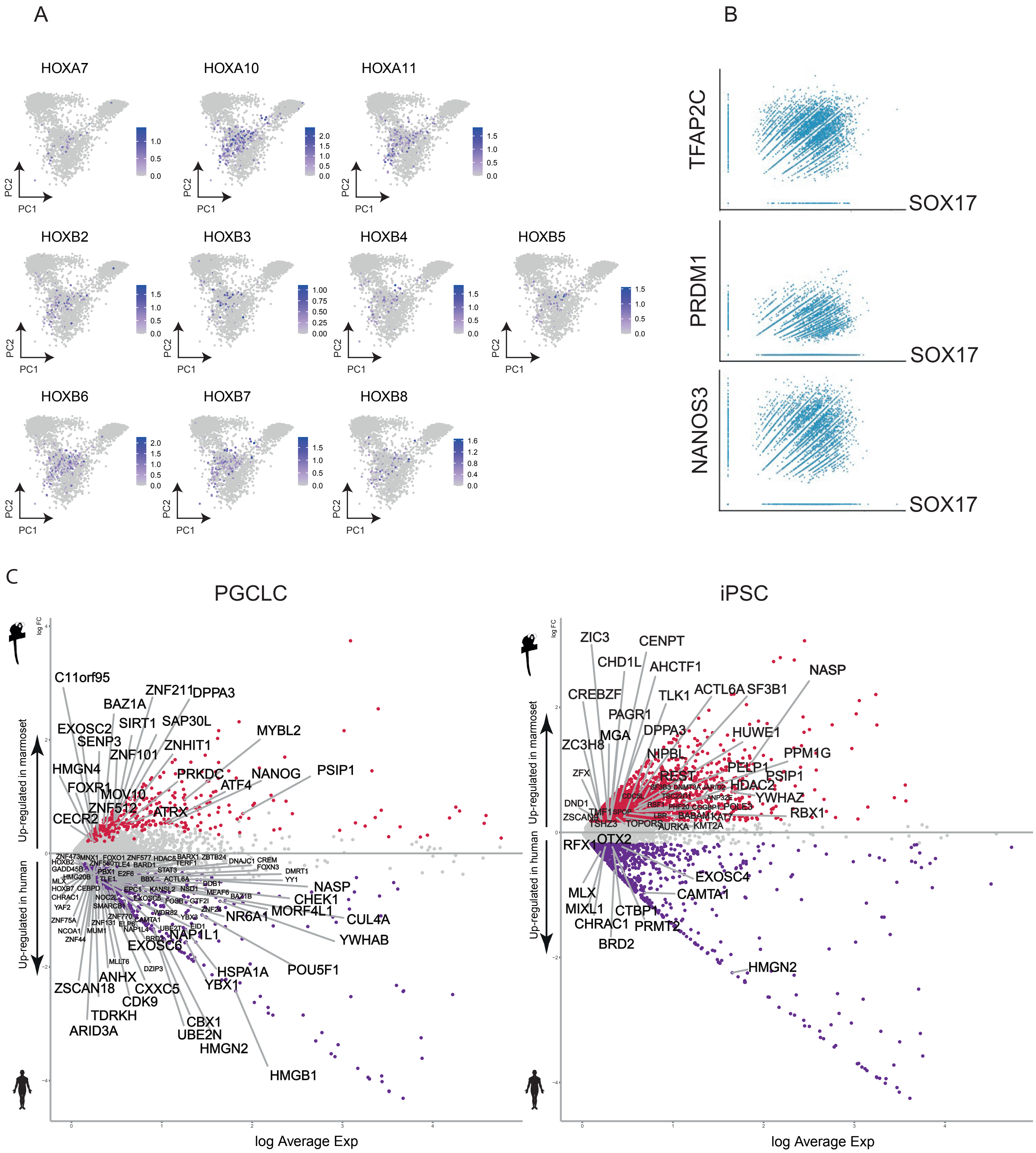
A
HOXA7
HOXA10
HOXA11
HOXB2
HOXB3
HOXB4
HOXB5
HOXB6
HOXB7
HOXB8
PC1
PC2
B
TFAP2C
PRDM1
NANOS3
SOX17
C
PGCLC
iPSC
Up-regulated in marmoset
Up-regulated in human
log FC
log Average Exp
C11orf95
ZNF211
DPPA3
BAZ1A
EXOSC2
SIRT1
SAP30L
MYBL2
SENP3
ZNHIT1
ZNF101
HMGN4
PRKDC
NANOG
PSIP1
FOXR1
ATF4
MOV10
ZNF512
CECR2
ATRX
NASP
CHEK1
MORF4L1
CUL4A
NR6A1
YWHAB
NAP1L1
EXOSC6
HSPA1A
POU5F1
ANHX
ZSCAN18
CXXC5
YBX1
CDK9
TDRKH
ARID3A
CBX1
UBE2N
HMGN2
HMGB1
ZIC3
CENPT
CHD1L
AHCTF1
CREBZF
TLK1
ACTL6A
SF3B1
NASP
PAGR1
MGA
DPPA3
HUWE1
ZC3H8
NIPBL
PPM1G
PELP1
PSIP1
REST
HDAC2
YWHAZ
RBX1
RFX1
OTX2
EXOSC4
CAMTA1
MLX
MIXL1
CTBP1
CHRAC1
PRMT2
BRD2
HMGN2

Figure S5

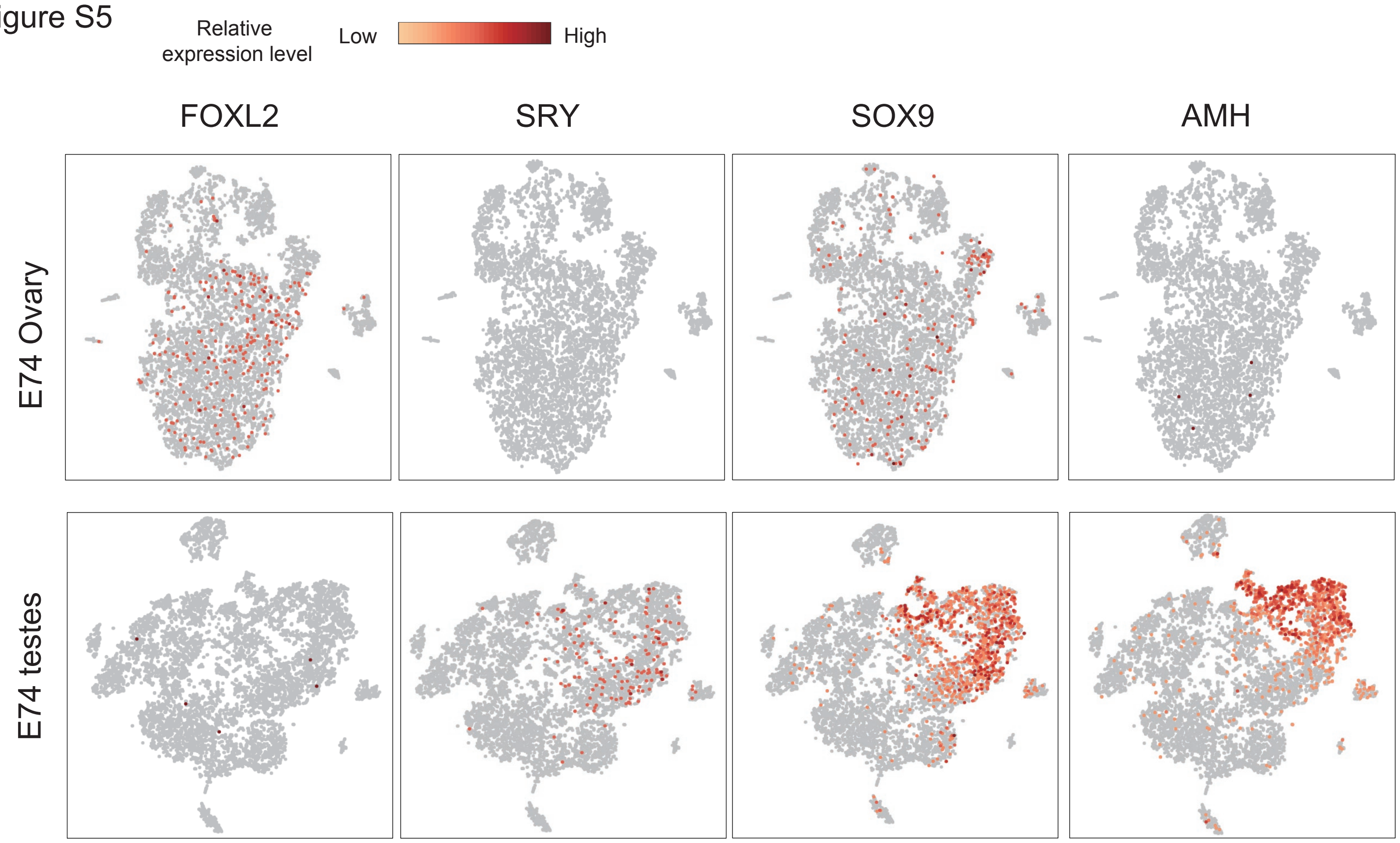
Relative expression level
Low
High
FOXL2
SRY
SOX9
AMH
E74 Ovary
E74 testes

Figure S6

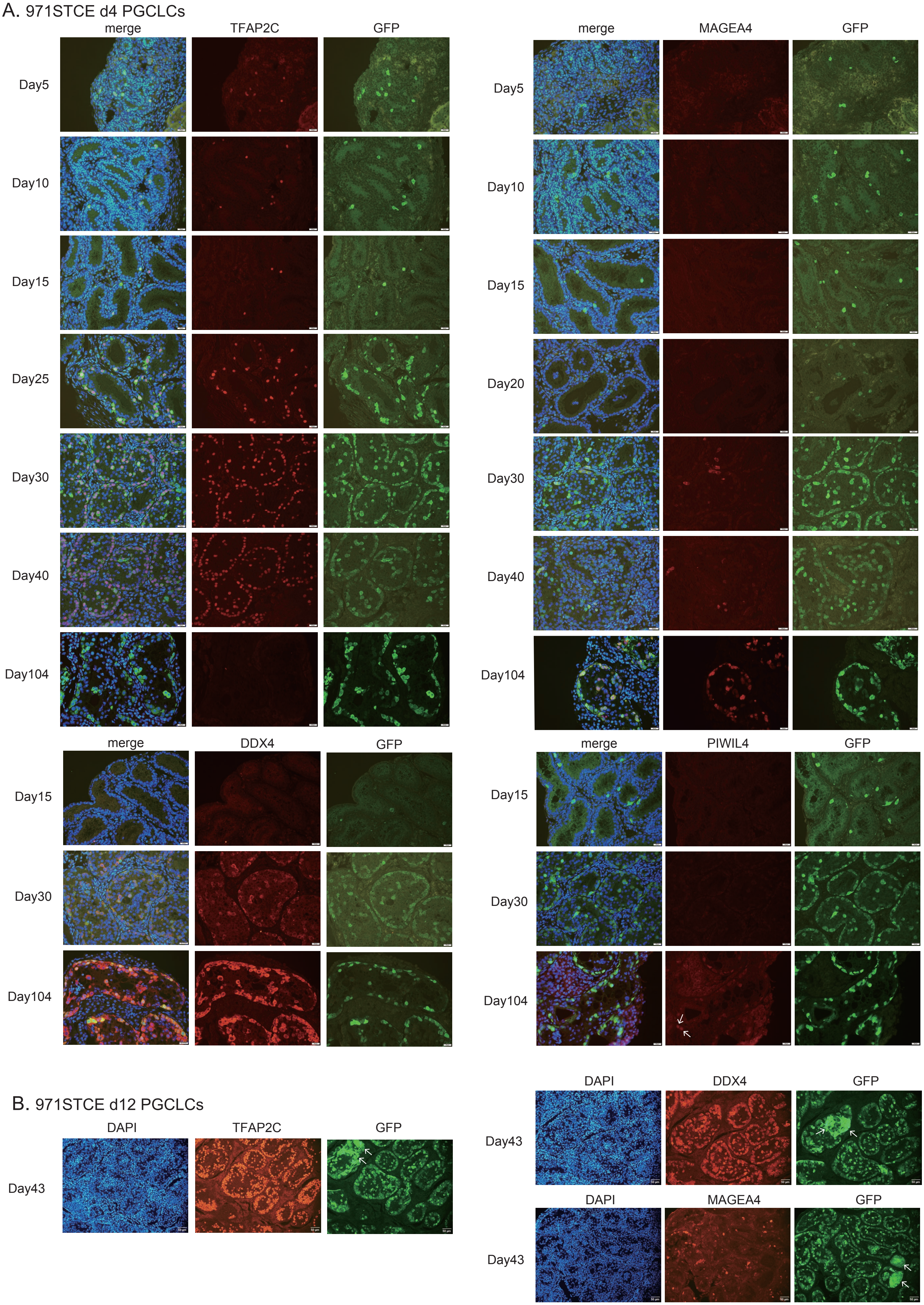
A. 971STCE d4 PGCLCs
merge
TFAP2C
GFP
Day5
Day10
Day15
Day25
Day30
Day40
Day104
merge
MAGEA4
GFP
Day5
Day10
Day15
Day20
Day30
Day40
Day104
merge
DDX4
GFP
Day15
Day30
Day104
merge
PIWIL4
GFP
Day15
Day30
Day104
B. 971STCE d12 PGCLCs
DAPI
TFAP2C
GFP
Day43
DAPI
DDX4
GFP
Day43
DAPI
MAGEA4
GFP
Day43

Figure S7

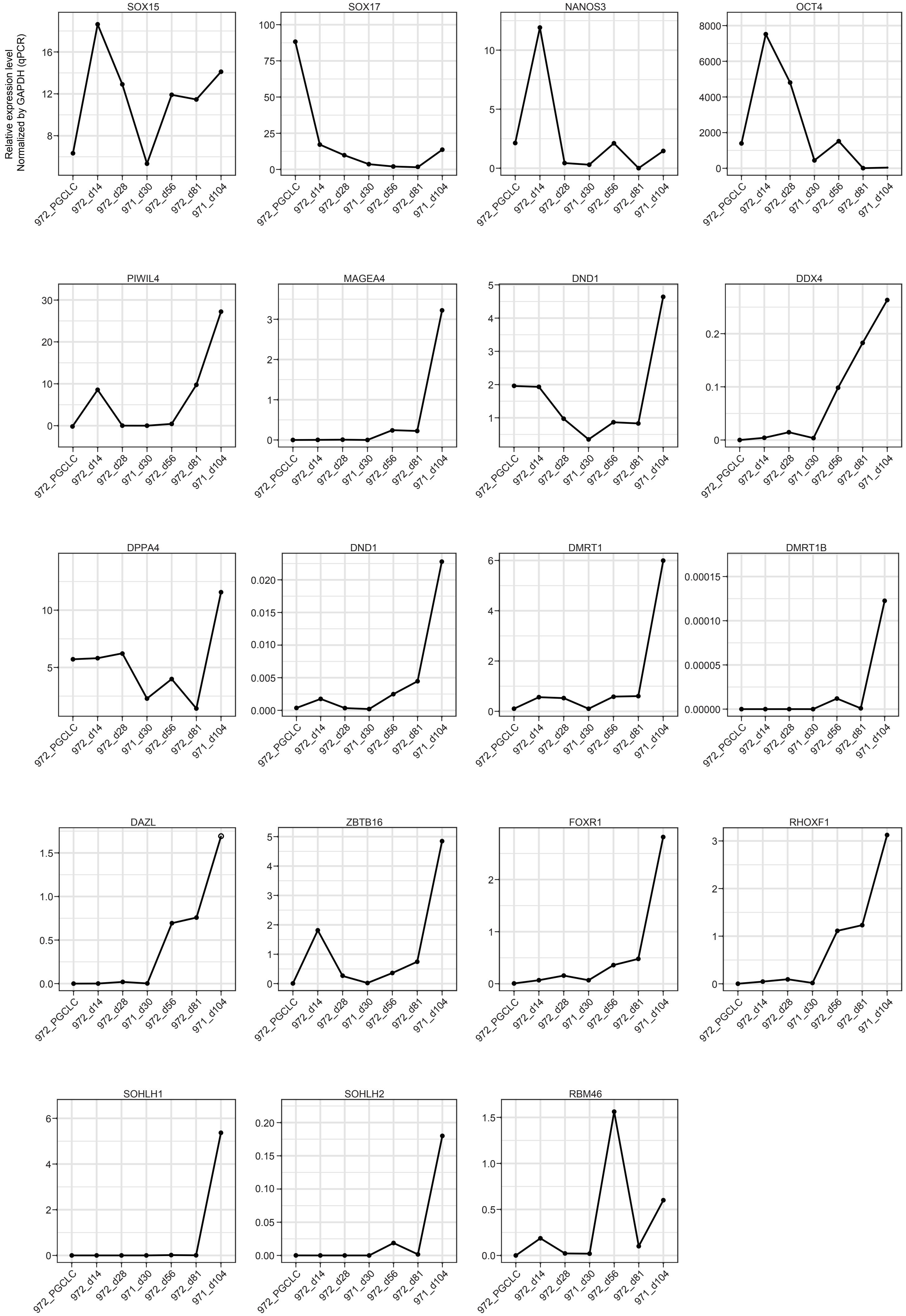
SOX15
SOX17
NANOS3
OCT4
PIWIL4
MAGEA4
DND1
DDX4
DPPA4
DND1
DMRT1
DMRT1B
DAZL
ZBTB16
FOXR1
RHOXF1
SOHLH1
SOHLH2
RBM46
Relative expression level
Normalized by GAPDH (qPCR)
972_PGCLC
972_d14
972_d28
971_d30
972_d56
972_d81
971_d104

Supplemental Figure Legends

Figure S1. PGCLC generation from 971-STCE and 972-STCE iPSCs

A. Schematic representation of knock-in of T2A-tdTomato cassette into *SOX17* locus. The expected recombination confirmed by PCR analyses (left and center). Heterozygosity (971 and 972) or homozygosity (mRNA) was checked by PCR indicated on the right. **B.** *SOX17*-tdTomato (ST) and CAG-EGFP (CE) expression in d4_PGCLCs from 971-STCE and 972-STCE iPS cells. Scale bars: 100 µm (top) and 50 µm (bottom). **C.** FACS analysis of d4_PGCLCs from 971-STCE and 972-STCE iPS cells. Many cells are positive for *SOX17*-tdTomato fluorescence. Antibodies for BV421 (Y-axis) fluorescence were not used here. **D.** FACS analyses of d4, d8, d16 PGCLCs. Fluorescent observation of aggregates used for FACS analyses. N=1. **E.** BrdU incorporation into *SOX17*-tdTomato-positive cells of d8_ and d16_ PGCLC aggregates (bottom). BrdU were added 48 hr before the sampling for immunohistochemical analyses. Proliferating PGCLCs are indicated by white arrows in the merge panels. Scale bars: 20 µm. N=1. **F.** Expression changes of key genes during PGCLC culture. FACS-purified PGCLCs were examine by qPCR. Error bars represent S.D. (N=2) of cells of different passage numbers. cDNAs used in Figure 1C were examined.

Figure S2. Determining the conditions for PGCLC induction using mRNAs

A. Determining the optimal number of transfections. In each well of 12-well plate, $8x10^4$ iPSCs (mRNA iPSCs) were seeded. As shown in the schematic diagram, after the indicated number of mRNA transfections, cells were aggregated in the low binding 96 well plate. The results of two independent experiments are shown. The expression levels of *NANOS3* and *GAPDH* in aggregates (day 4) were determined using qPCR. Delta Ct values are shown. The indicated values are the average of technical duplicates or triplicates. The results of two independent experiments are shown. **B.** Determining the optimal cell number of iPSCs (mRNA-ST iPSCs) used for transfection. PGCLC induction was performed using a standard procedure (two successive day mRNA transfection and aggregate culture for four days). PGCLC aggregates shown on the top were analyzed by qPCR (bottom). The average of technical triplicates are shown (N=1) separately. **C.** *SOX17* and *BLIMP1* mRNAs were together transfected at various ratios. After the two successive day transfection, PGCLCs were generated using floating aggregate culture. d4_PGCLCs were used for the analyses. The average of technical triplicates are shown (N=1).

Figure S3. Single cell RNA-seq analyses of developing marmoset iPSCs (A), PGCLC aggregates (B), ovaries (C-E) and testes (F-H)

iPSC and Germ cell populations used for PGCLC developmental analyses are indicated.

Figure S4. Characteristics of marmoset PGCLCs

A. PCA plot analyses of *HOX* genes. See Figure 2A for information of cells. **B.** Co-expression of *SOX17* with other markers *TFAP2C/PRDM1/NANOS3* in PGCLCs. PGCLCs identified in the analyses of Figure 2E were examined. **C.** Species specific expression of transcription factors in human and marmoset PGCLCs (left) and iPSCs (right). PGCLCs and iPSCs extracted from 10X data were analyzed. Transcription factors were indicated in the MA plot.

Figure S5. The expression of genes involved in sex differentiation in E74 ovaries and testes

Figure S6. The expression of PGC and gonocyte marker genes in xrtestes

Immunofluorecent analyses of marker gene expression in developing xrtestes. Day4 (A) and Day 12 (B) PGCLCs from 971-STCE iPSCs were used for the generation of xrtestes. Days after the transplantation were indicated on the left side of the panels. Xrtestis experiments conducted were listed in Table S2. Scale bar: 20 μm. *PIWIL4*-positive cells are indicated by white arrows in (A). Cancerous cells are indicated by white arrows in (B). The cancerous cells highly express EGFP, though germ cells express it only weakly. In addition, they do not express a PGC marker (*TFAP2C*), which is expressed in almost all seminomas (Pauls et al., 2005).

Figure S7. qPCR analyses of PGC and gonocyte marker genes in xrtestes

qPCR analyses of marker gene expression in developing xrtestes (971-STCE and 972-STCE). Only one sample was examined in each stage, and the indicated values are the average of technical duplicates. The expression level was normalized to that of *GAPDH*. The value was further normalized to the expression level in the marmoset adult testes. The expression level in the adult testes corresponds to 1.

Table S1. Sample and cell line information used in this study

Samples used for 10x library generation

#	Cells or samples (cell line name)	Library type	No. of cells analyzed[a]	Animal ID	Sex	Oocyte ID	Sperm ID	Note
1	iPSC culture (mRNA iPS)	10x v3	4,138	I 2965F	F			Derived from liver cells.
2	PGCLC aggregate (mRNA iPS)	10x v3	7,284	I 2965F	F			Induced from #1
3	E74 ovaries	10x v2	8,001		F	I4698F or I5463F	I4213M or YI034M	
4	E74 testes	10x v2	8,664		M	I4698F or I5463F	I4213M or YI034M	
5	E82 ovaries	10x v3	5,970		F	I689F	YI810M	
6	E87 testes	10x v2	7,230		M	I4750F or I4962F	I5057M or I764M	
7	Newborn ovaries	10x v2	14,308	From RIKEN	F			
8	22 day testes	10x v2	9,789	I 880M	M			
9	3yr10mnth testes	10x v2	12,335	I 6093M	M			

[a] Number of cells after Cellranger analyses

Samples used for simultaneous scRNA/scBS-seq aanalyses

#	Cells or samples	Library type	No. of cells analyzed	Animal ID	Sex	Oocyte ID	Sperm ID	Note
9	iPS/PGCLC (971-STCE)	scRNA-seq/scBS-seq	29 scRNA-seq/30 scBS-seq	I 971M	M			Derived from ear cells.<br>(RNA-seq/0 BS-seq[b])<br>iPS 3/0[b]<br>d4_PGCLC 2/3[c]<br>d12_PGCLC 2/3[c]<br>d5_xrtestes 3/3<br>d10_xrtestes 2/3[c]<br>d15_xrtestes 1/2[c]<br>d20_xrtestes 3/4[c]<br>d25_xxrtestes 3/2[c,d]<br>d30_xrtestes 3/3<br>d104_xrtestes 8/8
10	iPS/PGCLC (972-STCE)			I 972M	M			Derived from ear cells.

[b] scRNA-seq was only conducted

[c] For one sample, scBS-seq was only conducted

[d] For two samples, scRNA-seq was only conducted

Table S2. Summary of transplantation experiments

Cell line	PGCLCs	Sampling	Status	Marker expression	scRNA/scBS-seq
971 STCE	d4	d5	reconstituted testes		○
971 STCE	d4	d10	reconstituted testes		○
971 STCE	d15	d10	reconstituted testes		
972 STCE	d4	d12	reconstituted testes		
971 STCE	d4	d14	reconstituted testes	TFAP2C(+)	
972 STCE	d4	d14	reconstituted testes	TFAP2C(+)	
972 STCE	d4	d14	reconstituted testes		
971 STCE	d4	d15	reconstituted testes	TFAP2C(+)	○
971 STCE	d4	d20	reconstituted testes	TFAP2C(+)	○
971 STCE	d15	d21	reconstituted testes	TFAP2C(+)	
971 STCE	d4	d25	reconstituted testes	TFAP2C(+)	○
972 STCE	d4	d28	reconstituted testes	TFAP2C(+), DDX4(+)	
972 STCE	d4	d28	reconstituted testes		
971 STCE	d4	d28	reconstituted testes		
971 STCE	d4	d28	canceration		
971 STCE	d4	d30	reconstituted testes	TFAP2C(+), DDX4(+)	○
971 STCE	d4	d40	reconstituted testes	TFAP2C(+), DDX4(+)	
971 STCE	d4	d40	canceration		
971 STCE	d4	d42	reconstituted testes		
971 STCE	d12	d43	reconstituted testes and partial canceration	TFAP2C(+), DDX4(+), MAGEA4(+)	
972 STCE	d4	d47	reconstituted testes		
972 STCE	d4	d56	reconstituted testes	TFAP2C(+), DDX4(+), MAGEA4(+)	
972 STCE	d4	d56	reconstituted testes		
972 STCE	d4	d63	canceration		
972 STCE	d4	d81	reconstituted testes	DDX4(+), MAGEA4(+)	
972 STCE	d4	d81	reconstituted testes		
971 STCE	d4	d84	reconstituted testes and partial canceration	TFAP2C(+), DDX4(+)	
971 STCE	d4	d84	reconstituted testes and partial canceration		
971 STCE	d4	d84	reconstituted testes	TFAP2C(+), DDX4(+)	
971 STCE	d4	d84	reconstituted testes	TFAP2C(+), DDX4(+)	
972 STCE	d4	d90	canceration		
972 STCE	d4	d90	canceration		
972 STCE	d4	d90	reconstituted testes	TFAP2C(+), DDX4(+)	

972 STCE	d4	d90	reconstituted testes and partial canceration	TFAP2C(+), DDX4(+)	
972 STCE	d4	d96	reconstituted testes		
971 STCE	d4	d104	reconstituted testes and partial canceration	DDX4(+), MAGEA4(+), PIWIL4(+)	○
971 STCE	d15	d109	canceration		
971 STCE	d4	d113	reconstituted testes		
972 STCE	d4	d137	canceration		
972 STCE	d4	d137	canceration		
972 STCE	d4	d165	canceration		
972 STCE	d4	d165	canceration		
972 STCE	d4	d165	canceration		

Table S3. Summary statics for simultaneous scRNA-seq and scBS-seq analyses

scBS-seq	% of DNAme	% of mapping	# of total reads	
PGCLCs_d4-1	62.4	22.2	9271248	*scBS-seq only
PGCLCs-d4-2	60.3	29.5	5395186	
PGCLCs-d4-3	60.6	31.5	7753392	
PGCLCs_d12-1	42.8	38.9	7654536	
PGCLCs_d12-2	48.6	28.1	6006990	
PGCLCs_d12-3	36.5	22.3	3636159	*scBS-seq only
d5-1	43.3	30.4	7842101	
d5-3	39.3	19.5	6601570	
d5-4	47.8	24.2	8367382	
d10-1	28.4	34.1	6996434	
d10-2	40.3	19.9	5676850	*scBS-seq only
d10-8	31.8	31.2	4445992	
d15-3	11.7	33.5	5630184	*scBS-seq only
d15-8	33.3	33.2	7078191	
d20-1	19.2	29.6	5579726	*scBS-seq only
d20-2	17.5	23.4	5201037	
d20-3	14.6	35.8	5801722	
d20-4	10.2	31.7	7145941	
d25-1	10.9	19.2	5130107	*scBS-seq only
d25-8	8	29.9	7365294	
d30-1	11.4	39.2	5419758	
d30-3	7.9	30.9	6966065	
d30-4	8.9	30.1	6806686	
d104-2	3.2	1.6	5378037	
d104-21	4.2	1.9	4513287	
d104-36	5.1	2.1	5780035	
d104-62	3.6	2.8	7248465	
d104-74	3.8	3.6	5372929	
d104-108	6.6	5	5413307	
d104-139	3.5	6.3	5284600	
d104-144	4.3	9.7	6700814	

total:31 cells

scRNA-seq	# of total genic UMIs	# of total genes	Index sequences	Cell barcodes	
PGCLC_d4-2	13265	2860	103:Biotin/CAAGCAGAAGACGGCATACGAGATAGAGTAGTGACTG GAGTTCAGACGTGTGCTCTTCCGATC	ACAGCTACACGTGAGA	
PGCLC_d4-3	55505	5832	103:Biotin/CAAGCAGAAGACGGCATACGAGATAGAGTAGTGACTG GAGTTCAGACGTGTGCTCTTCCGATC	AGCGTATAGACGACGT	
PGCLC_12-1	8917	2929	105:Biotin/CAAGCAGAAGACGGCATACGAGATGCCAATGTGACTG GAGTTCAGACGTGTGCTCTTCCGATC	CACATAGTCGCACTCT	
PGCLC_12-2	11069	3311	105:Biotin/CAAGCAGAAGACGGCATACGAGATGCCAATGTGACTG GAGTTCAGACGTGTGCTCTTCCGATC	CCTACACTCTACTATC	
d5-1	9196	1948	106:Biotin/CAAGCAGAAGACGGCATACGAGATCTTGTAGTGACTG GAGTTCAGACGTGTGCTCTTCCGATC	GACACGCTCAGTCAGT	
d5-3	4519	1769	105:Biotin/CAAGCAGAAGACGGCATACGAGATGCCAATGTGACTG GAGTTCAGACGTGTGCTCTTCCGATC	GCAATCACAATGTTGC	
d5-4	28842	4036	104:Biotin/CAAGCAGAAGACGGCATACGAGATGCCAATGTGACTG GAGTTCAGACGTGTGCTCTTCCGATC	GGTGCGTAGGTGCACA	
d10-1	11944	3055	104:Biotin/CAAGCAGAAGACGGCATACGAGATGCCAATGTGACTG GAGTTCAGACGTGTGCTCTTCCGATC	GTTCTCGTCTGCTGTC	
d10-8	23130	3551	104:Biotin/CAAGCAGAAGACGGCATACGAGATGCCAATGTGACTG GAGTTCAGACGTGTGCTCTTCCGATC	AGCGTATAGACGACGT	
d15-8	20608	3958	103:Biotin/CAAGCAGAAGACGGCATACGAGATAGAGTAGTGACTG GAGTTCAGACGTGTGCTCTTCCGATC	ATCATCTGTAGCGTAG	
d20-2	15090	3372	104:Biotin/CAAGCAGAAGACGGCATACGAGATGCCAATGTGACTG GAGTTCAGACGTGTGCTCTTCCGATC	CACATAGTCGCACTCT	
d20-3	17330	3941	103:Biotin/CAAGCAGAAGACGGCATACGAGATAGAGTAGTGACTG GAGTTCAGACGTGTGCTCTTCCGATC	CCTACACTCTACTATC	
d20-4	64849	3929	104:Biotin/CAAGCAGAAGACGGCATACGAGATGCCAATGTGACTG GAGTTCAGACGTGTGCTCTTCCGATC	CGAGCACAGATAGCAT	
d25-3	13434	2887	103:Biotin/CAAGCAGAAGACGGCATACGAGATAGAGTAGTGACTG GAGTTCAGACGTGTGCTCTTCCGATC	GACACGCTCAGTCAGT	*scRNA-seq only
d25-4	8558	2976	104:Biotin/CAAGCAGAAGACGGCATACGAGATGCCAATGTGACTG GAGTTCAGACGTGTGCTCTTCCGATC	GCAATCACAATGTTGC	*scRNA-seq only
d25-8	24894	4146	104:Biotin/CAAGCAGAAGACGGCATACGAGATGCCAATGTGACTG GAGTTCAGACGTGTGCTCTTCCGATC	CCTACACTCTACTATC	
d30-1	72746	6109	103:Biotin/CAAGCAGAAGACGGCATACGAGATAGAGTAGTGACTG GAGTTCAGACGTGTGCTCTTCCGATC	GGTGCGTAGGTGCACA	
d30-3	20653	4516	103:Biotin/CAAGCAGAAGACGGCATACGAGATAGAGTAGTGACTG GAGTTCAGACGTGTGCTCTTCCGATC	GTTCTCGTCTGCTGTC	
d30-4	16235	3492	103:Biotin/CAAGCAGAAGACGGCATACGAGATAGAGTAGTGACTG GAGTTCAGACGTGTGCTCTTCCGATC	TACTCATCACAGTCGC	
d104-2	48848	6112	105:Biotin/CAAGCAGAAGACGGCATACGAGATGCCAATGTGACTG GAGTTCAGACGTGTGCTCTTCCGATC	ACAGCTACACGTGAGA	
d104-21	14859	4370	106:Biotin/CAAGCAGAAGACGGCATACGAGATCTTGTAGTGACTG GAGTTCAGACGTGTGCTCTTCCGATC	CACATAGTCGCACTCT	
d104-36	31217	5183	105:Biotin/CAAGCAGAAGACGGCATACGAGATGCCAATGTGACTG GAGTTCAGACGTGTGCTCTTCCGATC	ATCATCTGTAGCGTAG	
d104-62	22689	4778	105:Biotin/CAAGCAGAAGACGGCATACGAGATGCCAATGTGACTG GAGTTCAGACGTGTGCTCTTCCGATC	TCGGTAATCACGCATA	
d104-74	12425	4039	106:Biotin/CAAGCAGAAGACGGCATACGAGATCTTGTAGTGACTG GAGTTCAGACGTGTGCTCTTCCGATC	GCAATCACAATGTTGC	
d104-108	12594	4458	106:Biotin/CAAGCAGAAGACGGCATACGAGATCTTGTAGTGACTG GAGTTCAGACGTGTGCTCTTCCGATC	GTTCTCGTCTGCTGTC	
d104-139	32917	3962	106:Biotin/CAAGCAGAAGACGGCATACGAGATCTTGTAGTGACTG GAGTTCAGACGTGTGCTCTTCCGATC	GGTGCGTAGGTGCACA	
d104-144	12421	4160	106:Biotin/CAAGCAGAAGACGGCATACGAGATCTTGTAGTGACTG GAGTTCAGACGTGTGCTCTTCCGATC	TGTCCCAGTTTGGCGC	
iPSC-1	26593	2868	106:Biotin/CAAGCAGAAGACGGCATACGAGATCTTGTAGTGACTG GAGTTCAGACGTGTGCTCTTCCGATC	ACAGCTACACGTGAGA	*scRNA-seq only
iPSC-2	81459	6495	106:Biotin/CAAGCAGAAGACGGCATACGAGATCTTGTAGTGACTG GAGTTCAGACGTGTGCTCTTCCGATC	AGCGTATAGACGACGT	*scRNA-seq only
iPSC-3	115155	6873	104:Biotin/CAAGCAGAAGACGGCATACGAGATGCCAATGTGACTG GAGTTCAGACGTGTGCTCTTCCGATC	ATCATCTGTAGCGTAG	*scRNA-seq only

total: 30 cells

Table S4. The list of primer sequences used in this study

#	Name	
1	Sox17_5UTR	CTCAGAGAGAACCCACCACC ATGAGCAGCCCGGATG
2	Sox17_R_Asc1	TTAAC GGCGCGCC TCACACATCAGGATAGTTGCAG
3	5UTR_HBA_Not1	GATCGTACGCGGCCGCTCTTCTGGTCCCCACAGACTCAGAGA GAACCCAC
4	Sox17 5’ arm F	ACGCGTTACGTATCGGATCC TGCAGCACATGCAGGACCAC
5	Sox17 5’ arm R	CTGCCCTCTCCGGATCCCACATCAGGATAGTTGCAGTAATAC AC
6	Sox17 3’ arm F	CGCTAGCGAATTCTAGGATCC GGA GAG CTAAGGAAGTCCTCAG
7	Sox17 3’ arm R	ACGAATTCAGATTCGGATCC AGCCTCTGTAGGCAGGTCAAG
8	sgRNA-Universal-rev-primer	GTTT GAATTC AAAAAAA GCACCGACTCGGTGCCACTTTTTCAAGTTGATAA CGGACTAGCCTTATTTTAACTTGCTATTTCTAGCTCTAA
9	pHL_Sox17_1	CTT GGATCC G AAGCAGTGTTACACACTTCC GTTTTAGA GCTA GAAA TAGCA
10	pHL_Sox17_2	CTT GGATCC G ACACACTTCCTGGAGAGCTA GTTTTAGA GCTA GAAA TAGCA
11	pHL_Sox17_3	CTT GGATCC G GAGGACTTCCTTAGCTCTCC GTTTTAGA GCTA GAAA TAGCA
12	Sox17_scr5F	CAAGTCGTGGAAGGCGCTGAC
13	tdTomatoR1	GAAGCGCATGAACTCTTTGATGACCTC
14	Sox17_scr3R	AGGCAAACTCAACGTTGAGGAGTG
15	human-b-actProR	GACATCTCTTGGGCACTGAG
16	SOX15-qPCR F	CTACTCGACAGCCTACTTGCC
17	SOX15-qPCR R	CTGGAGCCTAGGGTCACTCTG
18	PIWIL4-qPCR F	CAGGTTCCAGTGGAATACCTGTG
19	PIWIL4-qPCR R	CACATGGTACTGGTATAGCTG
20	MAGEA4-qPCR F	TGGGAGGAGCTGAGTGTGATG
21	MAGEA4-qPCR R	CAGAGATACATCTCCAAGTCACTC
22	DAZL-qPCR F	CTCCGGCTTATTCATCTGTAAACT
23	DAZL-qPCR R	GATGCACTCTTTTATCCCTGAAGT
24	DMRT1B-qPCR F	TGCAGTAGGCCCTGAGTACC
25	DMRT1B-qPCR R	GGTAGTAGCTTGGCTGGAAGTCTC
26	FOXR1-qPCR F	GCAGAAACTTGCCAGGTATAAACT
27	FOXR1-qPCR R	GATCCTCCTTCTTCATAAGTCCTG
28	RHOXF1-qPCR F	GTGTTAACAAGAAGGGAACTTGCT

29 RHOXF1-qPCR R	ATGTAGAAACTGTCATCTGGATCG
30 SOHLH1-qPCR F	CTCTGGTGACTGTGGGTCCTA
31 SOHLH1-qPCR R	CGTCAACTGTAGAACATTCTCCTT
32 ZBTB16-qPCR F	ACTATAGGGTGCACACAGGTGAGA
33 ZBTB16-qPCR R	GAGGTACGTCTTCTCTATCCTCCA
34 CREM-qPCR F	GTGGAACAATCCAGATTTCTAACC
35 CREM-qPCR R	GTAGTAGGAGCTCGGATCTGGTAA
36 DMRT1-qPCR F	ATTCTTACTACCCACCTCCCTCTT
37 DMRT1-qPCR R	ATGACAGGAGTGACTGTAAAGCTG
38 SOHLH2-qPCR F	CAGGACATGCAGGTGATATGAC
39 SOHLH2-qPCR R	CTAGAGATTCAGGGCAGGCAGA
40 DND1-qPCR F	GGGCAGATCGCTCTGCTC
41 DND1-qPCR R	CTCTCCACAGAGGTGTGATTG
42 SOX17-qPCR F	CCGAGCTGAGCAAGATGCTG
43 SOX17-qPCR R	GTGGTCCTGCATGTGCTGCAC
44 NANOS3-qPCR F	CTTCTGCCCACTCACTGGACAG
45 NANOS3-qPCR R	CTCAGACTTCCCGGCACCTCTG
46 DDX4-qPCR F	AAGTATTAACAGATGCTCAACAGGATGT
47 DDX4-qPCR R	TGAAGCCAGGAATGTATGCACTA
48 DPPA4-qPCR F	TGGGTAAGCAAAGGCACACAG
49 DPPA4-qPCR R	CTGGTGTCAGCAACTAAAGCTAAGCAC
50 GAPDH-qPCR F	TGCTGGCGCTGAGTATGTG
51 GAPDH-qPCR R	AGCCCCAGCCTTCTCCAT
52 SOX17-endo-F	ACCGCGACAGGCTAGAACAC
53 SOX17-endo-R	GGTCTGGCTCTGGTCGTCAC
54 SOX17-exo-F	CCCACAGACTCAGAGAGAAC
55 SOX17-exo-R	GGTCTGGCTCTGGTCGTCAC
56 NANOG-qPCR F	TGCTGAGATGCCTCACACAG
57 NANOG-qPCR R	TCACTGCAGGGCTACTCTCT
58 DPPA3-qPCR-F	CGGTCATCAGTTTCTGCTGTG
59 DPPA3-qPCR-R	TCCTGGTAAGTTTCTCCGGTG
60 OCT4-qPCR F	GGAACAAAACACGGAGGAGTC
61 OCT4-qPCR R	CAGGGTGATCCTCTTCTGCTTC
62 BLIMP1-qPCR F	CGGATATGACTCTGTGGACAGAG
63 BLIMP1-qPCR R	CACGCCAATAACCTCTTTGCTG
64 CB16UMI12-RT primer1	TCA GAC GTG TGC TCT TCC GAT CTA ATC GGT GTT GAT TCG NNN NNN NNN NNN TTT TTT TTT TTT TTT TTT TTT TTT T
65 BC16UMI12-RT primer2	TCA GAC GTG TGC TCT TCC GAT CTA CAG CTA CAC GTG AGA NNN NNN NNN NNN TTT TTT TTT TTT TTT TTT TTT TTT T

66	BC16UMI12-RT primer3	TCA GAC GTG TGC TCT TCC GAT CTA GCG TAT AGA CGA CGT NNN NNN NNN NNN TTT TTT TTT TTT TTT TTT TTT TTT T
67	BC16UMI12-RT primer4	TCA GAC GTG TGC TCT TCC GAT CTA TCA TCT GTA GCG TAG NNN NNN NNN NNN TTT TTT TTT TTT TTT TTT TTT TTT T
68	BC16UMI12-RT primer5	TCA GAC GTG TGC TCT TCC GAT CTC ACA TAG TCG CAC TCT NNN NNN NNN NNN TTT TTT TTT TTT TTT TTT TTT TTT T
69	BC16UMI12-RT primer6	TCA GAC GTG TGC TCT TCC GAT CTC CTA CAC TCT ACT ATC NNN NNN NNN NNN TTT TTT TTT TTT TTT TTT TTT TTT T
70	BC16UMI12-RT primer7	TCA GAC GTG TGC TCT TCC GAT CTC GAG CAC AGA TAG CAT NNN NNN NNN NNN TTT TTT TTT TTT TTT TTT TTT TTT T
71	BC16UMI12-RT primer8	TCA GAC GTG TGC TCT TCC GAT CTC TGA AGT AGT ATT GGA NNN NNN NNN NNN TTT TTT TTT TTT TTT TTT TTT TTT T
72	BC16UMI12-RT primer9	TCA GAC GTG TGC TCT TCC GAT CTG ACA CGC TCA GTC AGT NNN NNN NNN NNN TTT TTT TTT TTT TTT TTT TTT TTT T
73	BC16UMI12-RT primer10	TCA GAC GTG TGC TCT TCC GAT CTG CAA TCA CAA TGT TGC NNN NNN NNN NNN TTT TTT TTT TTT TTT TTT TTT TTT T
74	BC16UMI12-RT primer11	TCA GAC GTG TGC TCT TCC GAT CTG GTG CGT AGG TGC ACA NNN NNN NNN NNN TTT TTT TTT TTT TTT TTT TTT TTT T
75	BC16UMI12-RT primer12	TCA GAC GTG TGC TCT TCC GAT CTG TTC TCG TCT GCT GTC NNN NNN NNN NNN TTT TTT TTT TTT TTT TTT TTT TTT T
76	BC16UMI12-RT primer13	TCA GAC GTG TGC TCT TCC GAT CTT ACT CAT CAC AGT CGC NNN NNN NNN NNN TTT TTT TTT TTT TTT TTT TTT TTT T
77	BC16UMI12-RT primer14	TCA GAC GTG TGC TCT TCC GAT CTT CGG TAA TCA CGC ATA NNN NNN NNN NNN TTT TTT TTT TTT TTT TTT TTT TTT T
78	BC16UMI12-RT primer15	TCA GAC GTG TGC TCT TCC GAT CTT GTC CCA GTT TGG CGC NNN NNN NNN NNN TTT TTT TTT TTT TTT TTT TTT TTT T
79	BC16UMI12-RT primer16	TCA GAC GTG TGC TCT TCC GAT CTT TGA CTT GTA CTC GCG NNN NNN NNN NNN TTT TTT TTT TTT TTT TTT TTT TTT T
80	TSO primer	/5Me-isodC//iisodG//iMe-isodC/AAGCAGTGGTATCAACGCAGAGTACATrGrG+G
81	3' Anchoerd primer	GTGACTGGAGTTCAGACGTGTGCTCTTCCGATC
82	ISPCR primer	AAGCAGTGGTATCAACGCAGAGT
83	scBS-P5-N9-oligo1	CTACACGACGCTCTTCCGATCTNNNNNNNNN
84	scBS-oligo2	GACTGGAGTTCAGACGTGTGCTCTTCCGATCTNNNNNNNNN
85	Biotin-index-primer103	Biotin/CAAGCAGAAGACGGCATACGAGATAGAGTAGTGACTGGAGTTCAGACGTGTGCTCTTCCGATC

86	Biotin-index-primer104	Biotin/CAAGCAGAAGACGGCATACGAGATAGTCCAGTGACTGGAGTTCAGACGTGTGCTCTTCCGATC
87	Biotin-index-primer105	Biotin/CAAGCAGAAGACGGCATACGAGATGCCAATGTGACTGGAGTTCAGACGTGTGCTCTTCCGATC
88	Biotin-index-primer106	Biotin/CAAGCAGAAGACGGCATACGAGATCTTGTAGTGACTGGAGTTCAGACGTGTGCTCTTCCGATC
89	QP2	CAAGCAGAAGACGGCATACGA
90	Modified_P5 primer	AATGATACGGCGACCACCGAGATCTACAC[index]ACACTCTTTCCCTACACGAC

Supplemental Experimental Procedures

Marmoset housing and samplings

All animal experiments using marmosets and mice were approved by the Animal Committee of the Central Institute for Experimental Animals (CIEA) (Approval number; 17029A, 18031A, 19033A, 21002A, 21012A, and 21052A) and National Center for Child Health and Diseases (NCCHD). Marmosets (CLEA Japan) were housed in CIEA and NCCHD. The marmosets were housed in stainless steel cages (W436 x D750 x H765 mm to W910 x D750 x H2050 mm) under the following conditions: temperature, 27 °C, humidity 40%, room pressure, +20 hPa, light 12 h per day, and basic food CMS-1 (CLEA Japan).

To obtain fetal gonads (E74 and E82 ovaries and E87 testes), frozen or fresh early embryos (8-cell to morula) were transferred into recipient uteri (Takahashi et al., 2014). The developmental days were determined based on the day of ovulation when the serum progesterone of the recipient animals exceeded 10 ng/ml. Fetuses were obtained by C-sections. The pregnant female marmosets were pre-anesthetized with 0.04 mg/kg medetomidine (Domitor, Nippon Zenyaku Kogyo), 0.40 mg/kg midazolam (Dormicam, Astellas Pharma), and 0.40 mg/kg butorphanol (Vetorphale, Meiji Seika Pharma), then sedated by isoflurane (Isoflurane inhalation solution, Viatris) inhalation. While C-section, the animals were warmed on a warmer pad on the surgery table. The uterus was exteriorized via a midline laparotomy and lifted from the abdominal cavity, and the fetus was removed from the uterus. After C-section, the female marmoset's abdominal wall and skin were sutured and kept warm in the intensive care unit until awakened from anesthesia.

Testis from 22d marmosets was obtained by hemicastration under anesthesia by inhalation of 1–3% isoflurane. Newborn ovaries and adult testes were obtained from animals sacrificed for use in other experiments and for illnesses that could not be cured, respectively.

mRNA preparation

For *in vitro* transcription of *SOX17* mRNA, marmoset *SOX17* cDNA was PCR-amplified from testis cDNAs using a following set of primers: Sox17_5UTR and Sox17_R_Asc1. The PCR product was again amplified to include the human *HBA* gene 5' UTR by using a following set of primers: 5UTR_HBA_Not1 and Sox17_R_Asc1. The primer sequences are listed in Table S4. The product was digested using Asc1 and Not1 and then ligated to phBG vector (deposited in RIKEN BRC #RDB18330) (Watanabe et al., 2019) to generate phBG-SOX17 vector. phBG-SOX17 and previously generated phBG-P53DD (Addgene #149707) (Watanabe *et al.*, 2019) vectors were digested with HindIII for *in vitro* transcription using mMESSAGE mMACHINE T7 Ultra Kit (Ambion). *In vitro* transcribed mRNAs were purified using LiCl precipitation, and they were used for the transfection.

Induction of PGCLCs

iPSCs (lines 971 and 972) were induced from ear fibroblast cells as described previously (Watanabe et al., 2019). Lipofection-based mRNA transfection was performed to generate PGCLCs from iPSCs. The day before transfection (day 0), the iPSCs were dissociated into single cells by Accumax (17087-54; Nacalai Tesque) at 37 °C and then 5.0×10^4 marmoset iPSCs were plated per one well of 12-well cell culture plates in MEF-conditioned medium containing 1.25 µg/ml iMatrix-511 silk (892021; FUJIFILM) and 10 µM Y-27632 (08945-42; Nacalai Tesque). The next day (day 1), the medium was replaced with 500 µL of the new conditioned medium. For transfection, two tubes containing 62.5 µL of OPTI-MEM (A4124801; Invitrogen) were prepared. In one tube, RNAs [0.2 µg of SOX17 mRNA, 0.05 µg of P53DD mRNA, 0.075 µg of the mixture of E3, K3, B18R mRNAs (00-0076; Reprocell)] were added. In the other tube, 1.5 µL of Lipofectoamine RNAiMax Transfection Reagent (13778150; Invitrogen) was added. After mixing well, the solutions in the two tubes were mixed, and the mixture was allowed to stand for 10 min. Next, the mixture was added dropwise into one well of a 12-well plate. The next day (day 2), the medium was replaced with 500 µL of new conditioned medium, and RNA transfection was performed again. Medium change and RNA transfection was performed at 10 am and 4 pm, respectively.

On the morning of day 3 (~10 am), cells were dissociated using Accumax. Subsequently, ~5,000 cells were plated in each well of a Nunclon Spher 96-Well plate (174929; Thermo Fisher Scientific) in 100 µL of aRB27 (1% B27/2 mM L-glutamine/1× non-essential amino acids/1× antibiotic-antimycotic in advanced RPMI medium) containing 50 ng/mL EGF (236-EG-200; R&D Systems), 100 ng/mL SCF (455-MC-050; R&D Systems), 1,000 U/mL LIF (LIF1010; Merck), 400 ng/mL BMP4 (314-BP-500; R&D Systems), and 10 µM Y-27632. The medium was changed every 4 days. BrdU (30 µg/ml) was added 48 hr before the sampling.

Lentivirus preparation and infection

Lentivirus was prepared using a protocol on the RIKEN website (https://dnaconda.riken.jp/Form_PDF/lntPrepen.pdf). Briefly, CS-CA-GFP (RDB05964), pCMV-VSV-G-RSV-Rev (RDB04393), and pCAG-HIVgp (RDB04394) were co-transfected into HEK293T cells. The supernatant was collected to enrich the lentivirus using LentiX-concentrator (Takara, 631231). The virus was stored at -80ºC until use. For the infection of the virus, the virus was added to the medium together with 4 µg/ml of polybrene. Then, spinfection was performed at 800 xg for 30 minutes at 32ºC.

Knock-in of reporter gene into *SOX17* gene locus

To visualize the generation of PGCLCs, T2A-tdTomato was inserted into the C-terminus of the *SOX17* gene. To make the targeting vector, 820-bp 5' and 669-bp 3' arms were amplified from the genome and inserted into the BamHI-digested KW1521 vector using an In-fusion HD cloning kit (Takara, 639648). The KW1521 vector contains T2A-tdTomato cassette and human beta-actin promoter driven Neomycin resistant gene cassette for the selection. For generating sgRNA-expressing vectors, PCR was performed using a common primer (sgRNA-Universal-rev-primer) and specific primers (pHL_Sox17_1, pHL_Sox17_2, pHL_Sox17_3). PCR products were then digested with EcoRI and BamHI, and ligated to the EcoRI and BamHI sites of pHL-H1-ccdB-mEF1a-RiH vector (Addgene #60601) (Li et al., 2015).

For homologous recombination, iPS cells were plated at the concentration of 8×10^4 cells per one well of 6-well plate at one day before the transfection (Day 0). Transfection was performed using the following plasmid DNAs [1 µg of targeting vector, 0.5 µg of pHL-EF1a-SphcCas9-iP-A (Addgene #60599) (Li *et al.*, 2015), and 167 ng each of three sgRNA vectors (pHL_H1_Sox17_1, pHL_H1_Sox17_2, pHL_H1_Sox17_3)] using Lipofectoamine 3000 reagent (Thermo Fisher Scientific, L3000008) or Lipofectoamine stem transfection reagent (Thermo Fisher Scientific, STEM00015) according to the manufacture's protocol. The day after the transfection (Day 2), cells were newly plated onto one well of a six-well plate. From Day 3, the selection was performed using 150 µg/ml G418. Colony pickup was performed ~Day 12.

For isolation of DNA for genotyping, cells were treated with DNA isolation buffer [100 mM Tris-HCl (PH 7.6)/100 mM NaCl/10 mM EDTA (PH 8.0)/0.5% SDS] containing 100 µg/ml Proteinase K at 50°C. After the phenol-chloroform extraction, DNA was precipitated by adding an equal volume of isopropanol at room temperature. PCR was performed to detect knock-in using the following sets of primers: Sox17_scr5F and tdTomatoR1 (recombination at the coding region side), Sox17_scr3R and human-b-actProR (recombination at the 3' UTR side), and Sox17_scr5F and Sox17_scr3R (for detecting entire knock-in insertion or wildtype bands).

Karyotype analysis

The iPSCs were cultured for 3 h in a medium containing 0.1 µg/mL colcemid (15212012; Gibco) and dissociated into single cells by treatment with Accumax. After collection, the cells were treated with 0.075 M KCl for 30 min at 25°C. Subsequently, the cells were fixed using a fixing solution (70% methanol/30% acetic acid). The fixed cells were then spread onto a glass slide using HANABI and stained with Hoechst 33342 (H1399; Invitrogen). Karyotype images were obtained using a Leica 6000B microscope. All three iPSC lines used in this study had normal karyotype.

mRNA ST: 46 XX 80% (5/5)

971 STCE: 46 XY 87.5% (7/8)

972 STCE: 46 XY 87.5% (7/8)

FACS purification of PGCLCs

PGCLC aggregates were dissociated with 0.25% Trypsin-EDTA for 10 minutes at 37 °C. The reaction was stopped by adding an equal amount of 10% KSR/alpha-MEM. DNaseI was added at the final concentration of 25 µg/ml, and cells were left at room temperature for 1 minute before centrifuge. After adding FACS buffer containing DAPI for removing dead cells, cells were filtered using a 60 µm cell strainer. tdTomato-positive cells were purified using SONY SH800 using purity mode.

RT-qPCR

RNA was extracted using TRIzol Reagent (15596026; Invitrogen). RNA was converted to cDNA using Superscript IV Reverse Transcriptase VILO Master Mix with ezDNase enzyme (11766050; Invitrogen), and gene expression analysis was performed using the primers listed in Table S4.

Immunofluorescence

xrtestes collected from kidneys and testes were fixed in 4% PFA (09154-56; Nacalai Tesque), and then they were embedded into paraffin blocks. Afterward, sections (4 µm) were deparaffinized and rehydrated using a xylene and ethanol series. For antigen retrieval, slides were heated to 95 °C for 10 min in 0.01 M citrate buffer (pH 6.0). The sections were blocked using a primary antibody diluent (AR9352; Leica Biosystems) and incubated overnight at 4 °C with the following primary antibodies: Rabbit-anti-GFP (308SS; 1:1,000; Novus Biologicals), Goat-anti-GFP (ab5450; 1:3,000; Abcam), anti-LAMININ (L9393; 1:200; Sigma-Aldrich), anti-3 β -HSD (sc-515120; 1:1,500; Santa Cruz Biotechnology Inc), anti-WT1 (ab89901; 1:500; Abcam), anti-MKI67 (NCL-ki67p; 1:50; Leica Biosystems), anti-TFAP2C (sc-12762; 1:100; Santa Cruz Biotechnology Inc), anti-DDX4 (AF2030; 1:500; R&D Systems), anti-MAGEA3/4 (MABC1150; 1:250; Merck), anti-PDPN (337001; 1:200; Biolegend), anti-BrdU (Bu20a; 1:200; Dako), and anti-PIWIL4 (GP1831 produced in a guinea pig; AHSSFRATEVGRTQD-Cys; 1:50). Subsequently, the sections were incubated with species-specific secondary antibodies for 60 min at room temperature.

***In vivo* differentiation**

To isolate fetal testicular somatic cells, pregnant ICR females were sacrificed, and embryos were collected at E13.5. Fetal male testes were distinguished from ovaries by their appearance, and the mesonephros attached to the testes were removed using a tungsten needle. Thereafter, the isolated

testes were treated with 0.25% trypsin EDTA (25200056; Nacalai Tesque) for 10 min at 37 °C for dissociation into single cells. To remove endogenous mouse PGCs, cells were plated in a cell culture plate in MEM α (12571063; Gibco) containing 10% KSR (10828-028; Gibco) and cultured for 6 h (Ohinata et al., 2009). PGCs usually do not attach to plates; the attached cells were removed from the culture plate using 0.25% trypsin/EDTA and collected as fetal testicular somatic cells. The proportions of germ cells in the attached and unattached fractions were 3.3% and 69.1%, respectively (determined by *DDX4* immunostaining).

Marmoset d4 or d12_PGCLCs were purified using *SOX17*-T2A-tdTomato fluorescence and FACS. The collected d4 or d12_PGCLCs (5.0×10^3 cells) were mixed with E13.5 fetal testicular somatic cells (5.0×10^4 cells) and plated in an ultra-low attachment 96-well plate (174929; Thermo Fisher Scientific) with MEM α containing 10% KSR, 1× antibiotic-antimycotic and 10 μM Y27632. The next day, cell aggregates were transplanted under the kidney capsule of NOG (NOD/Shi-scid, IL-2RγKO) mice to promote differentiation (Ito et al., 2002). Thereafter, NOG mice were anesthetized using medetomidine, midazolam, and butorphanol, and an incision was made in the peritoneum to expose the kidney capsule. Next, the kidney capsule was carefully incised using an injection needle under an inverted microscope, and cell aggregates were picked up using a glass capillary and transplanted into the kidney capsule.

Single cell RNA-seq (10X)

After dissection, mesonephros, epididymis and tunica albuginea were removed from testes and ovaries. Testes and ovaries were cut in pieces using forceps or scalpels. The iPS cells were dissociated using Accumax, while PGCLCs, ovaries (E74, E82, and newborn), and testes (E87 and 22d) were dissociated using 0.25% trypsin EDTA for 10 min at 35°C. Cell dissociation was promoted by gentle pipetting or tapping. Adult testes (3 years 10 months) were digested stepwise with 30 min collagenase 1 (1mg/mL) treatment and 10 min trypsin (0.25%) EDTA treatment. Trypsin reaction was stopped by adding 10% FCS/DMEM. DNaseI was, then, added at the final concentration of 20 μg/ml. Newborn ovaries were not completely digested by the above procedure, while others were digested almost completely. Cell suspension were filtered through 70 μm cell strainer. Cells were then washed using 0.04% BSA/PBS three times. For iPS cells, PGCLCs, and E82 ovaries, libraries were constructed using a Chromium Next GEM Single Cell 3′ GEM Library & Gel Bead Kit v3 (10x Genomics). Other libraries were constructed using a Chromium Single Cell 3′ Library and Gel Bead Kit v2 (10x Genomics), and sequencing was performed using a HiSeq4000 system (Illumina). One library was made from each stage.

To analyze 10x data, datasets were mapped to the common marmoset genome, calJac4 (or calJac3), using CellRanger. NCBI Callithrix jacchus Annotation Release 105 was used to obtain UMI count data. The UMI count data were analyzed using Seurat. Normalization and

standardization were performed using *NormalizeData* and *ScaleData* functions. Principal component analysis was performed using the inbuilt *RunPCA* function. Dimensionality reduction was visualized in 2D using *DimPlot*.

Comparison of *in vitro* datasets with *in vivo* dataset and Human datasets

Marmoset 10X datasets were mapped to the common marmoset genome (Callithrix jacchus 3.2.1) using CellRanger 3.0.2, which was used filter out low-quality cells and empty droplets. Gene annotation files for common marmoset genome (Caljac 3.2.1) were downloaded from Ensembl (release 91) and gene models extended similarly to the approach that was reported (Bergmann et al., 2022; Boroviak et al., 2018).

For comparison with human, several existing human 10x datasets were also analyzed, including germ cell samples from neonatal testes (Sohni et al., 2019) (GSE124263) and PGCLCs induced from iPSCs (Chen et al., 2019) (GSE140021). Smart-seq2 (SS2) datasets of male and female fetal germ cells (Li et al., 2017) (GSE86146) were incorporated in downstream analyses alongside single cell data from human iPSC and PGCLCs (Kojima et al., 2017) (GSE99350).

All samples that passed QC were analyzed using Seurat v3.1.2 (Butler et al., 2018). UMI data was normalized and standardised using the *NormalizeData* and *ScaleData* function using either the top 2,000, 5000 or 20,000 most varied genes for initial analysis. Principal component analysis was run using the inbuilt *RunPCA* function, with nonlinear dimensionality reduction techniques generated using RunUMAP. Dimensionality reduction was visualised in 2D using DimPlot.

Data from marmoset and humans were incorporated together for an integrative view of germ cell development. First our 10X samples from iPSCs were combined with samples from PGCLC aggregates to create a marmoset *in vitro* reference. Each gene in the marmoset data was annotated by its human orthologue based on the Ensembl BioMart database (https://m.ensembl.org/info/data/biomart/index.html). These homologous genes were used for the integrative analyses of Human and marmoset data. The marmoset reference was integrated alongside human ESC and aggregate datasets (Chen *et al.*, 2019), consisting of two replicated cell lines, one with high PGCLC-competence and one with low PGCLC-competence. The five datasets (our dataset, two replicates high competence line in human, two replicates low competence line) were then jointly aligned in Seurat based on Canonical Correlation Analysis (CCA) and mutual nearest neighbor (MNN) approaches. Specifically, FindIntegrationAnchors was run using 4000 features and In*tegrateData* (with 20 dimensions) was used to calculate corrected gene expression matrices for the three datasets. Datasets were visualized using PCA and UMAP on the corrected gene expression matrix, with expression plots based on the uncorrected RNA slot expression. Unbiased clustering on the aligned marmoset-human dataset was done using the *FindClusters* function. Recent re-analysis of these datasets alongside

appropriate *in vivo* references (Castillo-Venzor et al., 2022) identified a number of transient and terminal cell populations in the human aggregates, including primitive-streak-like cells, mesoderm-like cells, amnion-like cells, endoderm-like cells, and PGC-like cells. Cell identity assignment in the marmoset datasets could be assigned by co-clustering with the annotated human cells.

Subsequently, marmoset iPSC and PGCLC cells were merged with 10X samples of marmoset PGCs to create a marmoset PGC developmental trajectory reference dataset, which was integrated with 10X samples from human neonate gem cell lineages merged with 10X samples of human iPSCs and PGCLCs. SS2 datasets from male and female germ cells were merged with SS2 samples of PGCLCs and iPSCs. Unbiased clustering on the aligned marmoset-human dataset was done using the *FindClusters* function, and individual clusters were annotated based on the dominant lineages in the cluster. Cell lineages for marmosets were first identified in individual samples based on the expression of known marker genes (Figure S3). These annotations were additionally checked against the aligned human annotations and showed strong agreement.

Finally, marmoset lineages were appended by developmental stage, and the average expression of lineages was calculated using *AverageExpression*. Row normalized expression values for an extended panel of known markers was visualized using pheatmap.

RNA and DNA purification from the same single cell

xrtestes were cut in pieces using scalpels and they were dissociated using 0.25% Trypisn-EDTA. xrtestis cells were resuspended in 0.04% BSA in PBS for single-cell pick up. Single-cell (EGFP+) was picked up by a mouth pipet and put into a PCR tube. Lysis buffer (1.14 U/µl Rnase Inhibitor, 0.54% Triton X-100) containing DynaBeads MyOne Carboxylic acid (Thermo Fisher) was added. Beads bound to the nucleus and supernatant corresponding to the cytoplasmic fraction were separated. DNA isolation buffer (20 mM Tris-EDTA, 20 mM KCl, 0.3% Triton X-100, 1mg/ml Proteinase K, 0.1 pg/µl λDNA, 2 ng/µl carrier RNA) was added to the beads, and they were incubated at 50°C for 10 minutes. Proteinase was then heat-inactivated by 75°C for 30 minutes.

Single-cell RNA-seq of the cytoplasmic fraction

Before cDNA synthesis, ERCC RNA (Invitrogen, 1:500,000) was added to the cytoplasmic RNA. cDNA was made from cytoplasmic RNAs using oligo dT primer with cell barcode and UMI sequences (CB16UMI12-RT), template switch (TS) oligo DNA, and SuperScript II Reverse transcriptase (Thermo Fisher). To prevent concatamerization of the TS oligo, non-natural nucleotides were added to its 5' end. PCR reaction was performed using 3' Anchoerd primer and ISPCR primers to amplify cDNA. After purification of cDNA using AMPure beads, the qPCR reaction was carried out using a primer pair that detected germ cell-specific genes (NANOS3 *and*

PIWIL4) for the selection of germ cell. After pooling 8 cDNAs with different barcodes, PCR was performed using a biotinylated primer bound to 5' end of oligodT sequence (Biotin-index-primer) and unmodified primer annealed to 5' end of TS oligo (ISPCR). Fragmentation was carried out using NEBNext Ultra II FS DNA Library Prep Kit for Illumina, and biotinylated DNA was purified using streptavidin beads. Adapter ligation and PCR-amplification (using QP2 and Modified_P5_primer) were then performed according to NEBNext Ultra II FS DNA Library Prep Kit for Illumina. After quality check using Bioanalyzer (Agilent), paired-end 150-bp sequencing was performed by Illumina HiSeqX. Primer sequences are listed in Table S4.

Single-cell BS-seq

Single-cell BS-seq was conducted based on published literatures (Bian et al., 2018; Smallwood et al., 2014; Zhou et al., 2019). Before the bisulfite reaction, non-methylated lamda DNA (Promega) was added to the DNA. For bisulfite conversion, MethylCode Bisulfite Conversion kit (Invitrogen) was used. For making single-cell BS-seq libraries, two rounds of random priming reaction were performed using a random primer with a nucleotide ratio of A:T:G:C = 4:4:1:1 (scBS-P5-N9-oligo1), which reduces bias toward preferential amplification of GC-rich sequences. After ExonucleaseI treatment for removing the remaining random primer, second-strand synthesis was performed using scBS-oligo2 primer. PCR amplification were then carried out using unique dual index primers (NEBNext Multiplex Oligos for Illumina, 96 Unique Dual Index). After quality check using a bioanalyzer (Agilent), paired-end 150-bp sequencing was performed by Illumina HiseqX.

scRNA/scBS-seq data analysis

Our custom scRNA libraries were designed for analyses using CellRanger. Prior to the analyses using CellRanger, Read1 and Read2 were switched by changing the file names. Datasets were mapped to the common marmoset genome calJac4 using CellRanger. For annotation of calJac4 genome, NCBI Callithrix jacchus Annotation Release 105 was used. UMI count data were analyzed using Seurat. The count matrix was created by *CreateSeuratObject* function. To extract the cells of interest, a *subset* function was used. Data from different libraries were combined using the *merge* function. To remove cells with a small number of UMIs, a subset function was used by setting a cutoff value of unique feature counts over 1500. Normalization and standardization were performed using the *NormalizeData* and *ScaleData* functions, respectively. Appropriate cell names were reassigned using the *changeindent* function. Variable genes were extracted by *FindVariableFeatures* and principal component analysis was run using the inbuilt *RunPCA* function. Heatmap was generated using a *pheatmap* package.

For scBS-seq analysis, quality of the library was checked by FastQC and trimmed away adaptor

sequence using the TrimGalore program using an option --quality 20 --stringency 3 --length 50 --clip_R1 9 --clip_R2 9 --paired --trim1 --phred33. Reads were mapped to the reference genome using Bismark with a command bismark --bowtie2 --fastq --non_directional --un. Duplicate reads were removed with *deduplicate* function in Bismark (deduplicate_bismark –bam). Deduplicated BAM files were then converted to bedGraph using *methylation extractor* (bismark_methylation_extractor -p --bedGraph -counts).

For retrotransposon analyses, the coordinates of 8,251 full-length LINE1 and 2,275 full length Platy-1 elements were retrieved from L1 base2 (l1base.charite.de/l1base.php) and a published literature(Konkel et al., 2016), respectively. The calJac3 coordinates were converted to calJac4 coordinates using a liftover tool in UCSC (Lift Genome Annotations (ucsc.edu)). The coordinates for three major retrotransposon classes were retrieved from repeatmasker file downloaded from UCSC. Methylation data in bedgraph file format were used to calculate the average methylation levels.

Supplemental References

Bergmann, S., Penfold, C.A., Slatery, E., Siriwardena, D., Drummer, C., Clark, S., Strawbridge, S.E., Kishimoto, K., Vickers, A., Tewary, M., et al. (2022). Spatial profiling of early primate gastrulation in utero. Nature. 10.1038/s41586-022-04953-1.

Bian, S., Hou, Y., Zhou, X., Li, X., Yong, J., Wang, Y., Wang, W., Yan, J., Hu, B., Guo, H., et al. (2018). Single-cell multiomics sequencing and analyses of human colorectal cancer. Science *362*, 1060-1063. 10.1126/science.aao3791.

Boroviak, T., Stirparo, G.G., Dietmann, S., Hernando-Herraez, I., Mohammed, H., Reik, W., Smith, A., Sasaki, E., Nichols, J., and Bertone, P. (2018). Single cell transcriptome analysis of human, marmoset and mouse embryos reveals common and divergent features of preimplantation development. Development *145*. 10.1242/dev.167833.

Butler, A., Hoffman, P., Smibert, P., Papalexi, E., and Satija, R. (2018). Integrating single-cell transcriptomic data across different conditions, technologies, and species. Nat Biotechnol *36*, 411-420. 10.1038/nbt.4096.

Castillo-Venzor, A., Penfold, C.A., Morgan, M.D., Tang, W.W.C., Kobayashi, T., Wong, F.C.K., Bergmann, S., Slatery, E., Boroviak, T.E., Marioni, J.C., and Surani, M.A. (2022). Origin and segregation of the human germline. bioRxiv, 2022.2007.2006.498671. 10.1101/2022.07.06.498671.

Chen, D., Sun, N., Hou, L., Kim, R., Faith, J., Aslanyan, M., Tao, Y., Zheng, Y., Fu, J., Liu, W., et al. (2019). Human Primordial Germ Cells Are Specified from Lineage-Primed Progenitors. Cell Rep *29*, 4568-4582 e4565. 10.1016/j.celrep.2019.11.083.

Ito, M., Hiramatsu, H., Kobayashi, K., Suzue, K., Kawahata, M., Hioki, K., Ueyama, Y., Koyanagi, Y., Sugamura, K., Tsuji, K., et al. (2002). NOD/SCID/gamma(c)(null) mouse: an excellent recipient mouse model for engraftment of human cells. Blood *100*, 3175-3182. 10.1182/blood-2001-12-0207.

Kojima, Y., Sasaki, K., Yokobayashi, S., Sakai, Y., Nakamura, T., Yabuta, Y., Nakaki, F., Nagaoka, S., Woltjen, K., Hotta, A., et al. (2017). Evolutionarily Distinctive Transcriptional and Signaling Programs Drive Human Germ Cell Lineage Specification from Pluripotent Stem Cells. Cell Stem Cell *21*, 517-532 e515. 10.1016/j.stem.2017.09.005.

Konkel, M.K., Ullmer, B., Arceneaux, E.L., Sanampudi, S., Brantley, S.A., Hubley, R., Smit, A.F., and Batzer, M.A. (2016). Discovery of a new repeat family in the Callithrix jacchus genome. Genome Res *26*, 649-659. 10.1101/gr.199075.115.

Li, H.L., Fujimoto, N., Sasakawa, N., Shirai, S., Ohkame, T., Sakuma, T., Tanaka, M., Amano, N., Watanabe, A., Sakurai, H., et al. (2015). Precise correction of the dystrophin gene in duchenne muscular dystrophy patient induced pluripotent stem cells by TALEN and CRISPR-Cas9. Stem Cell Reports *4*, 143-154. 10.1016/j.stemcr.2014.10.013.

Li, L., Dong, J., Yan, L., Yong, J., Liu, X., Hu, Y., Fan, X., Wu, X., Guo, H., Wang, X., et al. (2017). Single-Cell RNA-Seq Analysis Maps Development of Human Germline Cells and Gonadal Niche Interactions. Cell Stem Cell *20*, 858-873 e854. 10.1016/j.stem.2017.03.007.

Ohinata, Y., Ohta, H., Shigeta, M., Yamanaka, K., Wakayama, T., and Saitou, M. (2009). A signaling principle for the specification of the germ cell lineage in mice. Cell *137*, 571-584. 10.1016/j.cell.2009.03.014.

Pauls, K., Jager, R., Weber, S., Wardelmann, E., Koch, A., Buttner, R., and Schorle, H. (2005). Transcription factor AP-2gamma, a novel marker of gonocytes and seminomatous germ cell tumors. Int J Cancer *115*, 470-477. 10.1002/ijc.20913.

Smallwood, S.A., Lee, H.J., Angermueller, C., Krueger, F., Saadeh, H., Peat, J., Andrews, S.R., Stegle, O., Reik, W., and Kelsey, G. (2014). Single-cell genome-wide bisulfite sequencing for assessing epigenetic heterogeneity. Nat Methods *11*, 817-820. 10.1038/nmeth.3035.

Sohni, A., Tan, K., Song, H.W., Burow, D., de Rooij, D.G., Laurent, L., Hsieh, T.C., Rabah, R., Hammoud, S.S., Vicini, E., and Wilkinson, M.F. (2019). The Neonatal and Adult Human Testis Defined at the Single-Cell Level. Cell Rep *26*, 1501-1517 e1504. 10.1016/j.celrep.2019.01.045.

Takahashi, T., Hanazawa, K., Inoue, T., Sato, K., Sedohara, A., Okahara, J., Suemizu, H., Yagihashi, C., Yamamoto, M., Eto, T., et al. (2014). Birth of healthy offspring following ICSI in in vitro-matured common marmoset (Callithrix jacchus) oocytes. PLoS One *9*, e95560. 10.1371/journal.pone.0095560.

Watanabe, T., Yamazaki, S., Yoneda, N., Shinohara, H., Tomioka, I., Higuchi, Y., Yagoto, M., Ema, M., Suemizu, H., Kawai, K., and Sasaki, E. (2019). Highly efficient induction of primate iPS cells by combining RNA transfection and chemical compounds. Genes Cells *24*, 473-484. 10.1111/gtc.12702.

Zhou, F., Wang, R., Yuan, P., Ren, Y., Mao, Y., Li, R., Lian, Y., Li, J., Wen, L., Yan, L., et al. (2019). Reconstituting the transcriptome and DNA methylome landscapes of human implantation. Nature *572*, 660-664. 10.1038/s41586-019-1500-0.
